# Supplementary material for: Short-term occupations at high elevation during the Middle Paleolithic at Kalavan 2 (Republic of Armenia)
Source: PLoS One. 2021 Feb 4;16(2):e0245700. doi: 10.1371/journal.pone.0245700 (PMC7861461; doi:10.1371/journal.pone.0245700)
Supplement: S1 File — (DOCX) [file pone.0245700.s001.docx]

**S1 section.**

## Kalavan 2 previous C14 dating

Radiocarbon dating was attempted on four samples of Kalavan 2 archaeological material: two in Poznan and two in the Center of Applied Isotope Studies at the University of Georgia [1]. One bone sample examined from Layer 19 gave a date of 42,040 ± 400 BP (UGAMS-2295). From Layer 7, two fragments of the same bovid teeth (Poz-20366 and Poz-22181) yielded age estimates of 34,200 ± 360 BP and 27,000 ± 400 BP. The variance in the results from the same bone is most likely a result of lab contamination. From Unit 6/7 an equid tooth was sampled twice (UGAMS-296 and UGAMS 2296a), once for collagen and once for bioapatite dating. The two samples gave a late age of 16,740 ± 130 BP uncalibrated and the bioapatite sample one of 20,000 ± 100 BP uncalibrated. Thus, the collagen date yielded more than 3000 ^14^C years younger age than the bioapatite one. [2]. These results contrast the results obtained from a sample from Layer 19 where the collagen fraction gave an older 14C age than the bioapatite one. This reversal in ages in the different specimens between the two dating methods was explained by the dissimilarities in the depositional environment between Layers 19 and 6 and the changes in the past water table at the locality. Thus, isotope exchange between bone bioapatite and groundwater-dissolved inorganic carbon compounds could have occurred in the different layers and therefore affected the bone collagen preservation and chemistry [2].

## OSL methodology

Luminescence samples were taken using light-tide steel tubes, material for high-resolution laboratory gamma-spectrometry was taken at the same positions as material for luminescence dating.

Sample preparation for luminescence dating was conducted under subdued red light in the OSL laboratory at the Max Planck Institute for Evolutionary Anthropology in Leipzig, Germany. To test the luminescence properties of the coarse-grain (180-250 µm) and fine grain (4-11 µm) material, both grain-size fractions were prepared. The fine-grain fraction was isolated by conducting several runs of centrifugation, the coarse fraction was obtained by sieving.

Sample preparation also included the removal of carbonates and organic matter by using HCL (15%) and hydrogen peroxide (30%), respectively. The coarse-grain feldspar fraction was isolated from heavy-minerals and quartz using lithium heterotung state.

**Equivalent dose (De) estimation and dose rate**

Due to the high proportion of volcanic rocks in the sedimentary environment, feldspar was used for OSL dating as it was demonstrated in previous studies, that volcanic quartz often yields problematic luminescence characteristics [3].

All luminescence measurements were conducted on a Risø TL/OSL DA-20 reader equipped with a 90Sr/90Y beta source with a dose rate of about 0.12 Gy/s.

Feldspar-stimulation was conducted using IR-diodes (~870nm) and the IRSL signal was detected in the320–480 nm wavelength-region.

Tests on the coarse (180-250 microns) feldspar material showed a very low luminescence sensitivity of the coarse feldspar material excluding the possibility of using small-sized aliquots or single grains for feldspar-luminescence dating. The fine-grain material yielded much brighter luminescence and reproducibility tests on polyminerals underlined the suitability of the 4-11 µm fraction for dating (see Fig. Sp 1. Fig. 1 and 2).

The protocol parameters for the used single-aliquot regenerative dose (SAR) protocol were defined by applying dose recovery tests on samples L-Eva 1684, 1685, and 1686. It turned out that the pIRIR225 [4] approach is most suitable for equivalent dose measurements. In the used protocol, the feldspar signal was measured at 225°C for 200 s after depleting the IR50 signal for 100 s. The preheat/ cutheat temperature was set to 250 °C. From each sample, 10-15 aliquots were measured. For final De-calculation, only aliquots with recycling ratios deviating < 10 % from unity were integrated. The De-value from each sample was calculated by determining the mean-value and its standard error respectively.

The conducted fading measurements [5] indicated the high signal stability (low fading) of the feldspar signal stimulated at 225°C after depleting the IR50 signal. Therefore, no fading correction was conducted.

To obtain the nuclide concentrations of 238U, 232Th, and 40K and their daughter nuclides high-resolution gamma spectrometry was conducted in the Felsenkeller-laboratory in Dresden. The cosmic dose rate was taken into account by considering longitude/altitude, height a.s.l. and thickness of the covering sediment layer [6], dose rate conversion factors following Guerin et al. [7] were used. Results of equivalent dose measurements and dosimetry are shown in table 5.

Tephra methods

Contiguous 2 cm thick samples of sediment were removed from cleaned trench sections and placed into individual zip-lock bags. In the laboratory, five 2 cm thick samples were amalgamated to create a 10 cm thick scan sample, measuring approximately 2-3 cm^3^. These were dried overnight at 105°C, before being combusted at 550°C to eliminate any organics. The residues from this process were immersed in 10% HCL to remove any carbonate, with the remaining material passed through sieves with an aperture of 125 and 15 μm: retaining all residue greater than 15 μm in size. Sieved material in the 15-125 μm size range was processed following the Blockley et al., [8] flotation method, to extract and isolate any volcanic glass shards from the host substrate. To quantify glass shard content, samples were spiked with a known concentration of palynospheres before being mounted on glass microscope slides using Canada balsam as the mounting medium [9]. Quantification of glass shard content was conducted using a high-powered polarizing light microscope at 200 and 400 x magnification, and concentrations of glass shards were established using the following equation:

$$C=\left( \frac{pxa}{b} \right)d$$

Where c = concentration of glass shards, p = number of palynospheres in a given volume, a = glass shard count, b = palynosphere count, d = dry sample weight in grams. Where the highest concentrations of glass shards were identified, the corresponding 2 cm bag samples comprising the 10 cm san sample, were re-sampled and processed to establish the precise interval where concentrations were highest. Those corresponding peaks in glass shard concentration, along with the visible volcanic ash layers identified in the Kalavan 2 trench excavations, were selected for major element characterization by Electron Microprobe Analysis (EPMA). Visible volcanic ash samples were bulk mounted onto silicon sheets and impregnated in epoxy resin, whereas cryptotephra glass shards were isolated from their host sediments using the same extraction procedure outline above (omitting the combustion stage) before impregnation. Once hardened, the epoxy resin ‘stubs’ containing the tephra were sectioned, exposing the glass shards at the surface, and polished using 0.3 μm Aluminium oxide powder. Major element analyses of the glass shards were conducted at the WDS-EPMA (Cameca SX-100) facility in Edinburgh. Beam diameter was set at 5 μm and 15 keV; a current of 2 nA was employed for Al, Si, Fe, K, Ca, Mg and a beam current of 80 nA for F, Mn, Cl and Ti [10]. To avoid beam-induced chemical modification and depletion of alkalis, various beam currents and count times were used to test for, and ultimately prevent Na mobilization. Settings of 0.5-1 nA and count times of 10-20 seconds for Na produced similar results to analyses conducted using higher currents; (2 nA) and count times of 20 seconds, suggesting Na mobilization was not a major concern for these samples. Establishing the potential susceptibility for Na mobilization is particularly important due to the lack of published grain specific glass analytical data available from sites in the Southern Caucasus. The electron microprobe was calibrated and analytical precision assessed by the analysis of internal Lipari and BCR-2G secondary standards.

***Reference:***

1. Ghukasyan, R., Colonge, D., Nahapetyan, S., Ollivier, V., Gasparyan, B., Monchot, H., Chataigner C. Kalavan-2 (North of Lake Sevan, Armenia): A new late middle paleolithic site in the Lesser Caucasus. Archaeol Ethnol Anthropol Eurasia. 2011;38: 39–51. doi:10.1016/j.aeae.2011.02.003

2. Cherkinsky A, Chataigner C. 14 C Ages of Bone Fractions from Armenian Prehistoric Sites . Radiocarbon. 2010;52: 569–577. doi:10.1017/s0033822200045604

3. Tsukamoto S, Murray AS, Huot S, Watanuki T, Denby PM, Bøtter-Jensen L. Luminescence property of volcanic quartz and the use of red isothermal TL for dating tephras. Radiat Meas. 2007;42: 190–197. doi:10.1016/j.radmeas.2006.07.008

4. Buylaert JP, Murray AS, Thomsen KJ, Jain M. Testing the potential of an elevated temperature IRSL signal from K-feldspar. Radiat Meas. 2009;44: 560–565. doi:10.1016/j.radmeas.2009.02.007

5. Huntley, D.J., Lamothe M. Ubiquity of anomalous fading in K-feldspars and the measurement and correction for it in optical dating. Can J Earth Sci. 2001;38: 1093–1106. doi:10.1139/cjes-38-7-1093

6. Prescott JR, Hutton JT. Cosmic ray contributions to dose rates for luminescence and ESR dating: Large depths and long-term time variations. Radiat Meas. 1994;23: 497–500. doi:10.1016/1350-4487(94)90086-8

7. Guérin, G., Mercier, N., Adamiec G. Dose-rate conversion factors: update. Anc TL. 2011;29: 5–8.

8. Blockley, S.P.E., Pyne-O’Donnell, S.D.F., Lowe, J.J., Matthews, I.P., Stone, A., Pollard, A.M., Turney, C.S.M. and Molyneux EG. A new and less destructive laboratory procedure for the physical separation of distal glass tephra shards from sediments. Quat Sci Rev. 2005;24: 1952–1960.

9. Gehrels, M.J., Newnham, R.M., Lowe, D.J., Wynne, S., Hazell, Z.J., Caseldine C. Towards rapid assay of cryptotephra in peat cores: review and evaluation of various methods. Quat Int. 2008;178: 68–84.

10. Hayward C. High spatial resolution electron probe microanalysis of tephras and melt inclusions without beam-induced chemical modification. The Holocene. 2012;22: 119–125. Available: 119-125
