# Supplementary material for: Short-term occupations at high elevation during the Middle Paleolithic at Kalavan 2 (Republic of Armenia)
Source: PLoS One. 2021 Feb 4;16(2):e0245700. doi: 10.1371/journal.pone.0245700 (PMC7861461; doi:10.1371/journal.pone.0245700)
Supplement: S2 File — (DOCX) [file pone.0245700.s002.docx]

**S2 section- Sedimentary and paleoenvironmental proxies’ methodologies**

# **Sedimentary pXRF**

The sediments were preliminarily dried at 60°C for 48 h. The elemental analysis was done using a field-portable X-Ray fluorescence unit (pXRF) Olympus Vanta C series using Energy-dispersive X-ray fluorescence (ED-XRF) with geochemical calibration under 2-beam soil mode, measuring the Mg, Al, Si, Ca, S, P, Ti, V, Cr, Mn, Fe, Co, Ni, Cu, W, Zn, Hg, As, Pb, Bi, Se, Th, U, Rb, Sr, Y, Zr, Nb, Mo, Ag, Cd, Sn, and Sb elements. The goal was to define the distinct elemental signature of each of the Barepat and Dany Rivers watersheds inside the sedimentary sequences.

# **Methodology of Micromorphology analysis**

The micromorphological analyzes were carried out on a series of 8 thin sections of size 6 x 9 cm made by the Thomas Beckmann Laboratory (Germany) using the standard and protocol for microbiological study [1]. The description and interpretation of the micromorphological features were made using the methodology and principles of analyses notably used by [2–9].

Observation was made with an Olympus SZ7 microscope/binocular as well as a digital camera microscope dinolite AM4515ZT-Edge in order to better navigate and define with different focal lengths and representations (ocular vs. screen) all the thin sections surfaces. A successive magnification of x35 then x210 has been systematically applied in order to establish the complete characteristics of the selected samples at different scales.

# **Methodology of Pollen analysis**

Pollen extraction in archeological/soil deposits requires large sampling and several concentration steps. 22 samples of 80g on average were treated successively using HCl and HF, then successively concentrated using 10µm mesh sieving and density liqueur separation (ZnCl_2_), before glycerine addition and slide mounting. Pollen slides were analyzed under a light microscope at a 400x magnification. Pollen types were identified according to reference atlases of [10] and [11].

## n-alkane Analysis methods

After collection, bulk samples were kept frozen to avoid any post-collection alteration of the *n*-alkanes present in the samples [12]. Sediment samples were placed in a Soxhlet extractor with 400 mL of 2:1 (v/v) dichloromethane: methanol and extracted for 48 hours. Total lipid extract was evaporated under a gentle stream of N_2_ gas at 40°C before undergoing silica gel column chromatography. We eluted the aliphatic fraction with 2 mL hexane, the aromatic fraction with 4 mL dichloromethane, and the polar fraction with 4 mL methanol. When *n*-alkenes were identified as co-eluting in samples, neutral fractions were passed through a silver nitrate column to remove them. In samples with a high unresolved complex matrix, urea adduction was used to remove branched- and cyclic-alkanes.

We quantified *n*-alkanes on a Thermo-Scientific Trace GC Ultra with a split/splitless injector and flame ionization detector. Samples were separated on a BP-5 column (30m x 0.25 mm x 0.25 um) with helium as the carrier gas (1.5 ml/min). The oven temperature was set at 50°C for 1 minute, ramped to 180°C at 12°C/min, then ramped to 320°C at 6°C/min and held for 4 minutes. *n*-alkane standards of known concentration were run between every eight samples to ensure reproducibility and quantify unknown archaeological *n*-alkane concentrations.

*n*-alkanes (*n*C_25_-*n*C_35_) are found in the waxy coating of higher plant tissue, which protects the plant from water loss, microbial attack, and UV damage [13,14]. Given that *n*-alkanes are resistant to degradation over long time scales [15] and their response to environmental variables, they are therefore a useful paleoclimate proxy. Studies have demonstrated that the relative abundance of *n*-alkanes produced by plants changes in response to environmental variables such as temperature, humidity, and vapor pressure deficit [16–18]. Previous work at Middle Paleolithic sites have used these molecular proxies in direct association with archaeological materials to interpret paleoclimate [19,20]. As well as physiological changes in plants, different plant functional types (i.e. grasses vs trees) produce different chain lengths of *n*-alkanes. This difference can be measured with the average chain length (ACL) of *n*-alkanes:

$ACL=\frac{27\times C_{27}+29\times C_{29}+31\times C_{31}+33\times C_{33}}{C_{27}+C_{29}+C_{31}+C_{33}}$ (Eq. 1)

Additionally, since terrestrial plants produce n-alkanes with a strong odd-over-even predominance (OEP), post-depositional alteration can be quantified:

$OEP=\frac{C_{27}+C_{29}+C_{31}+C_{33}}{C_{26}+C_{28}+C_{30}+C_{32}}$ (Eq. 2)

**Reference:**

1. Murphy CP. Thin section preparation of soils and sediments. Berkhamsted: AB Academic Publishers; 1986.

2. Kubiëna W.L. Micropedology. Ames, Iowa: Collegiate Press; 1938.

3. Jongerius A. Soil Micromorphology. Soil Sci. 1965;99.

4. Mücher HJ. Toepassing van de micromorfologie in het geomorfologisch onderzoek. Landbouwkd Onderz. 1973;85: 439–442.

5. R. B. Fabric and Mineral Analysis of Soils. Kriege: Huntington; 1976.

6. FitzPatrick EA. Micromorphology of Soils. London: Chapman and Hall; 1984.

7. Bullock, P., Federoff, N., Jongerius, A., Stoops, G., Tursina T. Handbook for soil thin section description. Albrighton: Waine Research Publications; 1985.

8. Stoops, G. . S. Guidelines For Analysis and Description of Soil and Regolith Thin Sections. Madison: Soil Science Society of American; 2003.

9. Stoops, G., Marcelino, V., Mees, F. erpretation of Micromorphological Features of Soils and Regoliths. Amsterdam: Int Elsevier; 2010.

10. Reille M. Pollen et spores d’Europe et d’Afrique du nord. Supplement 1. Review of Palaeobotany and Palynology. Laboratoire de Botanique historique et Palynologie Université d’Aix-Marseille; doi:10.1016/s0034-6667(97)00005-5

11. Beug HJ. Leitfaden der Pollenbestimmung für Mitteleuropa und angrenzende Gebiete. München: Pfeil; 2004.

12. Brittingham A, Hren M, Hartman G. Microbial alteration of the hydrogen and carbon isotopic composition of n-alkanes in sediments. Org Geochem. 2017;107: 1–8. doi:10.1016/j.orggeochem.2017.01.010

13. Jetter R, Kunst L, Samuels AL. Composition of plant cuticular waxes. In: Riederer M, Miller C, editors. Biology of the Plant Cuticule. Oxford: Blackwell Publishing Ltd; 2006. pp. 145–181.

14. Eglinton G, Hamilton RJ. Leaf Epicuticular Waxes. Science (80- ). 1967;156: 1322–1335. doi:DOI: 10.1126/science.156.3780.1322

15. Schimmelmann A, Lewan MD, Wintsch RP. D/H isotope ratios of kerogen, bitumen, oil, and water in hydrous pyrolysis of source rocks containing kerogen types I, II, IIS, and III. Geochim Cosmochim Acta. 1999;63: 3751–3766. doi:10.1016/S0016-7037(99)00221-5

16. Bush RT, McInerney FA. Leaf wax n-alkane distributions in and across modern plants: Implications for paleoecology and chemotaxonomy. Geochim Cosmochim Acta. 2013;117: 161–179. doi:10.1016/j.gca.2013.04.016

17. Bush RT, McInerney FA. Influence of temperature and C4 abundance on n-alkane chain length distributions across the central USA. Org Geochem. 2015;79: 65–73. doi:10.1016/j.orggeochem.2014.12.003

18. Eley YL, Hren MT. Reconstructing vapor pressure deficit from leaf wax lipid molecular distributions. Sci Rep. 2018;8: 1–8. doi:10.1038/s41598-018-21959-w

19. Krajcarz MT, Gola MR, Cyrek KJ. Preliminary suggestions on the Pleistocene palaeovegetation around the Bisnik Cave (Czestochowa Upland, Poland) based on studies of molecular fossils from cave sediments. Stud Quat. 2010;27: 55–61.

20. Galván B, Hernández CM, Mallol C, Mercier N, Sistiaga A, Soler V. New evidence of early Neanderthal disappearance in the Iberian Peninsula. J Hum Evol. 2014;75: 16–27. doi:10.1016/j.jhevol.2014.06.002
