# Supplementary material for: Short-term occupations at high elevation during the Middle Paleolithic at Kalavan 2 (Republic of Armenia)
Source: PLoS One. 2021 Feb 4;16(2):e0245700. doi: 10.1371/journal.pone.0245700 (PMC7861461; doi:10.1371/journal.pone.0245700)
Supplement: S3 File — (DOCX) [file pone.0245700.s003.docx]

**S3 -**

**Use-wear methodology:**

Before the microscopic analysis, all artifacts were cleaned using distilled water in an ultrasonic bath for ten minutes. The study followed different scales of analysis, visual and macro assessment on tool edge and surface preservation, documentation of the edge modification, including retouch and major fractures, and micro-wear analysis [1]. A small sample was selected to assess the level of preservation of micro use-wear traces on the tool’s surface. In this sample, artifacts representing the different techno-typological classes were selected. Only artifacts with preserved edges and macroscopically showing no major post-depositional alterations were also included.

High-resolution macroscopic (low magnification) images for documentation and analysis were done using a digital camera (Nikon DSLR camera, model D610 with a Nikon AF-S VR Micro-Nikkor 105 mm f/2.8G IF-ED lens.) and a 3D digital automated microscope ZEISS Smartzoom 5 (equipped with a PlanApo 1.6×/0.1 objective, and an integrated segmented LED ringlight). When needed, extended depth focus and stitching methods were used to generate photos of the analyzed artifact.

After the acquisition, when needed digital images (including overviews, areas of interest, and particular macro features) were edited using GIMP (free open-source image editor, available at https://www.gimp.org/, v.2.10.18). Vectorized schematic drawing of the tools’ engravings were processed using Inkscape (free and open-source vector graphics editor, available at https://inkscape.org/, v.0.92.4). The Structure from Motion (SfM) photogrammetry models were generated from 2D digital images and using the software AgiSoft MetaShape (v.1.6.2.).

Micro-wear studies on obsidian materials are still rare when compared with other raw materials, such as flint or quartzite (see exception [2,3]. Here we follow the terminology commonly used to document microscopic use-wear traces on obsidian [2,3], using the upright light microscope ZEISS Axio Scope.A1 MAT, using the objectives EC Epiplan 10x/0.25 and 20x/0.4 for screening the tools surface, analysis, and documentation. All final images were processed using the ZEN 2 core (v. 2.XX) software and the integrated Extended depth of focus module.

**Use-wear methodology of the pitted stones:**

The methods used for the study of these materials are based on two main steps: a) multi-scale observations and b) 3D quantitative data acquisition. The multi-scale approach consists of a gradual analysis from the macroscopic to microscopic scales. Wear traces were identified and documented following the common terminology on ground stone tools studies [4–7]. Use-wear traces were organized in four main categories: residues, abrasive (e.g. striations), impact (surface macro fractures), and micro polish (i.e. sheen)

The artifacts were 3D scanned in order to quantitatively analyze their surface topography. At this phase, we present the general features of the artifacts.

Before the cleaning process, each artifact was sampled 3 times with a pipette for future residue analyses, using distilled water. After sampling, the artifacts were cleaned using distilled water in an ultrasonic bath for ten minutes, and another sample was taken from the cleaning water for residue analyses. After drying, the artifacts were 3D scanned using an HP 3D Structured Light 3D-Scanner Pro S2 (0.06 mm resolution).

**Reference:**

1. Marreiros, J. M., Bao, J. F. G., Bicho NF. Use-wear and residue analysis in archaeology. London: Springer; 2015.

2. Hurcombe L. Use wear analysis and obsidian: theory, experiments and results. Sheffield. Sheffield: Sheffield: JR Collis Publications; 1992.

3. Walton DP. An Experimental Program for Obsidian Use-Wear Analysis in Central Mexican Archaeology. J Archaeol Method Theory. 2018. doi:10.1007/s10816-018-9398-7

4. Adams JL. Ground Stone Analysis: A technological approach. Salt Lake City: The University of Utah press.; 2002.

5. Adams JL. Ground stone use-wear analysis: a review of terminology and experimental methods. J Archaeol Sci. 2014;48: 129–138.

6. Dubreuil L, Savage D. Ground stones: a synthesis of the use-wear approach. J Archaeol Sci. 2014;48: 139–153. doi:10.1016/j.jas.2013.06.023

7. Dubreuil L, Savage D, Delgado-Raack S, Plisson H, Stephenson B, de la Torre I. Current Analytical Frameworks for Studies of Use–Wear on Ground Stone Tools. 2015. pp. 105–158. doi:10.1007/978-3-319-08257-8_7
