# Supplementary material for: Short-term occupations at high elevation during the Middle Paleolithic at Kalavan 2 (Republic of Armenia)
Source: PLoS One. 2021 Feb 4;16(2):e0245700. doi: 10.1371/journal.pone.0245700 (PMC7861461; doi:10.1371/journal.pone.0245700)
Supplement: S4 File — (DOCX) [file pone.0245700.s004.docx]

# Faunal analysis methodology

Bone cleaning procedures

All faunal material was carefully cleaned with clear water and with or without a selection of soft brushes, depending on the preservation, to remove dirt and carbonate concretions as much as possible without damaging the bones. Cleaning took place off-site, in the field lab. Excluded from this process were teeth that seemed suitable for macro-and micro-wear analysis (see below).

Identification and counting

The identification of the bones is based on the comparative collection of the archaeological research center Monrepos, Neuwied. The taphonomic features were analyzed microscopically in the TraCEr lab at Monrepos. Some more completely preserved equid teeth were subject to macro-wear analysis.

The counting methods are the *Number of Identified Specimens*, or NISP, following Lyman who defines it as the “number of skeletal elements (bones and teeth) and fragments thereof” that could be identified to the taxon they represent [1]. And related to this, the *Number of Specimens* or NSP that include bones, teeth, and fragments thereof that can be identified to a specific taxon (NISP) but additionally includes the specimens that cannot. These counts are used to estimate a relative frequency of taxa and in the end, help to interpret the overall fragmentation of the assemblage [2–4].

Besides NSP and NISP, MNI will be used to define the smallest number of individuals that are necessary to account for all of the skeletal elements of a particular species found in the site. Also, the Minimal Number of Elements (MNE) was calculated as well [2–4].

**Bone surface modifications**

As mentioned above, the microscopical analysis of bone surface modifications was executed in the TraCEr lab. The 3D digital microscope ZEISS Smartzoom 5 helped not only with the recognition and distinction of surface modifications but also with taking detailed pictures of features on the bones. For simpler tasks, such as initial identification of features in lower magnification, the Upright light microscope ZEISS Axio Lab.A1 provided sufficient magnification and utility.

With this equipment, cut marks were able to be told apart from trampling marks, gnawing, and other taphonomic characteristics [5–7].

Features made by anthropogenic agents, such as butchery marks or patterns were examined and distinguished following Binford [8] and Lyman [3].

Other biostratinomic factors, like weathering, root etching, and burning, but also diagenetic features such as mineralization and other chemical alterations of the bones were also identified following Lyman [3].

Since a lot of the specimens that seem *burned* are small fragments (under 2 cm in length), it is hard to determine burning solely by color. An analysis with FTIR would complement this analysis and help to confirm if the pieces in question are really burned or colored differently because of other taphonomic processes.

*Carnivore* and *rodent gnawing* was examined with the help of Monrepos’ comparative taphonomy collection. The features are identified following Lyman [3].

*Root etching* is a common phenomenon in Kalavan 2’s faunal material. These traces of biochemical etching on the bone surface unfortunately cover large areas on some of the larger bone specimens. They’re rather easy to detect and are described following Lyman [3] and Binford [8].

*Trampling marks* are striations, that are made by fine, sandy, or gravely sediment being pressed and rubbed over the surface of an object, mostly through applying pressure from any direction (like animals moving over the sediment’s surface). Such marks can be confused with cut marks. They are distinguished microscopically on several features such as trajectory, shape of their groove, and inner striations [5].

Bone fracture patterns

The identification of patterns of bone breakage or fractures were carried out according to Villa and Mahieu[9] and Lyman [3]. The analysis of these patterns help to interpret whether bones were broken in a fresh or dry state. According to their work, the morphology of the breakage (e.g. spiral, rectangular, longitudinal), the broken surface (flat, rough or splintered), and thus the timing of the break (e.g. fresh, dry, recently overprinted) was recorded for every fragment larger than 2 cm. The interpretation of breakage patterns can be indicative of the influence of taphonomic agents such as animal chewing or anthropogenic markers such as the result of marrow extraction. Additionally, the morphology of the bones or fragments thereof was measured with a caliper and recorded for later fracture analysis and other examinations.

Body size

To mediate with the limited rate of identifiable material, bones were also assigned to a specific size class of animals, according to their weight. The correlation was made by the length or size of the bones or bone fragments and their cortical thickness.

These size classes are derived from several other archaeozoological analyses and tailored to this study while maintaining a comparable framework. They range from 0 to 5 and raise progressively according to weight. With 0 translating to micro mammals of a few grams to a couple of 100 g (e.g. hamsters). Class 1 is for everything that weights more than class 0 but less than 20 kg, such as hares, foxes, or badgers. Class 2 ranges from 20 to around 120 kg, for small cervids (e.g. roe deer, gazelle), goat, sheep, etc. Size class 3 comprises animals that weight between 120 to 260 kg, like medium cervids (e.g. red deer, reindeer, etc.) or onagers. From 300 kg to around a ton, size class 4 includes in this study especially larger ungulates such as large bovids and horses. Everything above would be considered Megafauna, such as *Proboscidea* or rhinos, but is absent from the present assemblages.

# Dental Microwear Texture and Mesowear Analysis methodology

Mesowear

Teeth were scored according to two schemes: (1) the original mesowear method developed by [10] and named "Mesowear I" by Ackermans [11], and (2) an expanded mesowear method following Winkler & Kaiser [12] and Taylor et al. [13], referred to as "Mesowear I & II – expanded" by Ackermans [11].

Cleaning

Prior to molding, enamel bands that seemed to be well-preserved were cleaned with acetone and cotton swabs until no sediment could be observed under a binocular microscope (Leica M420 – total optical magnification 6.3 to 32×). None of the specimens analyzed seemed to have post-mortem alterations.

Molding

Interesting enamel bands were molded with Provil novo Light regular set applied with the dispensing gun (Kulzer GmbH, Hanau, Germany). A first mold was taken as a final cleaning step and thrown away. The second mold was taken for further analysis.

Dental microwear texture analysis (DMTA)

Molds were scanned both on the Leica DCM8 at the PALEVOPRIM lab (UMR 7262 – CNRS, INEE, and University of Poitiers, France) and on the Zeiss LSM800 MAT at the TraCEr laboratory (Neuwied, Germany).

The Leica DCM8 was equipped with a Leica 100× objective (numerical aperture = 0.90 and working distance = 0.9 mm), acquiring areas of 333 × 251 µm for 2584 × 1945 pixels. The step size was set to 0.20μm and the white LED has a wavelength of 550 nm. The LSM 800 MAT was equipped with Zeiss 50× objective (numerical aperture = 0.95 and working distance = 0.22 mm), acquiring areas of 255.56 × 255.56 µm for 1981 × 1981 pixels. The step size was set to 0.20μm and the violet laser has a wavelength of 405 nm. Lateral (x and y) pixel size was therefore identical on both microscopes: 0.129 µm.

Since the sample is too small, the scans were not processed further.

# Microfauna methodology

All trenches were dry-sieved at 10 mm and any microfauna remains were collected. The trenches T1, T2, and T3 were wet-sieved with 2 mm mesh. In trench T4 two adjoining 50 cm sub-squares forming a 3-meter-deep column were systematically sampled in 5 cm spits (120 samples in total) and the weight of the sediment was recorded (2078 kg in total). Every fifth sample was sieved with 0.5 mm mesh, whilst the rest was processed with a 2 mm mesh. The sediments were dried and picked for any faunal remains. Analyses were done under binocular microscopes and taxonomic identification was done using the reference collection at MONREPOS (Archaeological Research Centre and Museum for Human Behavioural Evolution) and academic literature. Noting the composition, completeness, and preservation of cranial and postcranial elements following Andrews [14] allowed for interpretations of on-site taphonomy.

**Reference:**

1. Lyman RL. Quantitative Paleozoology. Cambridge: Cambridge University Press; 2008. doi:10.1017/CBO9780511813863

2. Reitz, E. J. WES. Zooarchaeology. 2 ed. Cambridge UK: Cambridge University Press; 2008.

3. Lyman RL. Vertebrate Taphonomy. Cambridge University Press; 1994. doi:10.1017/CBO9781139878302

4. Lyman RL. Quantitative Units and Terminology in Zooarchaeology. Vol. Am Antiq. 1994;59: 36–71.

5. Domínguez-Rodrigo, M., de Juana, S., Galán, M., Rodríguez M. A new protocol to differentiate trampling marks from butchery cut marks. J Archaeol Sci. 2009;36: 2643–2654.

6. Soulier M-C, Costamagno S. Let the cutmarks speak! Experimental butchery to reconstruct carcass processing. J Archaeol Sci Reports. 2017;11: 782–802. doi:10.1016/j.jasrep.2016.12.033

7. Domínguez-Rodrigo, M., Saladié, P., Cáceres, I., Huguet, R., Yravedra, J., Rodríguez- Hidalgo, A., Martín, P., Pineda, A., Marín, J., Gené, C., Aramendi, J., Cobo-Sánchez L. Use and abuse of cut mark analyses: the Rorschach effect. J Archaeol Sci. 2017;86: 14–23.

8. Binford LR. Bones: Ancient Men and Modern Myths. New York: Academic Press; 1981.

9. Villa, P., Mahieu E. Breakage patterns of human long bones. J Hum Evol. 1991;21: 27–48.

10. Fortelius, M., Solounias N. Functional Characterization of Ungulate Molars Using the Abrasion-Attrition Wear Gradient: A New Method for Reconstructing Paleodiets. Am Museum Novit. 2000;3301: 1–36.

11. Ackermans NL. The history of mesowear: a review. PeerJ. 2020;8: e8519.

12. Winkler, D.E., Kaiser TM. A case study of seasonal, sexual and ontogenetic divergence in the feeding behaviour of the moose (Alces alces LINNÉ, 1758). Verhandlungen des Naturwissenschaftlichen Vereins Hambg. 2011;46: 331–348.

13. Taylor LA, Müller DWH, Schwitzer C, Kaiser TM, Castell JC, Clauss M, et al. Comparative analyses of tooth wear in free-ranging and captive wild equids. Equine Vet J. 2016;48: 240–245. doi:10.1111/evj.12408

14. Andrews P. Owls, Caves, and Fossils: Predation, Preservation, and Accumulation of Small Mammal Bones in Caves, with an Analysis of the Pleistocene Cave Faunas from Westbury-sub-Mendip, Somerset, UK. Chicago: University of Chicago Press; 1990.
