# Supplementary figures and images for: Short-term occupations at high elevation during the Middle Paleolithic at Kalavan 2 (Republic of Armenia)
Source: PLoS One. 2021 Feb 4;16(2):e0245700. doi: 10.1371/journal.pone.0245700 (PMC7861461; doi:10.1371/journal.pone.0245700)

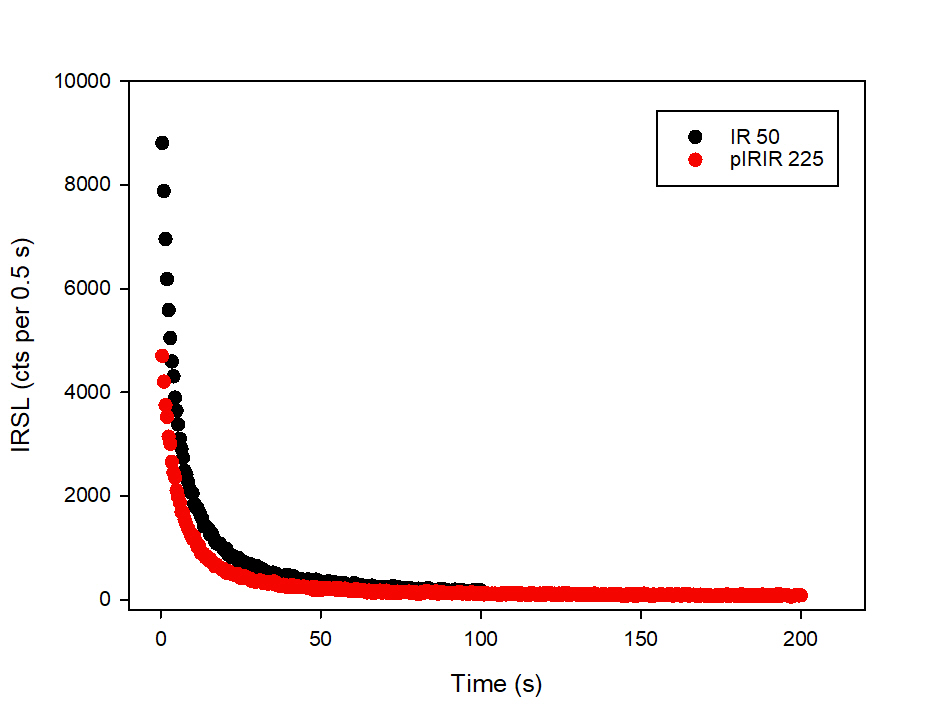

Supplement: S1 Fig — 1: IR50 and pIRIR225 decay curves of the natural luminescence signal obtained from fine-grain polyminerals (sample L-Eva 1681). 2: IR50 and pIRIR225 decay curves of the natural luminescence signal obtained from coarse grain K-feldspars (sample L-Eva 1681). 3: Results of dose recovery tests (pIRIR225 signal). All measured- to given dose ratios deviate < 10% from unity. 4: IR50- and pIRIR225 related g-values obtained on samples L-Eva 1684, 1685 and 1686. 5: Chemical classification diagrams of the glass analytical data obtained from the Kalavan tephras. 6: Glass shard concentrations vs. depth at Kalavan 2 Trench 1 & 2. Grey bars denote 10 cm scan samples and orange bars represent refined 2 cm intervals. 7: Selected chemical bi-plots of non-normalised glass compositional data from visible tephra identified at Kalavan 2. Comparisons are made to the Çekmece Formation derived from Nemrut in the EAVP. Comparative data from Macdonald et al. (2015). (ZIP) [file pone.0245700.s005.zip › Sp 1 Fig. 1 pIRIR225-4-11-poly-nat-signal.tif]

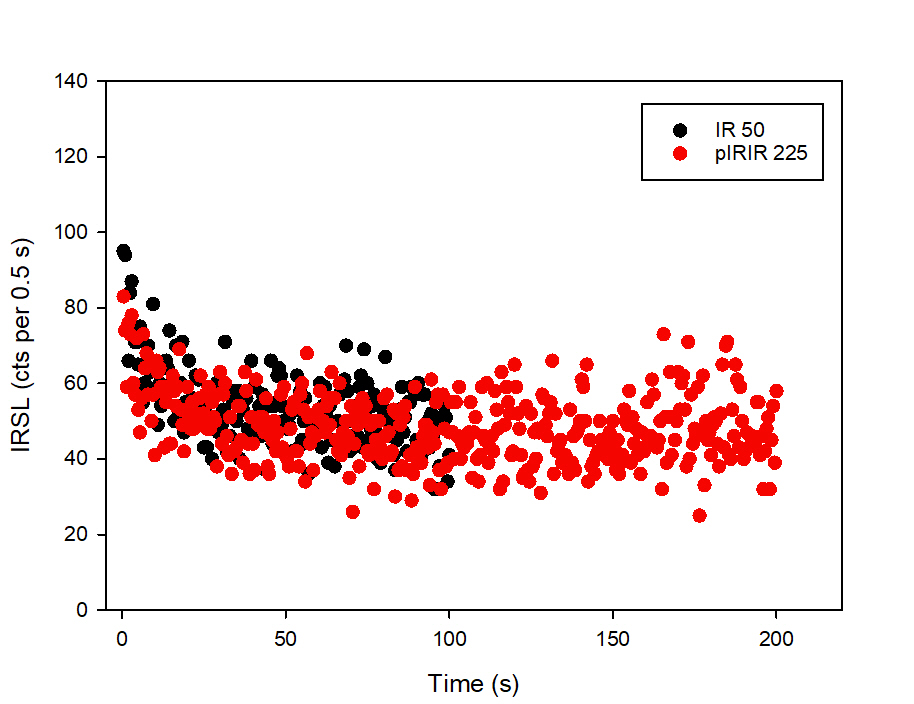

Supplement: S1 Fig — 1: IR50 and pIRIR225 decay curves of the natural luminescence signal obtained from fine-grain polyminerals (sample L-Eva 1681). 2: IR50 and pIRIR225 decay curves of the natural luminescence signal obtained from coarse grain K-feldspars (sample L-Eva 1681). 3: Results of dose recovery tests (pIRIR225 signal). All measured- to given dose ratios deviate < 10% from unity. 4: IR50- and pIRIR225 related g-values obtained on samples L-Eva 1684, 1685 and 1686. 5: Chemical classification diagrams of the glass analytical data obtained from the Kalavan tephras. 6: Glass shard concentrations vs. depth at Kalavan 2 Trench 1 & 2. Grey bars denote 10 cm scan samples and orange bars represent refined 2 cm intervals. 7: Selected chemical bi-plots of non-normalised glass compositional data from visible tephra identified at Kalavan 2. Comparisons are made to the Çekmece Formation derived from Nemrut in the EAVP. Comparative data from Macdonald et al. (2015). (ZIP) [file pone.0245700.s005.zip › Sp 1 Fig. 2 pIRIR225-0.5mm-180-250-nat-signal.tif]

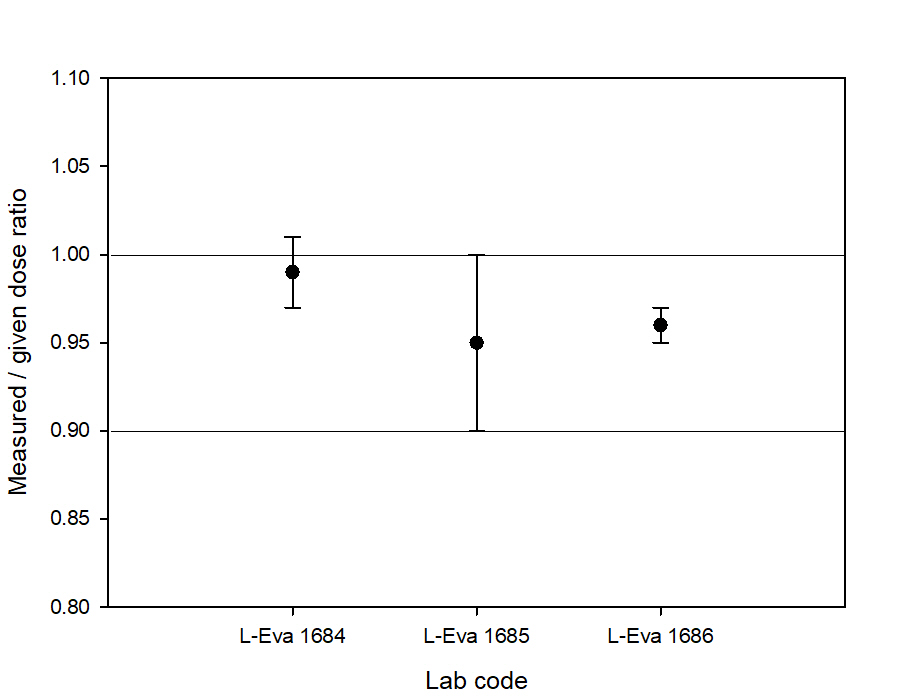

Supplement: S1 Fig — 1: IR50 and pIRIR225 decay curves of the natural luminescence signal obtained from fine-grain polyminerals (sample L-Eva 1681). 2: IR50 and pIRIR225 decay curves of the natural luminescence signal obtained from coarse grain K-feldspars (sample L-Eva 1681). 3: Results of dose recovery tests (pIRIR225 signal). All measured- to given dose ratios deviate < 10% from unity. 4: IR50- and pIRIR225 related g-values obtained on samples L-Eva 1684, 1685 and 1686. 5: Chemical classification diagrams of the glass analytical data obtained from the Kalavan tephras. 6: Glass shard concentrations vs. depth at Kalavan 2 Trench 1 & 2. Grey bars denote 10 cm scan samples and orange bars represent refined 2 cm intervals. 7: Selected chemical bi-plots of non-normalised glass compositional data from visible tephra identified at Kalavan 2. Comparisons are made to the Çekmece Formation derived from Nemrut in the EAVP. Comparative data from Macdonald et al. (2015). (ZIP) [file pone.0245700.s005.zip › Sp 1 Fig. 3 pIRIR225-1684-1685-1686.tif]

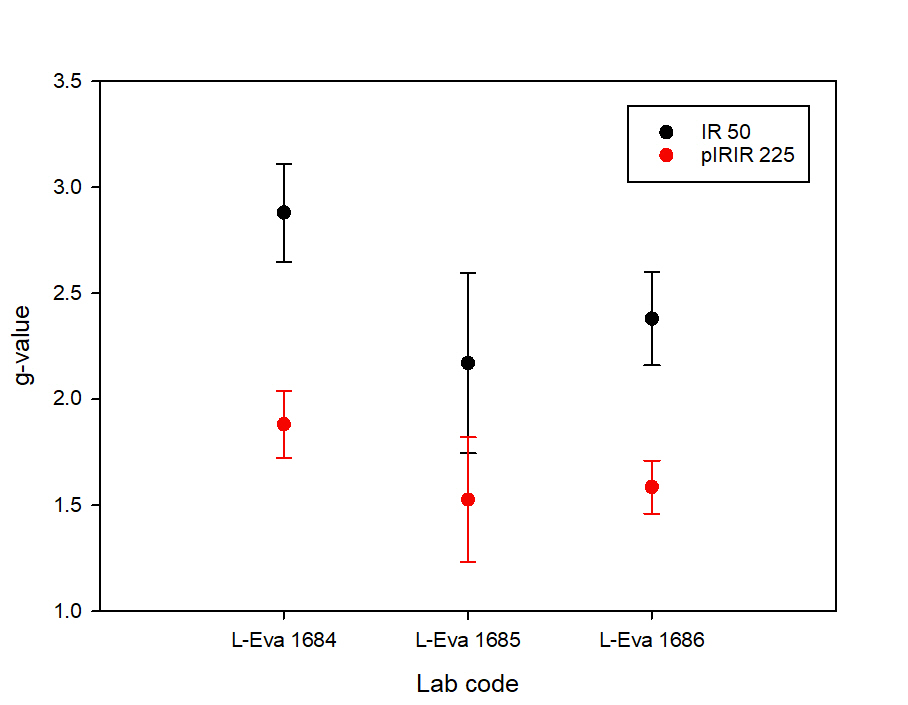

Supplement: S1 Fig — 1: IR50 and pIRIR225 decay curves of the natural luminescence signal obtained from fine-grain polyminerals (sample L-Eva 1681). 2: IR50 and pIRIR225 decay curves of the natural luminescence signal obtained from coarse grain K-feldspars (sample L-Eva 1681). 3: Results of dose recovery tests (pIRIR225 signal). All measured- to given dose ratios deviate < 10% from unity. 4: IR50- and pIRIR225 related g-values obtained on samples L-Eva 1684, 1685 and 1686. 5: Chemical classification diagrams of the glass analytical data obtained from the Kalavan tephras. 6: Glass shard concentrations vs. depth at Kalavan 2 Trench 1 & 2. Grey bars denote 10 cm scan samples and orange bars represent refined 2 cm intervals. 7: Selected chemical bi-plots of non-normalised glass compositional data from visible tephra identified at Kalavan 2. Comparisons are made to the Çekmece Formation derived from Nemrut in the EAVP. Comparative data from Macdonald et al. (2015). (ZIP) [file pone.0245700.s005.zip › Sp 1 Fig. 4 fading-pIRIR225_finegrain-1684-1685-1686.tif]

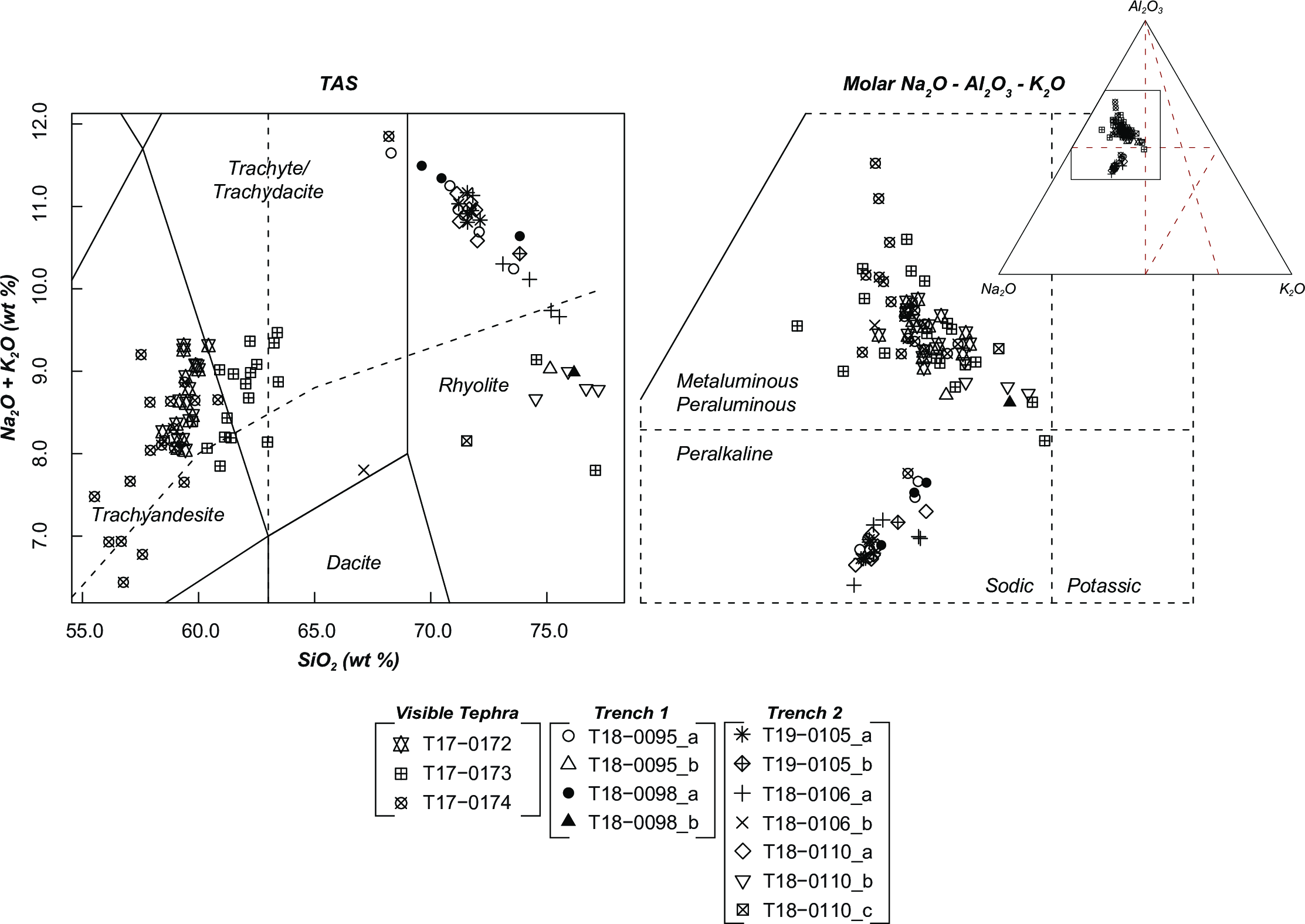

Supplement: S1 Fig — 1: IR50 and pIRIR225 decay curves of the natural luminescence signal obtained from fine-grain polyminerals (sample L-Eva 1681). 2: IR50 and pIRIR225 decay curves of the natural luminescence signal obtained from coarse grain K-feldspars (sample L-Eva 1681). 3: Results of dose recovery tests (pIRIR225 signal). All measured- to given dose ratios deviate < 10% from unity. 4: IR50- and pIRIR225 related g-values obtained on samples L-Eva 1684, 1685 and 1686. 5: Chemical classification diagrams of the glass analytical data obtained from the Kalavan tephras. 6: Glass shard concentrations vs. depth at Kalavan 2 Trench 1 & 2. Grey bars denote 10 cm scan samples and orange bars represent refined 2 cm intervals. 7: Selected chemical bi-plots of non-normalised glass compositional data from visible tephra identified at Kalavan 2. Comparisons are made to the Çekmece Formation derived from Nemrut in the EAVP. Comparative data from Macdonald et al. (2015). (ZIP) [file pone.0245700.s005.zip › Sp 1 fig. 5_Kalavan tephra classification.tif]

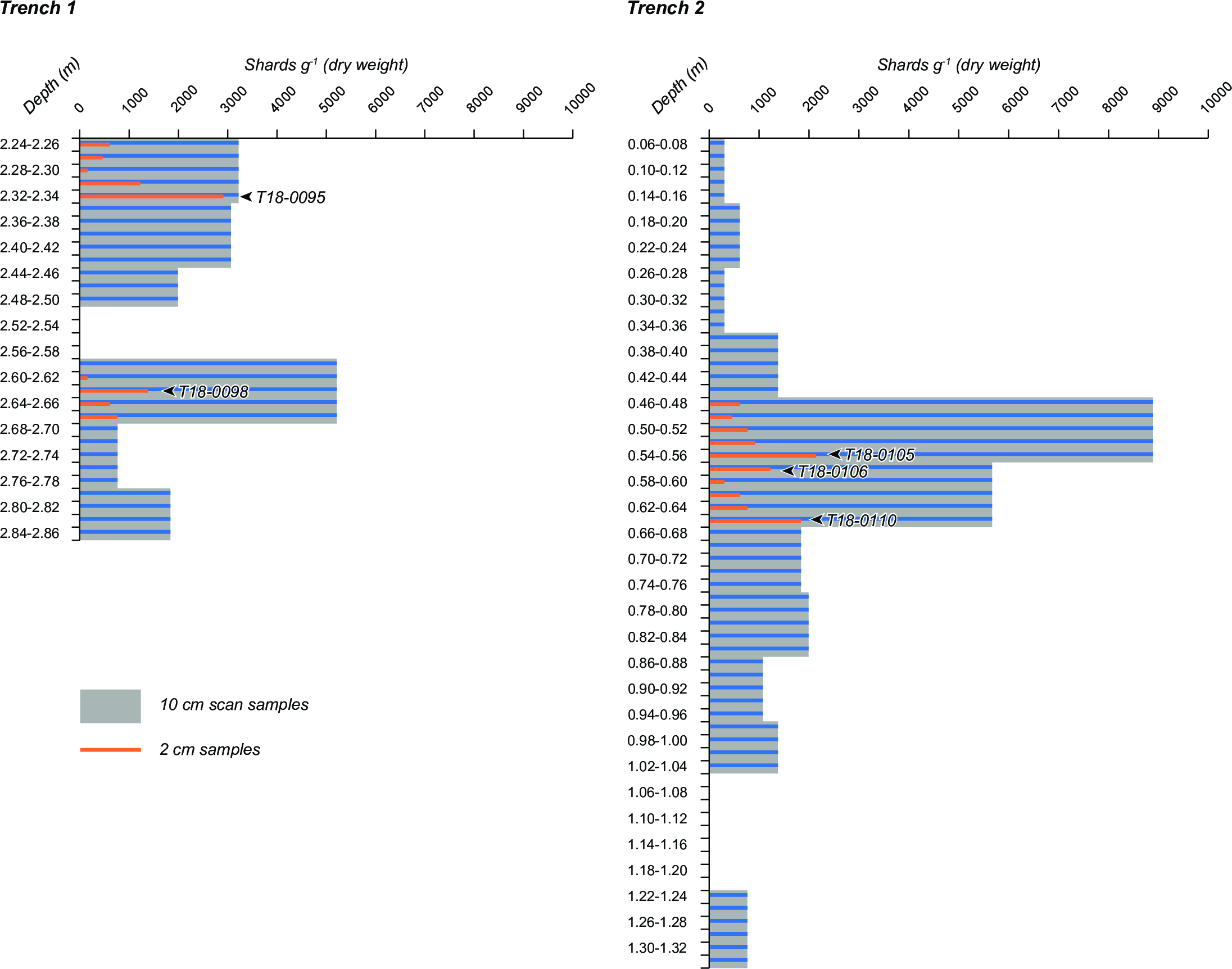

Supplement: S1 Fig — 1: IR50 and pIRIR225 decay curves of the natural luminescence signal obtained from fine-grain polyminerals (sample L-Eva 1681). 2: IR50 and pIRIR225 decay curves of the natural luminescence signal obtained from coarse grain K-feldspars (sample L-Eva 1681). 3: Results of dose recovery tests (pIRIR225 signal). All measured- to given dose ratios deviate < 10% from unity. 4: IR50- and pIRIR225 related g-values obtained on samples L-Eva 1684, 1685 and 1686. 5: Chemical classification diagrams of the glass analytical data obtained from the Kalavan tephras. 6: Glass shard concentrations vs. depth at Kalavan 2 Trench 1 & 2. Grey bars denote 10 cm scan samples and orange bars represent refined 2 cm intervals. 7: Selected chemical bi-plots of non-normalised glass compositional data from visible tephra identified at Kalavan 2. Comparisons are made to the Çekmece Formation derived from Nemrut in the EAVP. Comparative data from Macdonald et al. (2015). (ZIP) [file pone.0245700.s005.zip › Sp 1 fig. 6 Kalavan2_cryptotephrostratigraphy_trench 1 & 2.tif]

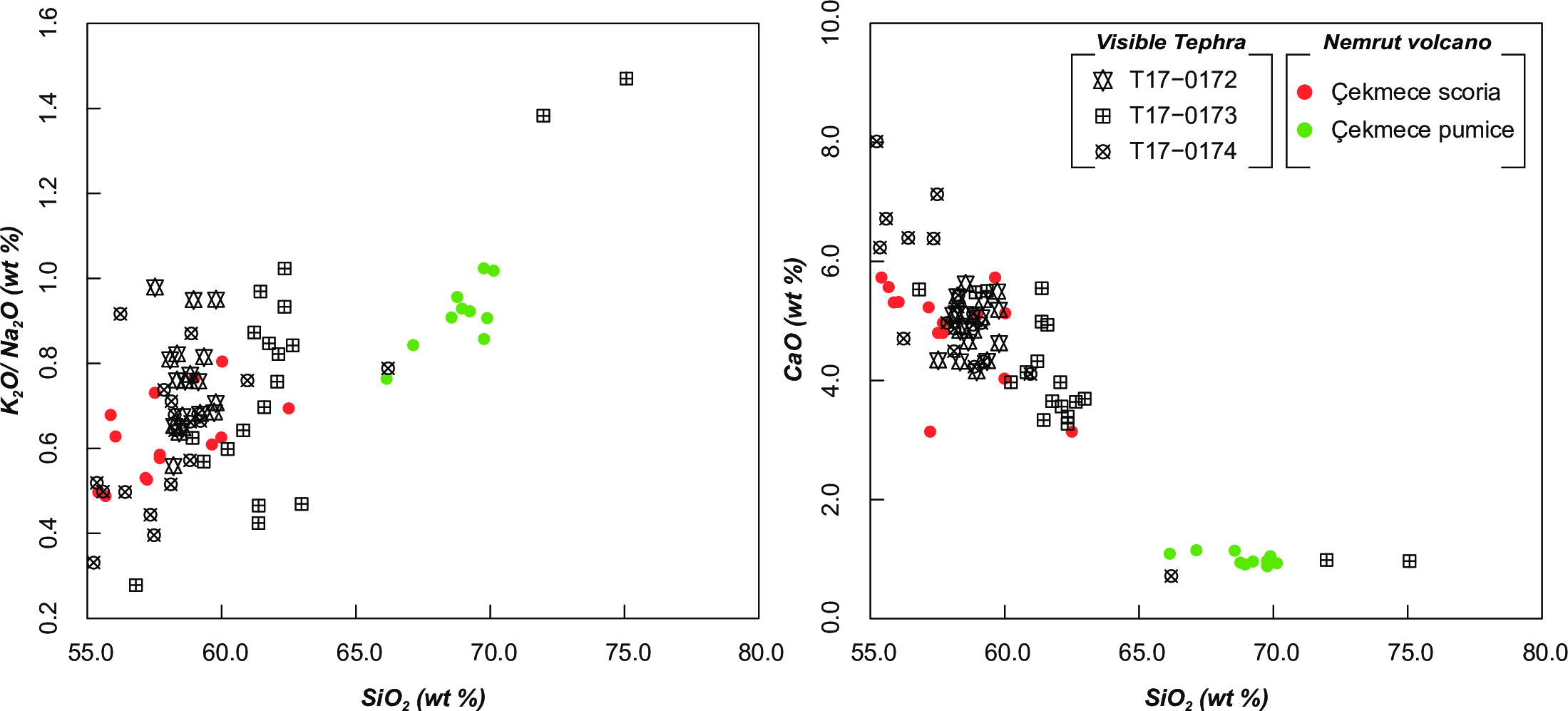

Supplement: S1 Fig — 1: IR50 and pIRIR225 decay curves of the natural luminescence signal obtained from fine-grain polyminerals (sample L-Eva 1681). 2: IR50 and pIRIR225 decay curves of the natural luminescence signal obtained from coarse grain K-feldspars (sample L-Eva 1681). 3: Results of dose recovery tests (pIRIR225 signal). All measured- to given dose ratios deviate < 10% from unity. 4: IR50- and pIRIR225 related g-values obtained on samples L-Eva 1684, 1685 and 1686. 5: Chemical classification diagrams of the glass analytical data obtained from the Kalavan tephras. 6: Glass shard concentrations vs. depth at Kalavan 2 Trench 1 & 2. Grey bars denote 10 cm scan samples and orange bars represent refined 2 cm intervals. 7: Selected chemical bi-plots of non-normalised glass compositional data from visible tephra identified at Kalavan 2. Comparisons are made to the Çekmece Formation derived from Nemrut in the EAVP. Comparative data from Macdonald et al. (2015). (ZIP) [file pone.0245700.s005.zip › Sp 1 fig. 7 visible tephra correlations.tif]

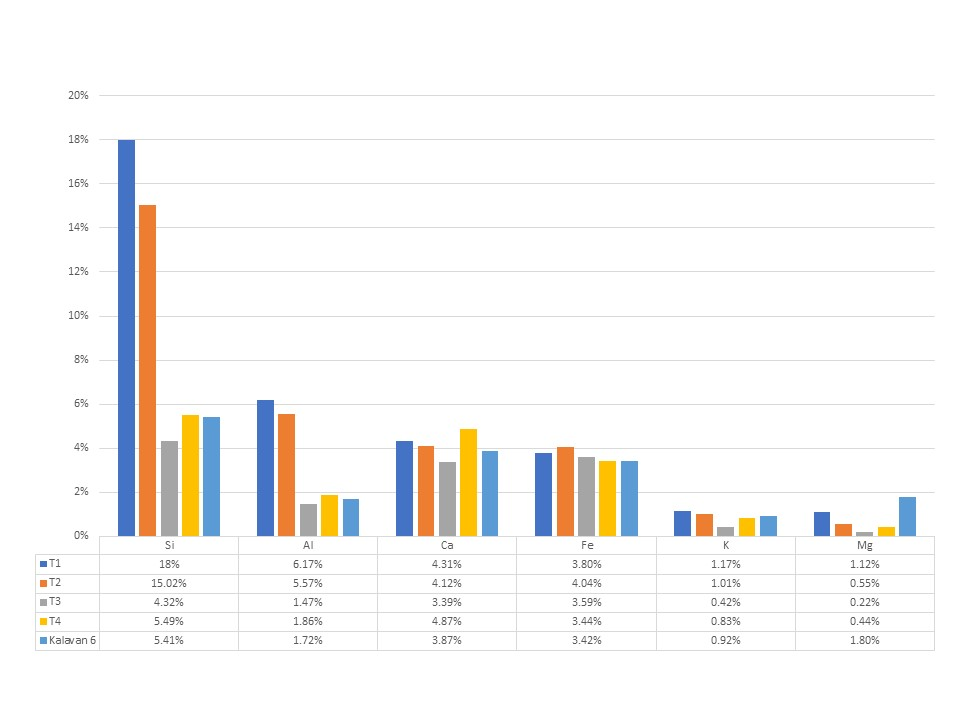

Supplement: S2 Fig — 1: pXRF results of elemental by trench. 2–9: Micromorphological samples pictures with details on the main micromorphological features: Ob = Obsidian flake; Bo = Bone; Co = Compaction traces; Vo = Void; Mn = Manganese; Bi = Bioturbation; Fm = Frost microstructure; Ve = Veins of Magnesium, gypsum and/or calcite; Rs = Rotation structure. 10: Odd-over-even predominance (OEP) and average chain length (ACL) of n-alkanes from Kalavan-2 (red triangles) compared to modern grass soils (yellow squares) and deciduous soils (green squares). Modern data from Bleidtner et al 2018. 11: n-alkane abundances of samples from the Kalavan-2 ~55kya sedimentary unit (red triangles) compared to modern grass soils (yellow squares) and deciduous soils (green squares). Modern data from Bleidtner et al 2018. (ZIP) [file pone.0245700.s006.zip › Sp 2 fig. 1. pXRF results of elemental by trench.tif]

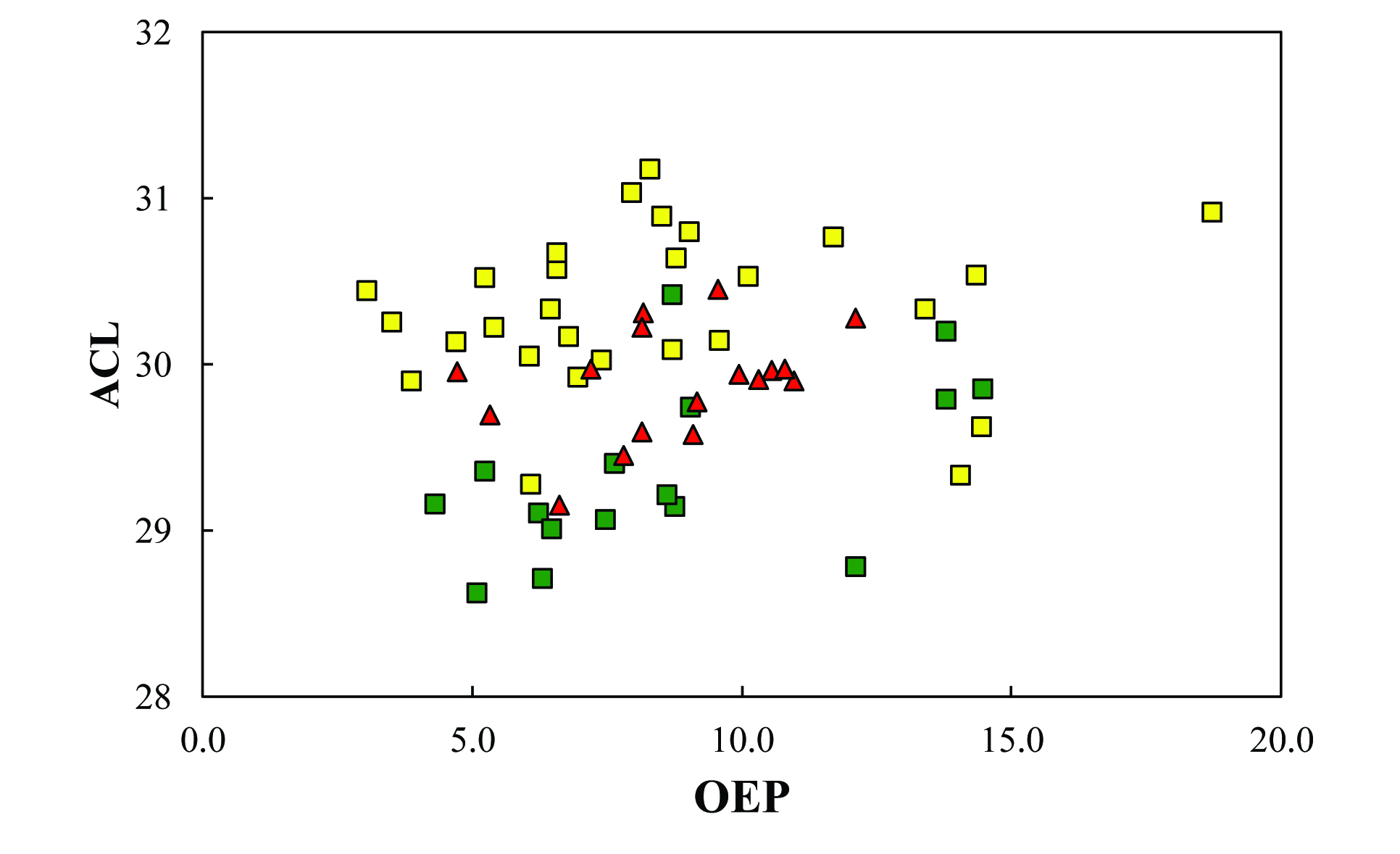

Supplement: S2 Fig — 1: pXRF results of elemental by trench. 2–9: Micromorphological samples pictures with details on the main micromorphological features: Ob = Obsidian flake; Bo = Bone; Co = Compaction traces; Vo = Void; Mn = Manganese; Bi = Bioturbation; Fm = Frost microstructure; Ve = Veins of Magnesium, gypsum and/or calcite; Rs = Rotation structure. 10: Odd-over-even predominance (OEP) and average chain length (ACL) of n-alkanes from Kalavan-2 (red triangles) compared to modern grass soils (yellow squares) and deciduous soils (green squares). Modern data from Bleidtner et al 2018. 11: n-alkane abundances of samples from the Kalavan-2 ~55kya sedimentary unit (red triangles) compared to modern grass soils (yellow squares) and deciduous soils (green squares). Modern data from Bleidtner et al 2018. (ZIP) [file pone.0245700.s006.zip › Sp 2 fig. 10 ACL vs OEP-1.tif]

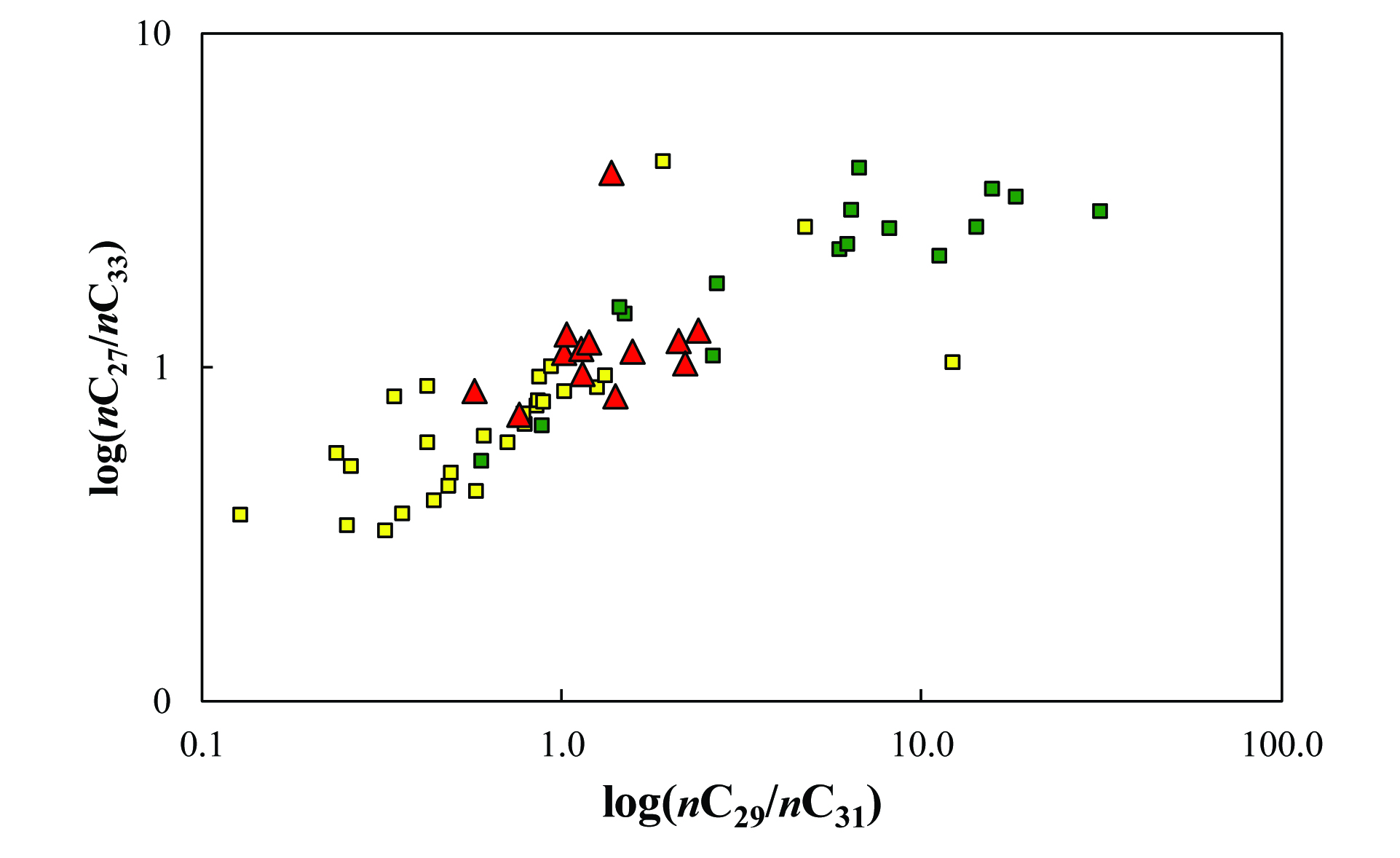

Supplement: S2 Fig — 1: pXRF results of elemental by trench. 2–9: Micromorphological samples pictures with details on the main micromorphological features: Ob = Obsidian flake; Bo = Bone; Co = Compaction traces; Vo = Void; Mn = Manganese; Bi = Bioturbation; Fm = Frost microstructure; Ve = Veins of Magnesium, gypsum and/or calcite; Rs = Rotation structure. 10: Odd-over-even predominance (OEP) and average chain length (ACL) of n-alkanes from Kalavan-2 (red triangles) compared to modern grass soils (yellow squares) and deciduous soils (green squares). Modern data from Bleidtner et al 2018. 11: n-alkane abundances of samples from the Kalavan-2 ~55kya sedimentary unit (red triangles) compared to modern grass soils (yellow squares) and deciduous soils (green squares). Modern data from Bleidtner et al 2018. (ZIP) [file pone.0245700.s006.zip › Sp 2 fig. 11 Kalavan 2 55 kya samples-2.tif]

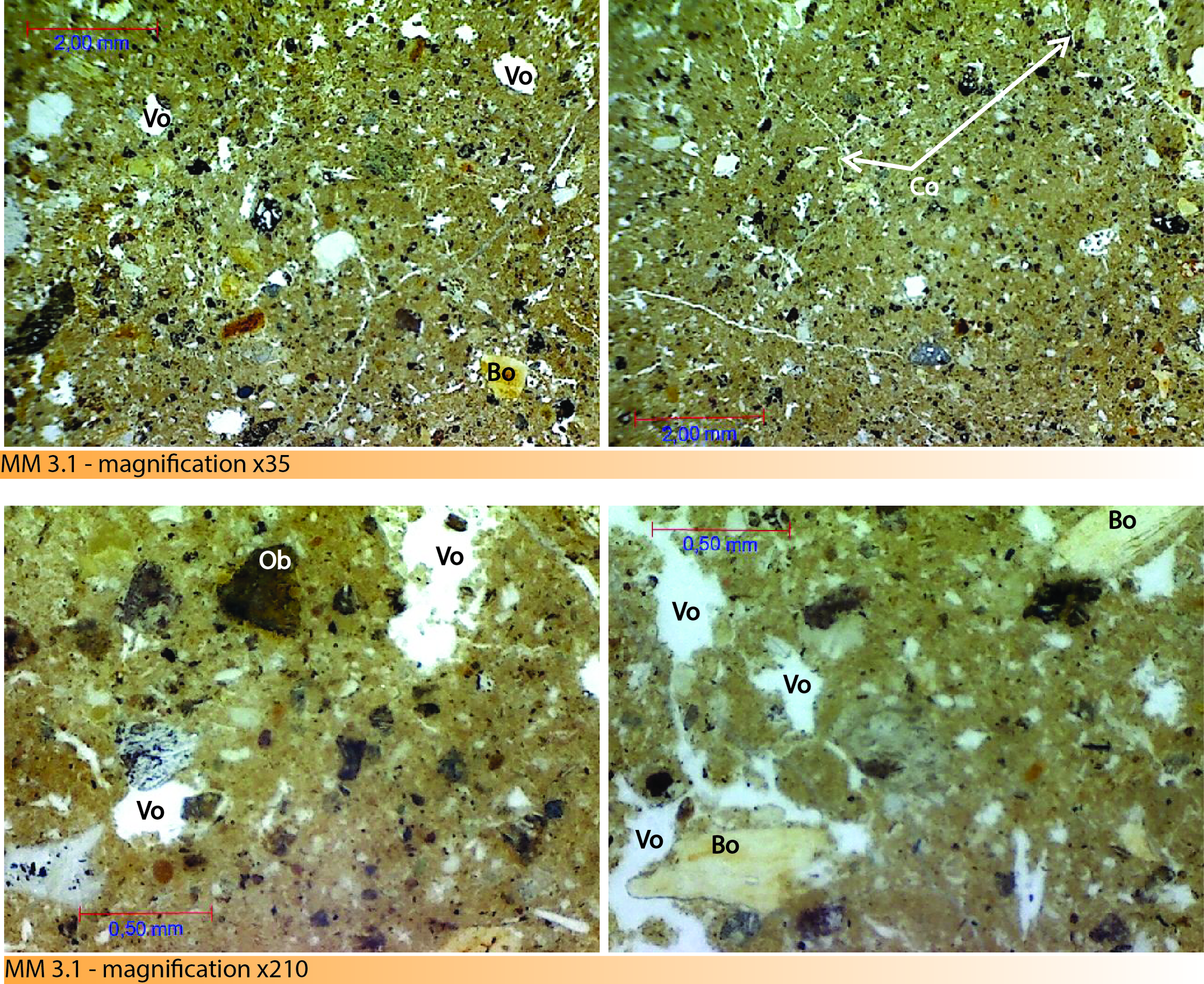

Supplement: S2 Fig — 1: pXRF results of elemental by trench. 2–9: Micromorphological samples pictures with details on the main micromorphological features: Ob = Obsidian flake; Bo = Bone; Co = Compaction traces; Vo = Void; Mn = Manganese; Bi = Bioturbation; Fm = Frost microstructure; Ve = Veins of Magnesium, gypsum and/or calcite; Rs = Rotation structure. 10: Odd-over-even predominance (OEP) and average chain length (ACL) of n-alkanes from Kalavan-2 (red triangles) compared to modern grass soils (yellow squares) and deciduous soils (green squares). Modern data from Bleidtner et al 2018. 11: n-alkane abundances of samples from the Kalavan-2 ~55kya sedimentary unit (red triangles) compared to modern grass soils (yellow squares) and deciduous soils (green squares). Modern data from Bleidtner et al 2018. (ZIP) [file pone.0245700.s006.zip › Sp 2 fig. 2 Kalavan MM 3.1 figure.tif]

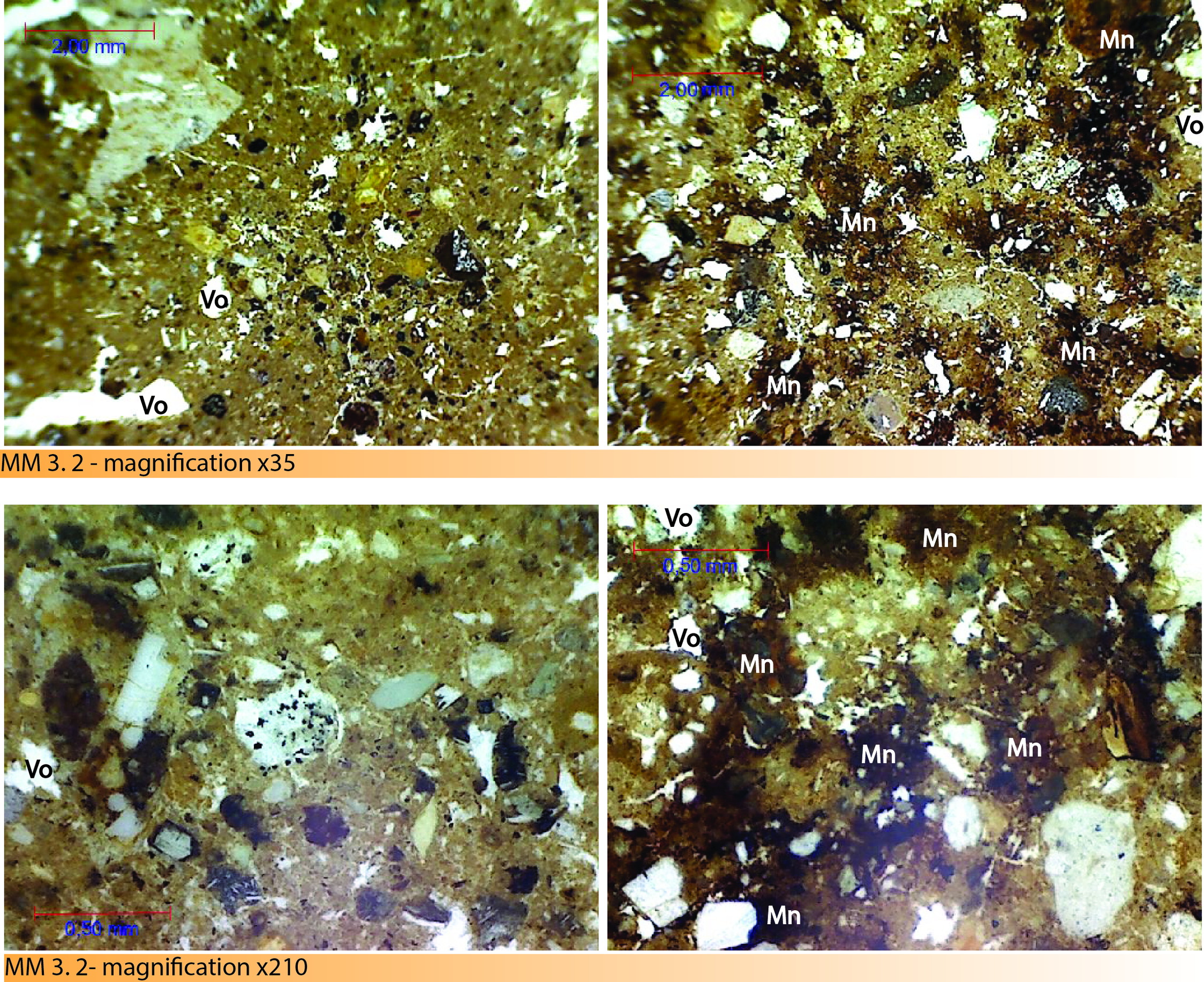

Supplement: S2 Fig — 1: pXRF results of elemental by trench. 2–9: Micromorphological samples pictures with details on the main micromorphological features: Ob = Obsidian flake; Bo = Bone; Co = Compaction traces; Vo = Void; Mn = Manganese; Bi = Bioturbation; Fm = Frost microstructure; Ve = Veins of Magnesium, gypsum and/or calcite; Rs = Rotation structure. 10: Odd-over-even predominance (OEP) and average chain length (ACL) of n-alkanes from Kalavan-2 (red triangles) compared to modern grass soils (yellow squares) and deciduous soils (green squares). Modern data from Bleidtner et al 2018. 11: n-alkane abundances of samples from the Kalavan-2 ~55kya sedimentary unit (red triangles) compared to modern grass soils (yellow squares) and deciduous soils (green squares). Modern data from Bleidtner et al 2018. (ZIP) [file pone.0245700.s006.zip › Sp 2 fig. 3 Kalavan MM 3.2 figure.tif]

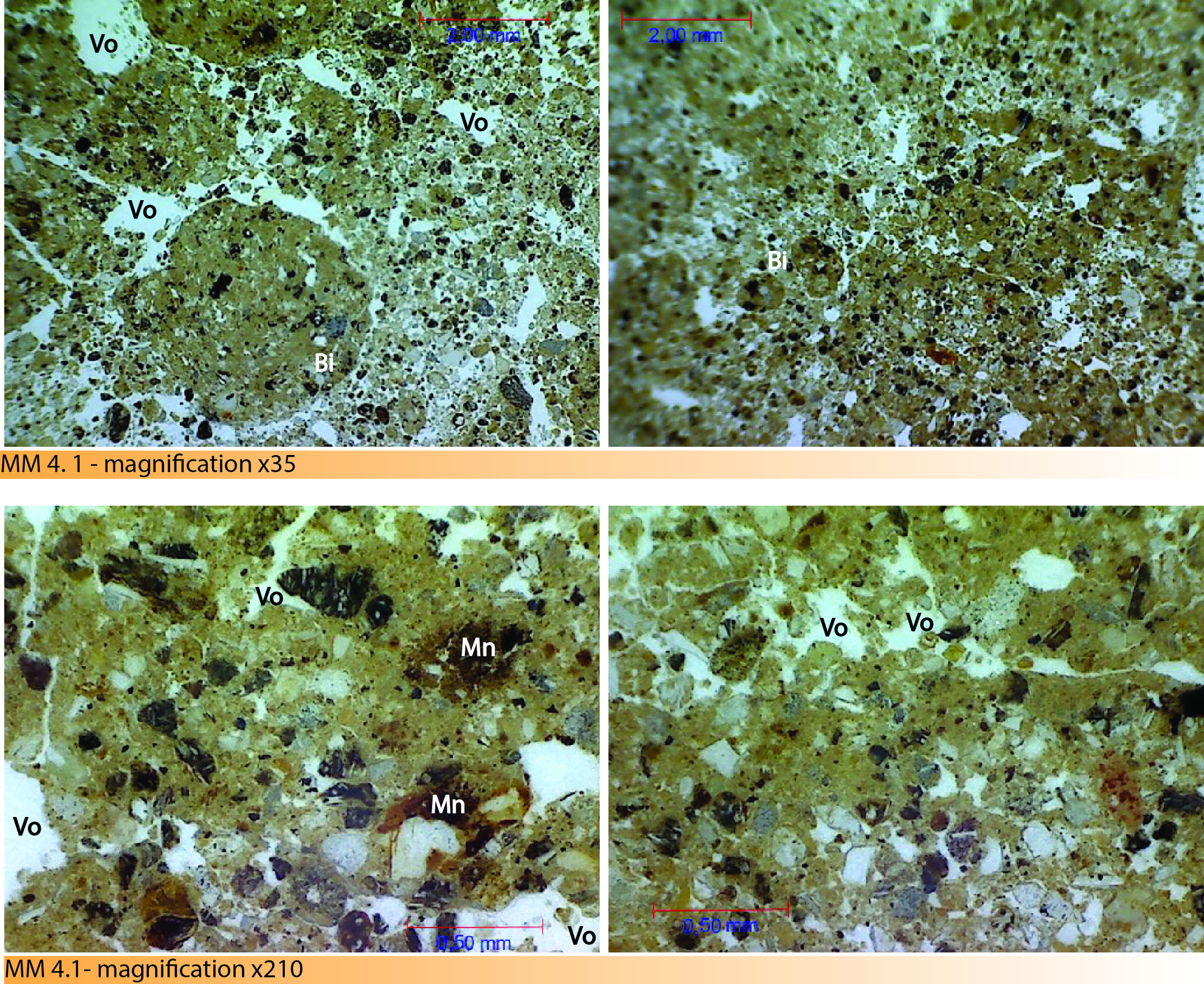

Supplement: S2 Fig — 1: pXRF results of elemental by trench. 2–9: Micromorphological samples pictures with details on the main micromorphological features: Ob = Obsidian flake; Bo = Bone; Co = Compaction traces; Vo = Void; Mn = Manganese; Bi = Bioturbation; Fm = Frost microstructure; Ve = Veins of Magnesium, gypsum and/or calcite; Rs = Rotation structure. 10: Odd-over-even predominance (OEP) and average chain length (ACL) of n-alkanes from Kalavan-2 (red triangles) compared to modern grass soils (yellow squares) and deciduous soils (green squares). Modern data from Bleidtner et al 2018. 11: n-alkane abundances of samples from the Kalavan-2 ~55kya sedimentary unit (red triangles) compared to modern grass soils (yellow squares) and deciduous soils (green squares). Modern data from Bleidtner et al 2018. (ZIP) [file pone.0245700.s006.zip › Sp 2 fig. 4 Kalavan MM 4.1 figure.tif]

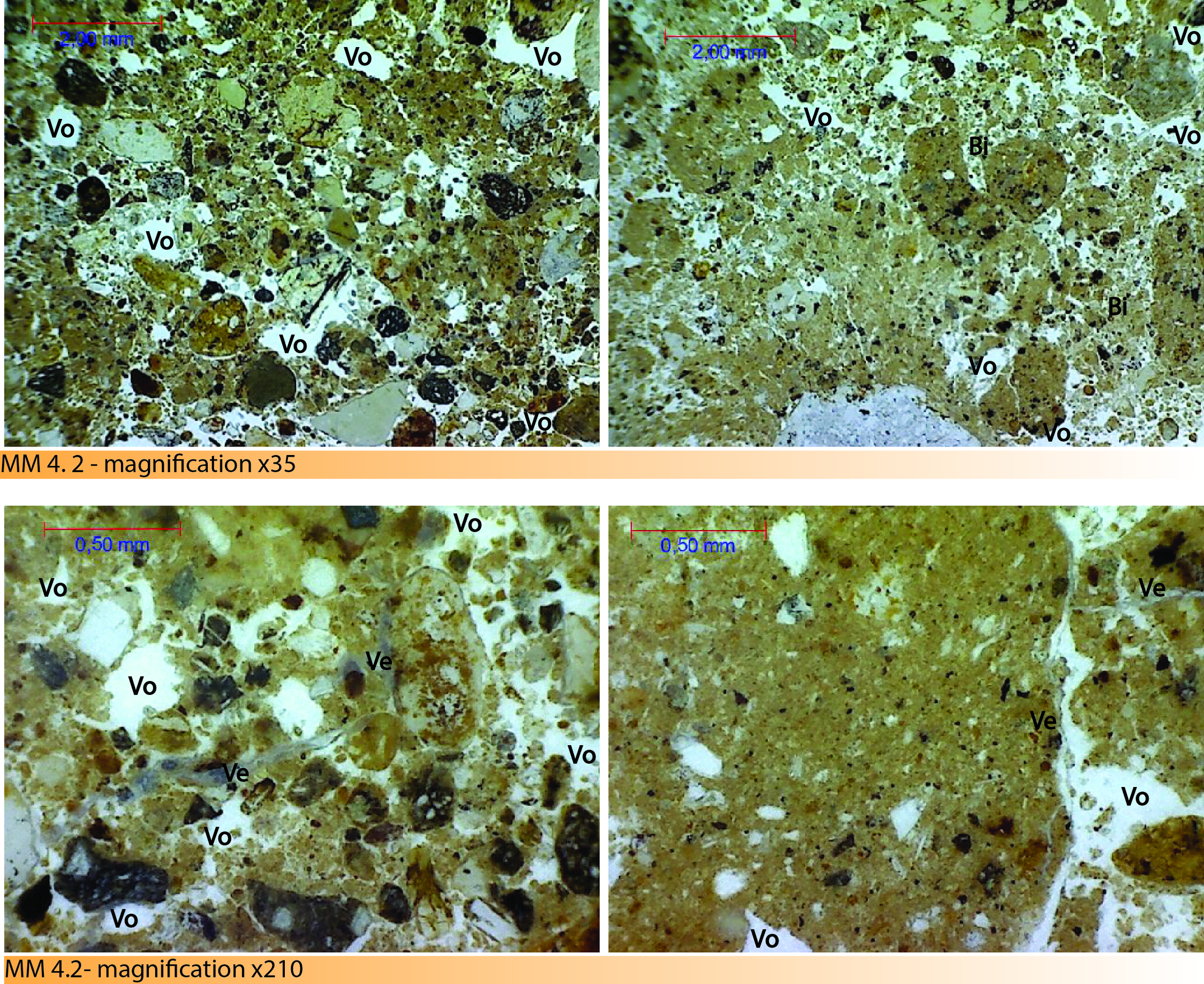

Supplement: S2 Fig — 1: pXRF results of elemental by trench. 2–9: Micromorphological samples pictures with details on the main micromorphological features: Ob = Obsidian flake; Bo = Bone; Co = Compaction traces; Vo = Void; Mn = Manganese; Bi = Bioturbation; Fm = Frost microstructure; Ve = Veins of Magnesium, gypsum and/or calcite; Rs = Rotation structure. 10: Odd-over-even predominance (OEP) and average chain length (ACL) of n-alkanes from Kalavan-2 (red triangles) compared to modern grass soils (yellow squares) and deciduous soils (green squares). Modern data from Bleidtner et al 2018. 11: n-alkane abundances of samples from the Kalavan-2 ~55kya sedimentary unit (red triangles) compared to modern grass soils (yellow squares) and deciduous soils (green squares). Modern data from Bleidtner et al 2018. (ZIP) [file pone.0245700.s006.zip › Sp 2 Fig. 5 Kalavan MM 4.2 figure.tif]

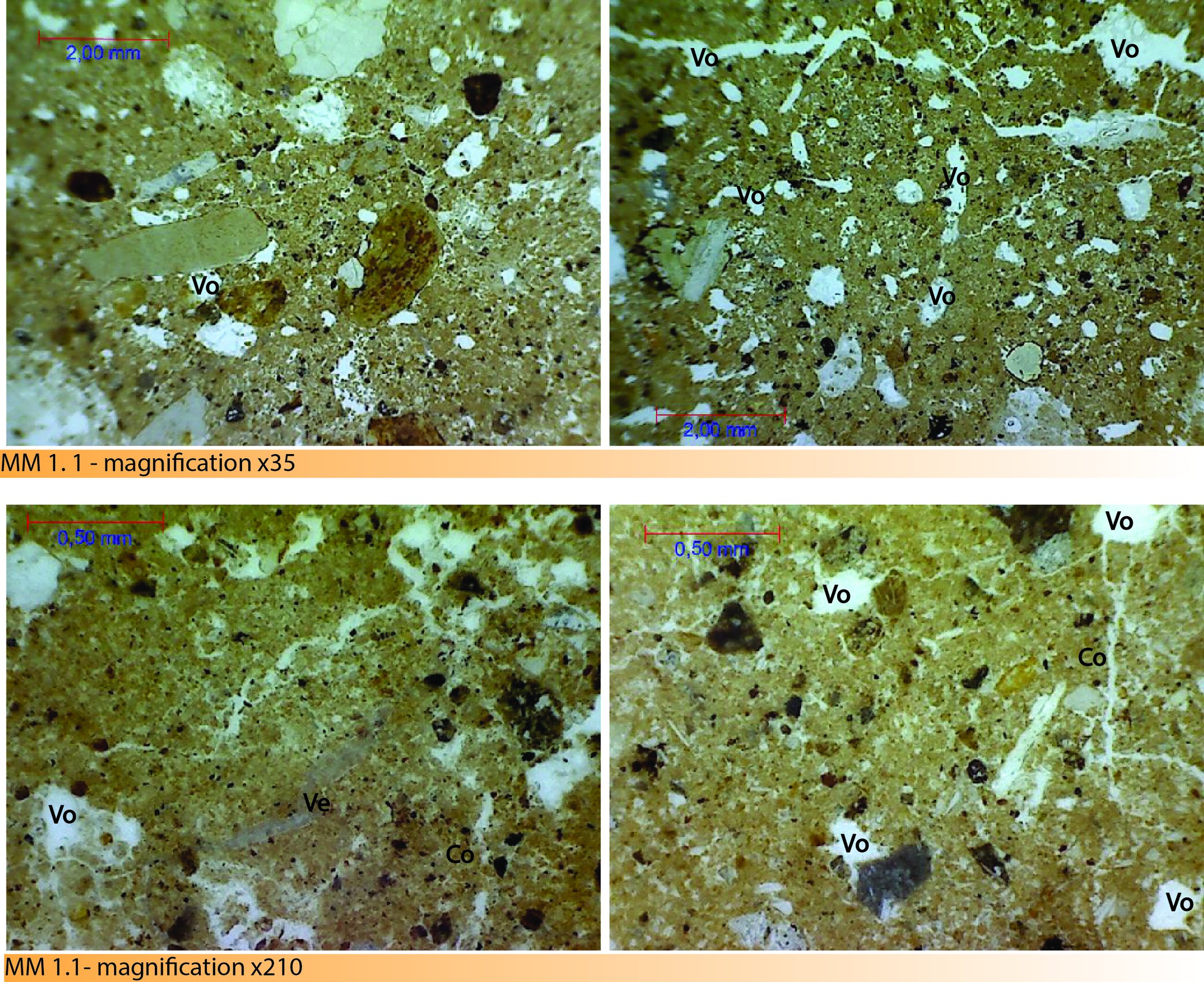

Supplement: S2 Fig — 1: pXRF results of elemental by trench. 2–9: Micromorphological samples pictures with details on the main micromorphological features: Ob = Obsidian flake; Bo = Bone; Co = Compaction traces; Vo = Void; Mn = Manganese; Bi = Bioturbation; Fm = Frost microstructure; Ve = Veins of Magnesium, gypsum and/or calcite; Rs = Rotation structure. 10: Odd-over-even predominance (OEP) and average chain length (ACL) of n-alkanes from Kalavan-2 (red triangles) compared to modern grass soils (yellow squares) and deciduous soils (green squares). Modern data from Bleidtner et al 2018. 11: n-alkane abundances of samples from the Kalavan-2 ~55kya sedimentary unit (red triangles) compared to modern grass soils (yellow squares) and deciduous soils (green squares). Modern data from Bleidtner et al 2018. (ZIP) [file pone.0245700.s006.zip › Sp 2 fig. 6 Kalavan MM 1.1 figure.tif]

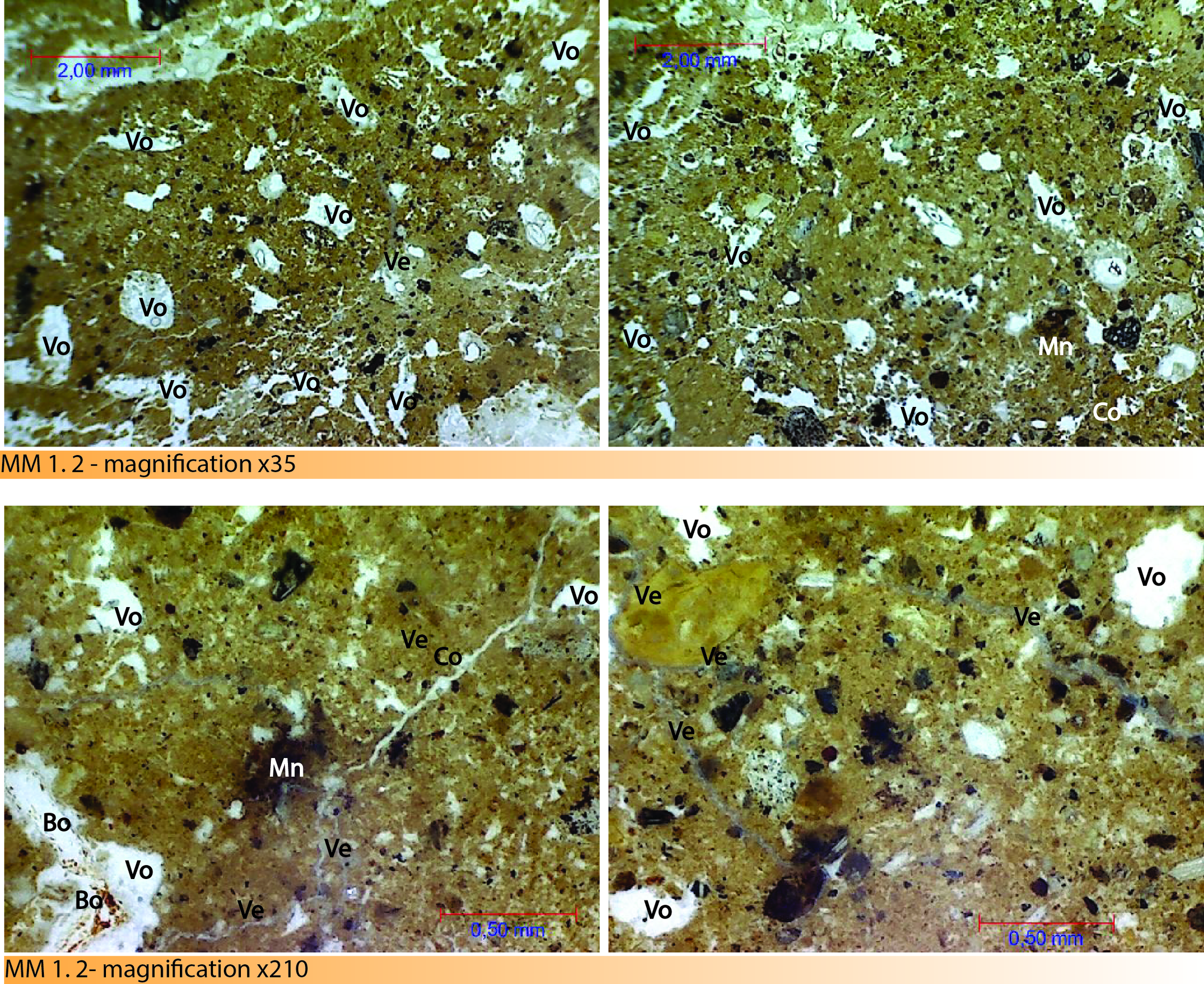

Supplement: S2 Fig — 1: pXRF results of elemental by trench. 2–9: Micromorphological samples pictures with details on the main micromorphological features: Ob = Obsidian flake; Bo = Bone; Co = Compaction traces; Vo = Void; Mn = Manganese; Bi = Bioturbation; Fm = Frost microstructure; Ve = Veins of Magnesium, gypsum and/or calcite; Rs = Rotation structure. 10: Odd-over-even predominance (OEP) and average chain length (ACL) of n-alkanes from Kalavan-2 (red triangles) compared to modern grass soils (yellow squares) and deciduous soils (green squares). Modern data from Bleidtner et al 2018. 11: n-alkane abundances of samples from the Kalavan-2 ~55kya sedimentary unit (red triangles) compared to modern grass soils (yellow squares) and deciduous soils (green squares). Modern data from Bleidtner et al 2018. (ZIP) [file pone.0245700.s006.zip › Sp 2 fig. 7 Kalavan MM 1.2 figure.tif]

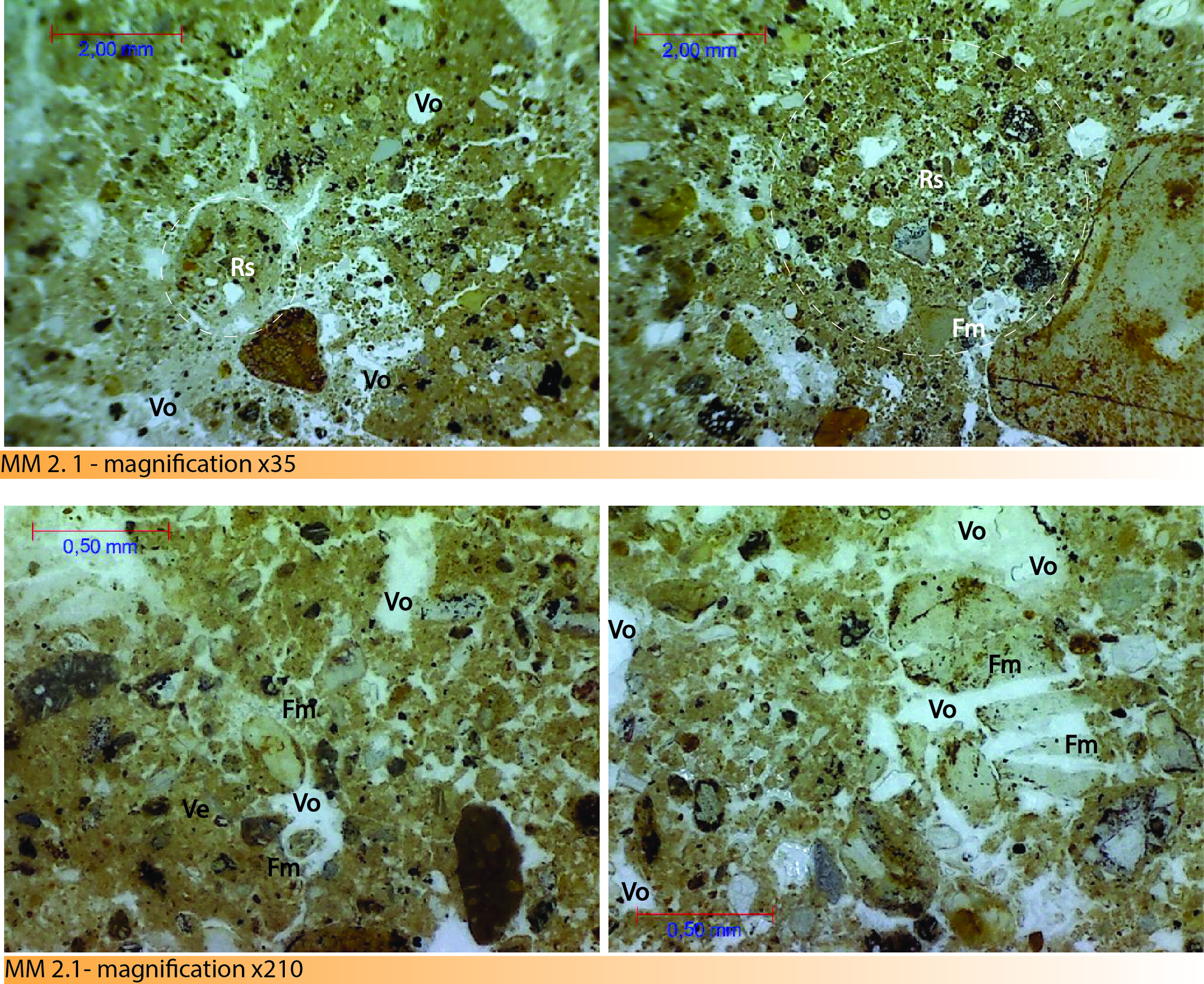

Supplement: S2 Fig — 1: pXRF results of elemental by trench. 2–9: Micromorphological samples pictures with details on the main micromorphological features: Ob = Obsidian flake; Bo = Bone; Co = Compaction traces; Vo = Void; Mn = Manganese; Bi = Bioturbation; Fm = Frost microstructure; Ve = Veins of Magnesium, gypsum and/or calcite; Rs = Rotation structure. 10: Odd-over-even predominance (OEP) and average chain length (ACL) of n-alkanes from Kalavan-2 (red triangles) compared to modern grass soils (yellow squares) and deciduous soils (green squares). Modern data from Bleidtner et al 2018. 11: n-alkane abundances of samples from the Kalavan-2 ~55kya sedimentary unit (red triangles) compared to modern grass soils (yellow squares) and deciduous soils (green squares). Modern data from Bleidtner et al 2018. (ZIP) [file pone.0245700.s006.zip › Sp 2 Fig. 8 Kalavan MM 2.1 figure.tif]

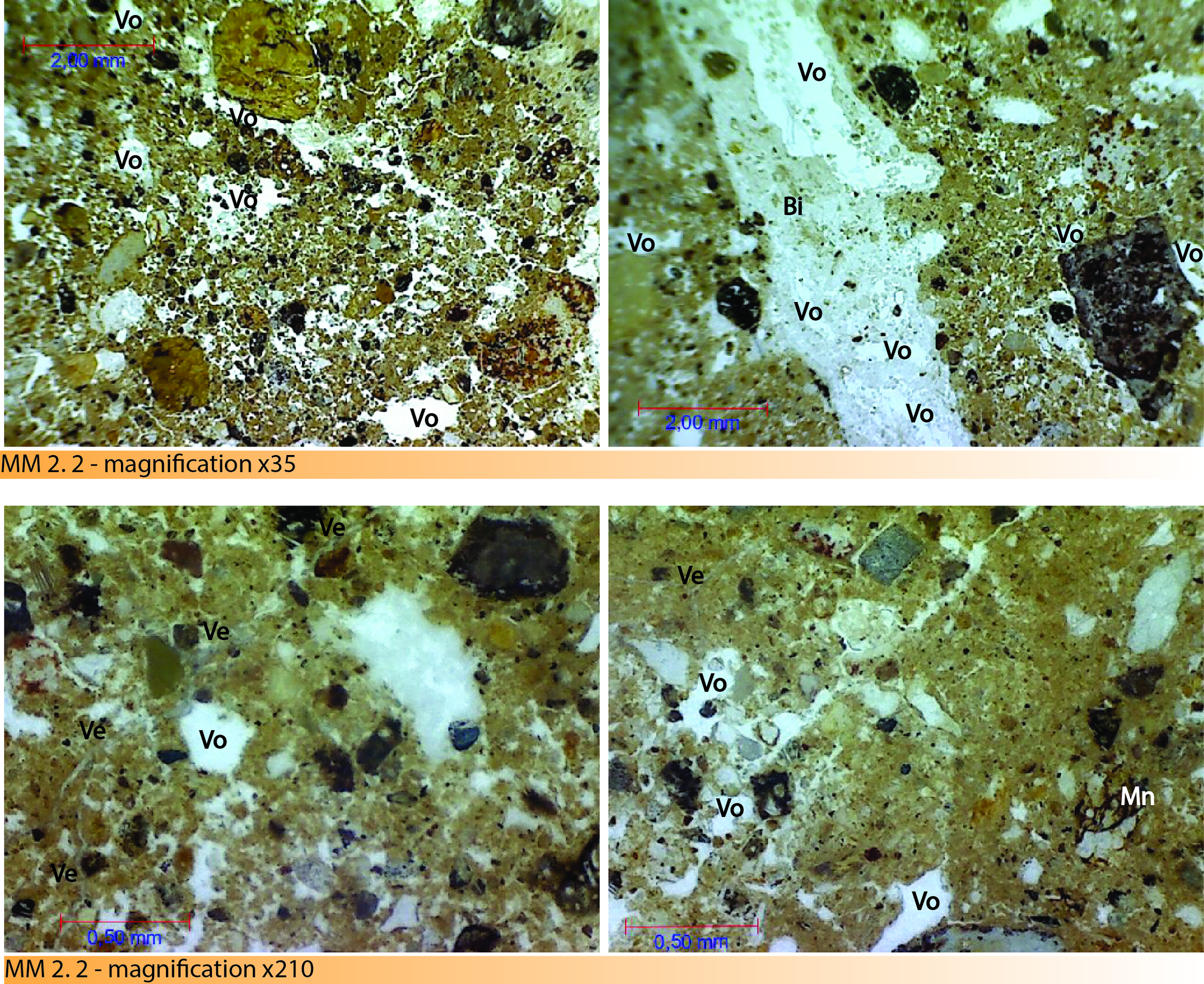

Supplement: S2 Fig — 1: pXRF results of elemental by trench. 2–9: Micromorphological samples pictures with details on the main micromorphological features: Ob = Obsidian flake; Bo = Bone; Co = Compaction traces; Vo = Void; Mn = Manganese; Bi = Bioturbation; Fm = Frost microstructure; Ve = Veins of Magnesium, gypsum and/or calcite; Rs = Rotation structure. 10: Odd-over-even predominance (OEP) and average chain length (ACL) of n-alkanes from Kalavan-2 (red triangles) compared to modern grass soils (yellow squares) and deciduous soils (green squares). Modern data from Bleidtner et al 2018. 11: n-alkane abundances of samples from the Kalavan-2 ~55kya sedimentary unit (red triangles) compared to modern grass soils (yellow squares) and deciduous soils (green squares). Modern data from Bleidtner et al 2018. (ZIP) [file pone.0245700.s006.zip › Sp 2 fig. 9 Kalavan MM 2.2 figure.tif]

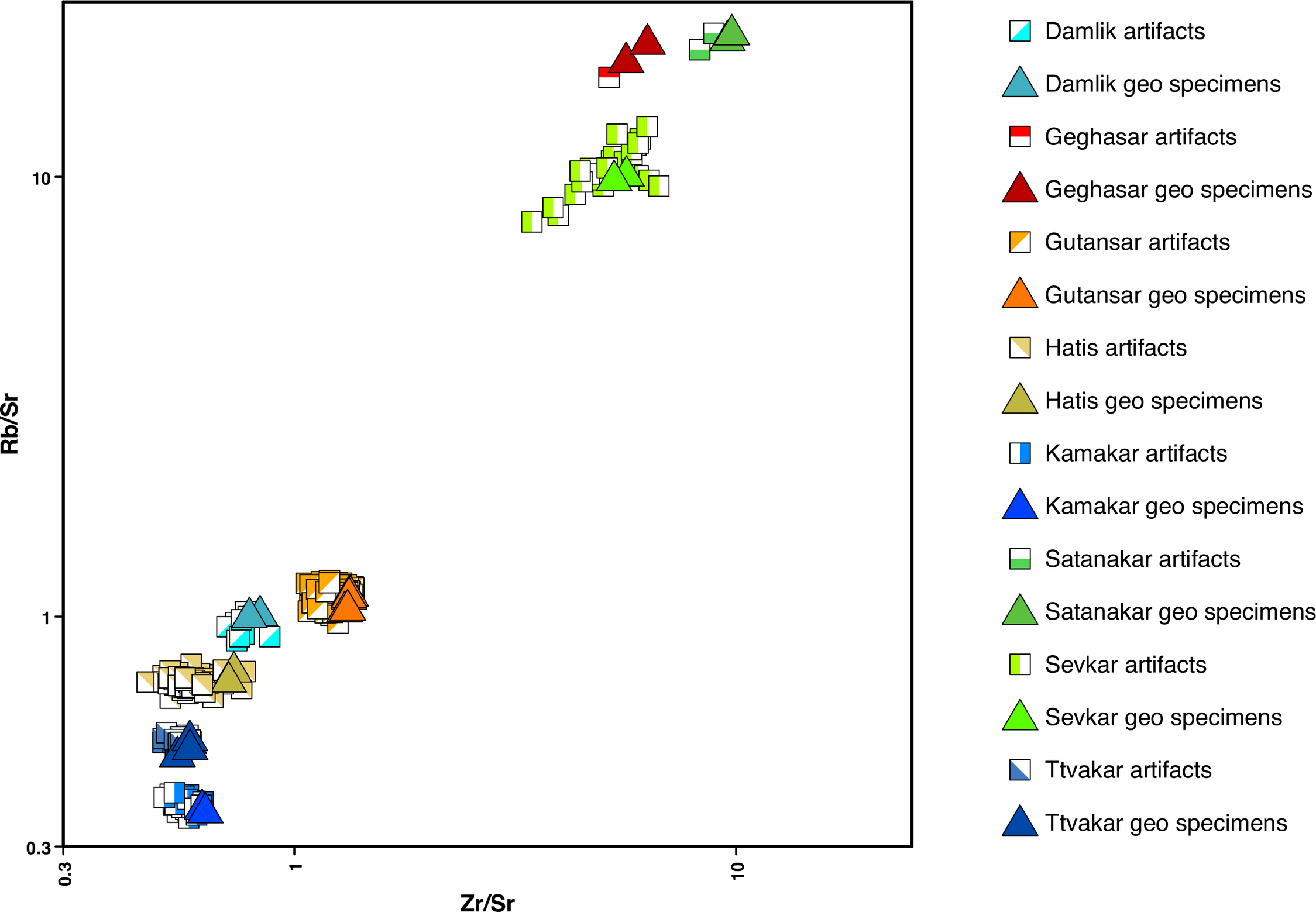

Supplement: S3 Fig — Reference specimens. S2 Fig 1 in S2 Fig. (TIF) [file pone.0245700.s007.tif]
