# Supplementary material for: Short-term occupations at high elevation during the Middle Paleolithic at Kalavan 2 (Republic of Armenia)
Source: PLoS One. 2021 Feb 4;16(2):e0245700. doi: 10.1371/journal.pone.0245700 (PMC7861461; doi:10.1371/journal.pone.0245700)
Supplement: S1 Table — 1. Summary of non-normalised glass compositional data obtained from the visible and crypto-tephra preserved in the Kalavan 2 Trench 1, 2 & 4 sequences. Visible tephra are affixed with T17- numbers whereas the cryptotephra begin T18- or T19. 2. Non-normalised glass major and minor element data. (ZIP) [file pone.0245700.s008.zip › S1 Table 1 Kalavan 2 tephra.docx]

**S1 Table 1:** Summary of non-normalised glass compositional data obtained from the visible and crypto-tephra preserved in the Kalavan 2 Trench 1, 2 & 4 sequences. Visible tephra are affixed with T17- numbers whereas the cryptotephra begin T18- or T19.

| **Lab code** | | **Trench #** | | **Composition** |  | **SiO2** | **TiO2** | **Al2O3** | **FeO** | **MnO** | **MgO** | **CaO** | **Na2O** | **K2O** | **P2O5** | **Total** |  |
| --- | --- | --- | --- | --- | --- | --- | --- | --- | --- | --- | --- | --- | --- | --- | --- | --- | --- |
|  | | | | | | | | | | | | | | | | | |
| T17-0174_a | | 2 | | Trachyandesite | Mean (18) | 57.78 | 1.60 | 16.09 | 7.11 | 0.13 | 2.62 | 5.46 | 4.90 | 3.01 | 0.71 | 99.42 |  |
|  |  |  |  |  | 2σ | 3.06 | 0.60 | 2.95 | 1.88 | 0.06 | 0.94 | 2.20 | 0.64 | 1.45 | 0.31 | 1.76 |  |
|  | | | | | | | | | | | | | | | | | |
| T17-0174_b | | 2 | | Trachyte/ Trachydacite | (1) | 66.20 | 0.32 | 14.29 | 3.86 | 0.15 | 0.01 | 0.71 | 6.43 | 5.07 | 0.02 | 97.07 |  |
|  | | | | | | | | | | | | | | | | | |
| T17-0172 | | 4 | | Trachyandesite | Mean (18) | 58.76 | 1.29 | 15.63 | 5.71 | 0.14 | 2.96 | 4.91 | 4.88 | 3.68 | 0.85 | 98.80 |  |
|  |  |  |  |  | 2σ | 1.28 | 0.16 | 0.72 | 0.55 | 0.02 | 0.42 | 0.88 | 0.44 | 0.92 | 0.13 | 1.98 |  |
|  | | | | | | | | | | | | | | | | | |
| T17-0173 | | 4 | | Trachyandesite-trachyte | Mean (17) | 61.14 | 1.07 | 16.22 | 4.80 | 0.11 | 1.99 | 4.29 | 5.13 | 3.47 | 0.64 | 98.85 |  |
|  |  |  |  |  | 2σ | 3.12 | 0.42 | 2.99 | 1.77 | 0.04 | 1.22 | 1.70 | 1.19 | 1.61 | 0.23 | 2.97 |  |
|  | | | | | | | | | | | | | | | | | |
| T17-0173_b | | 4 | | Rhyolite | Mean (2) | 73.52 | 0.43 | 11.10 | 2.13 | 0.05 | 0.48 | 0.97 | 3.39 | 4.82 | 0.06 | 96.96 |  |
|  |  |  |  |  | 2σ | 4.37 | 0.02 | 4.07 | 2.04 | 0.00 | 0.59 | 0.03 | 0.89 | 0.85 | 0.01 | 1.16 |  |
|  | | | | | | | | | | | | | | | | | |
| T18-0098_a | | 1 | | Rhyolite (peralkaline) | Mean (3) | 67.98 | 0.27 | 12.08 | 3.75 | 0.12 | 0.01 | 0.47 | 5.87 | 4.76 | 0.01 | 95.33 |  |
|  |  |  |  |  | 2σ | 4.86 | 0.10 | 2.82 | 0.24 | 0.05 | 0.03 | 0.51 | 0.08 | 0.76 | 0.01 | 0.85 |  |
|  | | | | | | | | | | | | | | | | | |
| T18-0098_b | | 1 | | Rhyolite  (calc-alkaline) | (1) | 74.71 | 0.06 | 12.67 | 1.25 | 0.04 | 0.03 | 0.50 | 3.94 | 4.88 | 0.02 | 98.09 |  |
|  | | | | | | | | | | | | | | | | | |
| T18-0095_a | | 1 | | Rhyolite (peralkaline) | Mean (6) | 68.14 | 0.31 | 11.56 | 4.48 | 0.14 | 0.01 | 0.43 | 5.98 | 4.56 | 0.02 | 95.61 |  |
|  |  |  |  |  | 2σ | 3.83 | 0.12 | 2.44 | 1.60 | 0.05 | 0.03 | 0.42 | 0.57 | 0.71 | 0.02 | 1.47 |  |
|  | | | | | | | | | | | | | | | | | |
| T18-0095_b | | 1 | | Rhyolite  (calc-alkaline) | (1) | 75.39 | 0.16 | 13.41 | 0.63 | 0.06 | 0.24 | 0.94 | 4.67 | 4.18 | 0.05 | 97.93 |  |
|  | | | | | | | | | | | | | | | | | |
| T18-0110_a | | 2 | | Rhyolite (peralkaline) | Mean (6) | 68.74 | 0.35 | 10.79 | 5.08 | 0.16 | 0.02 | 0.34 | 6.05 | 4.44 | 0.01 | 95.99 |  |
|  |  |  |  |  | 2σ | 1.28 | 0.03 | 0.69 | 0.85 | 0.06 | 0.11 | 0.06 | 0.71 | 0.33 | 0.01 | 1.79 |  |
|  | | | | | | | | | | | | | | | | | |
| T18-0110_b | | 2 | | Rhyolite  (calc-alkaline) | Mean (4) | 73.89 | 0.07 | 13.14 | 0.73 | 0.06 | 0.05 | 0.60 | 4.03 | 4.51 | 0.02 | 97.10 |  |
|  |  |  |  |  | 2σ | 3.99 | 0.02 | 1.03 | 0.85 | 0.07 | 0.05 | 0.40 | 0.66 | 0.80 | 0.02 | 2.26 |  |
|  | | | | | | | | | | | | | | | | | |
| T18-0110_c | | 2 | | Rhyolite  (calc-alkaline) | (1) | 69.48 | 0.71 | 13.26 | 2.88 | 0.06 | 0.65 | 2.04 | 3.69 | 4.23 | 0.11 | 97.12 |  |
|  | | | | | | | | | | | | | | | | | |
| T18-0106_a | | 2 | | Rhyolite (peralkaline) | Mean (5) | 71.34 | 0.27 | 10.17 | 4.47 | 0.12 | 0.01 | 0.22 | 5.57 | 4.26 | 0.01 | 96.43 |  |
|  |  |  |  |  | 2σ | 3.49 | 0.07 | 1.13 | 1.51 | 0.05 | 0.02 | 0.06 | 1.15 | 0.30 | 0.01 | 1.00 |  |
|  | | | | | | | | | | | | | | | | | |
| T18-0106_a | 2 | | Trachyte/ Trachydacite | | (1) | 65.30 | 0.90 | 14.61 | 3.87 | 0.08 | 1.25 | 3.46 | 4.95 | 2.64 | 0.24 | 97.30 |  |
|  | | | | | | | | | | | | | | | | | |
| T19-0105_a | | 2 | | Rhyolite (peralkaline) | Mean (6) | 68.87 | 0.35 | 10.69 | 5.21 | 0.15 | 0.01 | 0.32 | 6.20 | 4.33 | 0.01 | 96.14 |  |
|  |  |  |  |  | 2σ | 0.80 | 0.01 | 0.69 | 0.22 | 0.03 | 0.02 | 0.05 | 0.25 | 0.14 | 0.01 | 1.67 |  |
|  | | | | | | | | | | | | | | | | | |
| T19-0105_b | | 2 | | Rhyolite (peralkaline) | (1) | 70.48 | 0.22 | 10.83 | 3.66 | 0.09 | 0 | 0.22 | 5.58 | 4.37 | 0.01 | 95.46 |  |

**Figure 1**
Chemical classification diagrams of the glass analytical data obtained from the Kalavan tephras.

**Figure 2**
Glass shard concentrations vs. depth at Kalavan 2 Trench 1 & 2. Grey bars denote 10 cm scan samples and orange bars represent refined 2 cm intervals.

**Figure 3**
Selected chemical bi-plots of non-normalised glass compositional data from visible tephra identified at Kalavan 2. Comparisons are made to the Çekmece Formation derived from Nemrut in the EAVP. Comparative data from Macdonald et al. (2015).

**References**

Albert, P.G., Tomlinson, E.L., Smith, V.C., Di Traglia, F., Pistolesi, M., Morris, A., Donato, P., De Rosa, R., Sulpizio, R., Keller, J. and Rosi, M. 2017. Glass geochemistry of pyroclastic deposits from the Aeolian Islands in the last 50 ka: A proximal database for tephrochronology. *Journal of Volcanology and Geothermal Research*, 336, 81-107.

Arutyunyan, E.V., Lebedev, V.A., Chernyshev, I.V. and Sagatelyan, A.K. 2007. Geochronology of Neogene-Quaternary volcanism of the Geghama Highland (Lesser Caucasus, Armenia). *Doklady Earth Sciences* 416, 1042-1046.

Blockley, S.P.E., Pyne-O’Donnell, S.D.F., Lowe, J.J., Matthews, I.P., Stone, A., Pollard, A.M., Turney, C.S.M. and Molyneux, E.G. 2005. A new and less destructive laboratory procedure for the physical separation of distal glass tephra shards from sediments. *Quaternary Science Reviews* 24, 1952-1960.

Çubukçu, H.E., Ulusoy, İ., Aydar, E., Ersoy, O., Şen, E., Gourgaud, A. and Guillou, H. 2012. Mt. Nemrut volcano (Eastern Turkey): temporal petrological evolution. *Journal of Volcanology and Geothermal Research*, 209, 33-60.

Cullen, V.L., Smith, V.C. and Arz, H.W. 2014. The detailed tephrostratigraphy of a core from the south‐east Black Sea spanning the last∼ 60 ka. *Journal of Quaternary Science* 29, 675-690.

Davies, S.M., Abbott, P.M., Pearce, N.J., Wastegård, S. and Blockley, S.P. 2012. Integrating the INTIMATE records using tephrochronology: rising to the challenge. *Quaternary Science Reviews* 36, 11-27.

Deniel, C., Aydar, E. and Gourgaud, A. 1998. The Hasan Dagi stratovolcano (Central Anatolia, Turkey): evolution from calc-alkaline to alkaline magmatism in a collision zone. *Journal of Volcanology and Geothermal Research* 87, 275-302.

Druitt, T.H., Brenchley, P.J., Gökten, Y.E. and Francaviglia, V. 1995. Late Quaternary rhyolitic eruptions from the Acigöl Complex, central Turkey. *Journal of the Geological Society* 152, 655-667.

Hamann, Y., Wulf, S., Ersoy, O., Ehrmann, W., Aydar, E. and Schmiedl, G. 2010. First evidence of a distal early Holocene ash layer in Eastern Mediterranean deep-sea sediments derived from the Anatolian volcanic province. *Quaternary Research* 73, 497-506.

Hayward, C. 2012. High spatial resolution electron probe microanalysis of tephras and melt inclusions without beam-induced chemical modification. *The Holocene* 22, 119-125.

Jones, A.P., Tucker, M.E. and Hart, J. (eds.) (1999) The description & analysis of quaternary stratigraphic field sections, vol. 7, London, UK. Quaternary Research Association, 295pp. (Technical Guide, 7)

Karátson, D., Wulf, S., Veres, D., Magyari, E.K., Gertisser, R., Timar-Gabor, A., Novothny, Á., Telbisz, T., Szalai, Z., Anechitei-Deacu, V. and Appelt, O. 2016. The latest explosive eruptions of Ciomadul (Csomád) volcano, East Carpathians—a tephrostratigraphic approach for the 51–29 ka BP time interval. *Journal of Volcanology and Geothermal Research* 319, 29-51.

Keskin, M., 2007. Eastern Anatolia: a hotspot in a collision zone without a mantle plume. *Special Papers-Geological Society of America* 430, 693.

Kuzucuoglu, C., Pastre, J.F., Black, S., Ercan, T., Fontugne, M., Guillou, H., Hatté, C., Karabiyikoglu, M., Orth, P. and Türkecan, A. 1998. Identification and dating of tephra layers from Quaternary sedimentary sequences of Inner Anatolia, Turkey. *Journal of Volcanology and Geothermal Research* 85 (1-4), 153-172.

Landmann, G., Steinhauser, G., Sterba, J.H., Kempe, S. and Bichler, M. 2011. Geochemical fingerprints by activation analysis of tephra layers in Lake Van sediments, Turkey. *Applied Radiation and Isotopes* 69 (7), 929-935.

Lane, C.S., Cullen, V.L., White, D., Bramham-Law, C.W.F. and Smith, V.C., 2014. Cryptotephra as a dating and correlation tool in archaeology. *Journal of Archaeological Science* 42, 42-50.

Lebedev, V.A., Chernyshev, I.V. and Yakushev, A.I. 2011. Initial time and duration of Quaternary magmatism in the Aragats neovolcanic area (Lesser Caucasus, Armenia). *Doklady Earth Sciences* 437, (2) 532-536.

Lebedev, V.A., Chernyshev, I.V., Shatagin, K.N., Bubnov, S.N. and Yakushev, A.I. 2013. The quaternary volcanic rocks of the Geghama highland, Lesser Caucasus, Armenia: Geochronology, isotopic Sr-Nd characteristics, and origin. *Journal of Volcanology and Seismology* 7, 204-229.

Lebedev, V.A., Sharkov, E.V., Ünal, E. and Keskin, M. 2016a. Late Pleistocene Tendürek volcano (Eastern Anatolia, Turkey): I. Geochronology and petrographic characteristics of igneous rocks. *Petrology* 24, 127-152.

Lebedev, V.A., Chugaev, A.V., Ünal, E., Sharkov, E.V. and Keskin, M. 2016b. Late Pleistocene Tendürek Volcano (Eastern Anatolia, Turkey). II. Geochemistry and petrogenesis of the rocks. *Petrology* 24, 234-270.

Lowe, J.J., Ramsey, C.B., Housley, R.A., Lane, C.S., Tomlinson, E.L., RESET Team., RESET Associates. 2015. The RESET project: constructing a European tephra lattice for refined synchronisation of environmental and archaeological events during the last c. 100 ka. *Quaternary Science Reviews* 118, 1-17.

Macdonald, R., Sumita, M., Schmincke, H.U., Bagiński, B., White, J.C. and Ilnicki, S.S. 2015. Peralkaline felsic magmatism at the Nemrut volcano, Turkey: impact of volcanism on the evolution of Lake Van (Anatolia) IV. *Contributions to Mineralogy and Petrology* 169, 34.

Özdemir and Güleç, N. 2013. Geological and Geochemical Evolution of the Quaternary Suphan Stratovolcano, Eastern Anatolia, Turkey: Evidence for the Lithosphere-Asthenosphere Interaction in Post-Collisional Volcanism. *Journal of Petrology* 55, 37-62.

Pearce, J.A., Bender, J.F., De Long, S.E., Kidd, W.S.F., Low, P.J., Güner, Y., Saroglu, F., Yilmaz, Y., Moorbath, S. and Mitchell, J.G. 1990. Genesis of collision volcanism in Eastern Anatolia, Turkey. *Journal of Volcanology and Geothermal Research* 44, 189-229.

Schmincke, H.U., Sumita, M. and Paleovan scientific team. 2014. Impact of volcanism on the evolution of Lake Van (eastern Anatolia) III: periodic (Nemrut) vs. episodic (Süphan) explosive eruptions and climate forcing reflected in a tephra gap between ca. 14 ka and ca. 30 ka. *Journal of Volcanology and Geothermal Research* 285, 195-213.

Schmitt, A.K., Danišík, M., Evans, N.J., Siebel, W., Kiemele, E., Aydin, F. and Harvey, J.C. (2011). Acigöl rhyolite field, Central Anatolia (part 1): high-resolution dating of eruption episodes and zircon growth rates. *Contributions to Mineralogy and Petrology* 162, 1215-1231.

Şengör, A.M.C., Özeren, S., Genç, T. and Zor, E.,2003. East Anatolian high plateau as a mantle‐supported, north‐south shortened domal structure. *Geophysical Research Letters* 30.

Slimak, L., Kuhn, S.L., Roche, H., Mouralis, D., Buitenhuis, H., Balkan-Atlı, N., Binder, D., Kuzucuoğlu, C. and Guillou, H. 2008. Kaletepe Deresi 3 (Turkey): Archaeological evidence for early human settlement in Central Anatolia. *Journal of Human Evolution* 54, 99-111.

Sumita, M. and Schmincke, H.U. 2013a. Impact of volcanism on the evolution of Lake Van I: evolution of explosive volcanism of Nemrut Volcano (eastern Anatolia) during the past> 400,000 years. *Bulletin of volcanology* 75, 714.

Sumita, M. and Schmincke, H.U. 2013b. Impact of volcanism on the evolution of Lake Van II: temporal evolution of explosive volcanism of Nemrut Volcano (eastern Anatolia) during the past ca. 0.4 Ma. *Journal of Volcanology and Geothermal Research* 253, 15-34.

Szakács, A., Seghedi, I., Pécskay, Z. and Mirea, V., 2015. Eruptive history of a low-frequency and low-output rate Pleistocene volcano, Ciomadul, South Harghita Mts., Romania. *Bulletin of Volcanology* 77, (2) 12.

Timms, R.G.O., Matthews, I.P., Lowe, J.J., Palmer, A.P., Weston, D.J., MacLeod, A. and Blockley, S.P. 2019. Establishing tephrostratigraphic frameworks to aid the study of abrupt climatic and glacial transitions: a case study of the Last Glacial-Interglacial Transition in the British Isles (c. 16-8 ka BP). *Earth-Science Reviews*.

Tomlinson, E. L., Smith, V. C., Albert, P. G., Aydar, E., Civetta, L., Cioni, R., Çubukçu, C., Gertisser, R., Isaia, R., Menzies, M.A., Orsi, G., Rosi, M., Zanchetta, G. 2015. The major and trace element glass compositions of the productive Mediterranean volcanic sources: tools for correlating distal tephra layers in and around Europe. *Quaternary Science Reviews* 118, 48-66.

Tryon, C. A., Logan, M. A. V., Mouralis, D., Kuhn, S., Slimak, L., and Balkan-Atlı, N. 2009. Building a tephrostratigraphic framework for the Paleolithic of central Anatolia, Turkey. *Journal of Archaeological Science* 36, 637-652.

Wulf, S., Fedorowicz, S., Veres, D., Łanczont, M., Karátson, D., Gertisser, R., Bormann, M., Magyari, E., Appelt, O., Hambach, U. and Gozhyk, P.F. 2016. The ‘Roxolany Tephra’ (Ukraine)− new evidence for an origin from Ciomadul volcano, East Carpathians. *Journal of Quaternary Science* 31, 565-576.

Wulf, S., Hardiman, M.J., Staff, R.A., Koutsodendris, A., Appelt, O., Blockley, S.P., Lowe, J.J., Manning, C.J., Ottolini, L., Schmitt, A.K., Smith, V.C., Tomlinson, E.L., Vakhrameeva, P., Knupping, M., Kotthoff, U., Milner, A.M., Müller, U.C., Christanis, K., Kalaitzidis, S., Tzedakis, P.C., Schmiedl, G. and Pross, J. 2018. The marine isotope stage 1–5 cryptotephra record of Tenaghi Philippon, Greece: Towards a detailed tephrostratigraphic framework for the Eastern Mediterranean region. *Quaternary Science Reviews* 186, pp.236-262.

Yılmaz, Y., Güner, Y. and Şaroğlu, F. 1998. Geology of the Quaternary volcanic centres of the East Anatolia. *Journal of Volcanology and Geothermal Research* 85, 173-210.
