# Supplementary material for: Short-term occupations at high elevation during the Middle Paleolithic at Kalavan 2 (Republic of Armenia)
Source: PLoS One. 2021 Feb 4;16(2):e0245700. doi: 10.1371/journal.pone.0245700 (PMC7861461; doi:10.1371/journal.pone.0245700)
Supplement: S2 Table — 1: pXRF results of elements by trench. 2: Micromorphological results. 3: Pollen results. (ZIP) [file pone.0245700.s009.zip › S2 Table 1 list of elements pXRF.docx]

**S2 Table 1:** List of pXRF elements

| **Elements** | **T1** | **Min-Max** | **T2** | **Min-Max** | **T3** | **Min-Max** | **T4** | **Min-Max** |
| --- | --- | --- | --- | --- | --- | --- | --- | --- |
| **LE** | **64,23%** | 57,30-81,3% | **68,81%** | 64,2-80,57% | **85,1%** | 83,6-87,53% | **82,37%** | 65,5-88,75% |
| **Si** | **18%** | 10,45-23,65% | **15,02%** | 8,44-18,40% | **4,32%** | 3,54-5,75%- | **5,49%** | 2,54-15,06% |
| **Al** | **6,17%** | 0-8,45% | **5,57%** | 2,84-6,77% | **1,47%** | 1,17-1,74% | **1,86%** | 1,06-5,38% |
| **Ca** | **4,31%** | 1,35-12,54% | **4,12%** | 1,22-9,15% | **3,39%** | 1,12-6,48% | **4.87%** | 2,09-14,30% |
| **Fe** | **3,8%** | 2,87-5% | **4,04%** | 3,14-5,29% | **3,59%** | 3,26-4,32% | **3,44%** | 1,93-4,12% |
| **K** | **1,17%** | 0,69-2,44% | **1,01%** | 0,69-1,40% | **0,42%** | 0,62-1% | **0,83%** | 0,40-1,19% |
| **Mg** | **1,12%** | 1,2-1,9% | **0,55%** | 1,5-2,3% | **0,22%** | 0-1,8% | **0,44%** | 1,3-2,1% |
