# Supplementary material for: Short-term occupations at high elevation during the Middle Paleolithic at Kalavan 2 (Republic of Armenia)
Source: PLoS One. 2021 Feb 4;16(2):e0245700. doi: 10.1371/journal.pone.0245700 (PMC7861461; doi:10.1371/journal.pone.0245700)
Supplement: S2 Table — 1: pXRF results of elements by trench. 2: Micromorphological results. 3: Pollen results. (ZIP) [file pone.0245700.s009.zip › S2 Table 2 - Micromorph results.docx]

| **Sample** | **Trench** | **Level** | **Magnifica-tion** | **Structural and textural characters** | **Soil Components and features** | **Interpretations** |
| --- | --- | --- | --- | --- | --- | --- |
| **MM 3.1** | T1 | 1b | x35 & x210 | The basic distribution pattern is more frequently random but some areas are more interlaced. The distribution between coarse and fine (c/f) material is between double spaced enaulic and double spaced porphyric depending on the observed area at various magnification. The abundance of grain into the matrix is ranked between 20% and 30%. Grains have blocky shapes. Grains are also smooth rounded or subrounded and sometimes mixed with smooth subangular to angular material. The types of voids and microstructures are ranked between vesicles and channels and mainly represent roots activity. | Mineral grains and rock fragments are heterogeneous and mainly constituted by volcanogenic material. Metamorphic and sedimentary rock fragments are also present. Very diffuse organic material remains are visible in the micromass. Microfragment of obsidian flake and bone are visible. Mineral and rock alteration is light. | Alluvial deposits mixed with light colluvial supply and week traces of pedogenetic evolution (at the top of the thin section) and bioturbation. Distal alluvial phase with low energy deposition. A discreet colluviation and/or reworking of angular to sub-angular grains is effective during the flood stage due to the proximity of the slopes. Some rare evidence of compaction of the sediment in few areas indicates a short term top soil position. |
| **MM 3.2** | T1 | 1b | x35 & x210 | Random distribution pattern with more coarse material. The type of voids is vesicles, they are less numerous than in MM 3.1 and without traces of roots activities. | Same mineral components and features as in MM 3.1 but with diffuse manganese in the micromass and more floating grain distribution. | Alluvial and colluvial deposit with micro-lenses and recording of the variation movement of the water table (Mn deposition). |
| **MM 4.1** | T1 | 5 | x35 & x210 | The basic distribution pattern is more frequently random. The related distribution varies between double spaced fine enaulic and single to double spaced porphyric depending of the magnification scale of observation. Abundance of grain into the matrix is ranked between 20% and 35%. Grains have blocky shapes and are smooth rounded or subrounded mixed with smooth subangular to angular material. The types of voids and microstructures are ranked between vesicles, channels and planes. They mainly represent roots activity and bioturbation by worm gallery. | Mineral grains and rock fragments are heterogenous and constituted by volcanogenic, metamorphic and sedimentary material. Mineral and rock alteration is light. | Alluvial and colluvial deposit with medium dynamic of transportation. The bioturbation observed indicate a stasis in the morphogenic activity but without significative pedogenetic development. |
| **MM 4.2** | T1 | 5 | x35 & x210 | Random general distribution pattern. Coarse/fine related distribution is double spaced equal enaulic. The abundance of grain into the matrix is ranked between 20% and 40%. Grains have blocky shapes and are smooth rounded or subrounded mixed with smooth subangular material. Particles are poorly sorted and most of them have a blocky shape with smooth subrounded to subangular elements. Peds are subangular and blocky. The types of voids and microstructures are ranked between vesicles, channels, and planes. They mainly represent a reworking of soil elements or aggregate. | Same mineral components and features as in MM 4.1 but with secondary veins of gypsum/calcite or magnesium (see pXRF results) in the micromass and more floating grain distribution excepted for some sandy lenses. | Alluvial mixed with colluvial deposit with medium dynamic of transportation. The bioturbation observed is quite low and reworked soil aggregates are more present. These aggregates are however not related to a significative pedogenetic development. The alluvial component remains dominant. |
| **MM 1.1** | T2 | 4 | x35 & x210 | Random basic distribution pattern. Coarse/fine related distribution is double spaced porphyric to open porphyric. Abundance of grain into the matrix is ranked around 20%. Grains have blocky shapes and are smooth rounded or subrounded. Particles are poorly sorted and most of them have a blocky shape with smooth subrounded to rounded elements. Some particle shapes are platy to rodlike. The types of voids and microstructures are ranked between vesicles and channels and mainly represent roots activity (exceptionally planar voids can be observed in some pocket area). | Mineral grains and rock fragments are heterogeneous and constituted by volcanogenic, metamorphic, and sedimentary material. Mineral and rock alteration is light. Secondary veins of gypsum/calcite or magnesium (see pXRF results) are observed with x210 magnification. | Alluvial deposits mixed with light colluvial supply and traces of light pedogenetic evolution and bioturbation. Distal alluvial phase with low energy deposition. Some of the grains indicate the stream orientation (from left to right on the photo) A discreet colluviation and/or reworking of sub-angular grains is effective during the flood stage due to the proximity of the slopes. Some evidence of compaction of the sediment indicate a short term top soil position as well as the development of secondary crystallization. |
| **MM 1.2** | T2 | 4 | x35 & x210 | Random basic distribution pattern. Coarse/fine related distribution is open porphyric, most rarely double spaced porphyric. The abundance of grain into the matrix is ranked around 5% to 10%. Grains have blocky shapes and are smoothly rounded, subrounded, and sometimes subangular. Particles are poorly sorted and most of them have a blocky shape with smooth subrounded to rounded elements. The types of voids and microstructures are more often vesicles but some channels can be observed. They mainly represent roots activity with little compaction of the sediment. | Mineral grains and rock fragments are heterogeneous and constituted by volcanogenic, metamorphic, and sedimentary material. Mineral and rock alteration is light. Secondary veins of gypsum/calcite or magnesium (see pXRF results) are observed with x210 magnification. Microfragment of some subangular bones are visible. | Alluvial deposits are mixed with light colluvial supply and traces of light pedogenetic evolution and bioturbation (mainly roots). Distal alluvial phase with low energy deposition. Some of the grains indicate the stream orientation (from left to right on the photo) A discreet colluviation and/or reworking of sub-angular grains is effective during the flood stage due to the proximity of the slopes. As for the MM 1.1 observations, some evidence of light compaction of the sediment indicate a short term top soil position as well as the development of secondary crystallization. |
| **MM 2.1** | T2 | 5 | x35 & x210 | Random to clustered basic distribution patterns. Depending on the various magnification, the coarse/fine grain distribution can be ranked between single-spaced porphyric to double spaced equal enaulic. The abundance of constituents is around 20% to 30%. They are poorly sorted and with blocky shape. Most of the particles are smooth or undulating and always subrounded. Voids are from compounds packing type or plane type. The light rotation structure characterizes this sample. | Mineral grains and rock fragments are heterogeneous and constituted by volcanogenic, metamorphic, and sedimentary material. Mineral and rock alteration shows grain crushing, grain stacks, and smooth fractures typical of frost microstructures. | Mainly alluvial deposit with medium dynamic of transportation. After deposition and a short period of morphogenic stasis, the sediment was affected by preliminary to medium frost action (short to medium time span). This part of the thin section corresponds to the level 4 topsoil transition with mixed vegetation cover. This could be just a preservation of weather trends more than a long term glacial climate interval. |
| **MM 2.2** | T2 | 5 | x35 & x210 | Random to clustered basic distribution patterns. Depending on the various magnification, the coarse/fine grain distribution can be ranked between single-spaced porphyric to double spaced equal enaulic. The abundance of constituents is around 5% to 30% (highest rates are in the sandy lenses). They are poorly sorted and with blocky shape. The particles are mainly subrounded. Reworked sub-angular blocky peds are visible. Voids are from compounds packing type or plane type. Channel microstructure due to bioturbation (worm gallery) are observable. | Mineral grains and rock fragments are heterogeneous and constituted by volcanogenic, metamorphic, and sedimentary material. Mineral and rock alteration is light to moderate. | Mainly alluvial deposit with medium dynamic of transportation. Sandy lenses underline the alluvial components. Reworked soil peds and bioturbation indicates a light post-depositional pedogenic development lightly impacted by surface erosion. |
