# Supplementary material for: Short-term occupations at high elevation during the Middle Paleolithic at Kalavan 2 (Republic of Armenia)
Source: PLoS One. 2021 Feb 4;16(2):e0245700. doi: 10.1371/journal.pone.0245700 (PMC7861461; doi:10.1371/journal.pone.0245700)
Supplement: S2 Table — 1: pXRF results of elements by trench. 2: Micromorphological results. 3: Pollen results. (ZIP) [file pone.0245700.s009.zip › S2 Table 3 pollen results.docx]

| **Sample depth** | **K2T4U5d**  **N** | **K2T4U5d**  **%** | **K2T4U5a**  **N** | **K2T4U5a**  **%** |
| --- | --- | --- | --- | --- |
| **Pollen sum** | **169.5** | **100%** | **175.5** | **100%** |
| **No of taxa** | **11** | **11** | **12** | **12** |
| *Pinus diploxylon* | 2.5 | **1.5%** | 0.5 | **0.3%** |
| *Fraxinus excelsior* | 3 | 1.8% | 1 | 0.6% |
| **Tree** | **5.5** | **3.2%** | **1.5** | **0.9%** |
| *Cupressus* |  | 0.0% | 1 | 0.6% |
| **Shrub** | **0** | **0.0%** | **1** | **0.6%** |
| *Ephedra distachia* | 3 | 1.8% |  | 0.0% |
| **Heath** | **3** | 1.8% | **0** | **0.0%** |
| Poaceae | 8 | 4.7% | 2 | 1.1% |
| Asteraceae Asteroideae | 12 | 7.1% | 12 | 6.8% |
| Asteraceae Chicoroideae | 106 | 62.5% | 26 | 14.8% |
| *Convolvulus* | 2 | 1.2% | 2 | 1.1% |
| *Centaurea* undiff | 8 | **4.7%** | 97 | **55.3%** |
| *Lychnis flos-cuculi* |  | 0.0% | 1 | 0.6% |
| Caryophyllaceae undiff | 9 | 5.3% | 4 | 2.3% |
| Chenopodiaceae | 12 | **7.1%** | 28 | **16.0%** |
| *Artemisia* | 4 | **2.4%** |  | **0.0%** |
| *Ranunculus* undiff |  | 0.0% | 1 | 0.6% |
| **Herb** | **161** | **95.0%** | **173** | **98.6%** |
