# Supplementary material for: Short-term occupations at high elevation during the Middle Paleolithic at Kalavan 2 (Republic of Armenia)
Source: PLoS One. 2021 Feb 4;16(2):e0245700. doi: 10.1371/journal.pone.0245700 (PMC7861461; doi:10.1371/journal.pone.0245700)
Supplement: S3 Table — (DOCX) [file pone.0245700.s010.docx]

| Trench | Raw material | Type | Edge damage | Macro trace | Micro wear | Use-wear traces | surface | material | movement |
| --- | --- | --- | --- | --- | --- | --- | --- | --- | --- |
| 1 | Obsidian | Point proximal frag | yes | no | no | - | - | - | - |
| 1 | Obsidian | Chip | no | no | no | - | - | - | - |
| 1 | Obsidian | Chip | yes | no | no | - | - | - | - |
| 1 | Obsidian | Chip | no | no | no | - | - | - | - |
| 1 | Obsidian | Chip | no | no | no | - | - | - | - |
| 1 | Obsidian | Chip | yes | no | no | - | - | - | - |
| 1 | Obsidian | Chip | yes | no | no | - | - | - | - |
| 1 | Obsidian | Chip | yes | no | no | - | - | - | - |
| 1 | Obsidian | Chip | no | no | no | - | - | - | - |
| 1 | Obsidian | Chip | no | no | no | - | - | - | - |
| 1 | Obsidian | Chip | no | no | no | - | - | - | - |
| 1 | Obsidian | Chip | no | no | no | - | - | - | - |
| 1 | Obsidian | Shaping flake | no | no | no | polish | ventral and dorsal | undetermined | cutting |
| 1 | Obsidian | Chip | no | no | no | - | - | - | - |
| 1 | Obsidian | Chip | yes | no | no | - | - | - | - |
| 1 | Obsidian | Chip | yes | no | no | - | - | - | - |
| 1 | Obsidian | Chip | no | no | no | - | - | - | - |
| 1 | Obsidian | Chip | no | no | no | - | - | - | - |
| 1 | Obsidian | Chip | no | no | no | - | - | - | - |
| 1 | Obsidian | Chip | no | no | no | - | - | - | - |
| 1 | Obsidian | Chip | yes | no | no | - | - | - | - |
| 1 | Obsidian | Chip | no | no | no | - | - | - | - |
| 1 | Non-obsidian | Core-chopper | no | Yes | no | - | - | - | - |
| 1 | Non-obsidian | Pebble | no | no | no | - | - | - | - |
| 2 | Obsidian | Shaping flake | yes | no | yes | polish | ventral and dorsal | Semi-hard | cutting |
| 2 | Obsidian | Point distal frag | yes | no | yes | polish | ventral | wood | cutting |
| 2 | Obsidian | Retouched flake | yes | no | yes | polish and striations | ventral | Semi-hard | undetermined |
| 2 | Obsidian | Retouched flake | yes | no | yes | polish | ventral | undetermined | undetermined |
| 2 | Obsidian | Shaping flake | yes | no | no | - | - | - | - |
| 2 | Obsidian | Shaping flake | no | no | yes | polish | ventral | wood | scraping |
| 2 | Obsidian | Retouched flake | yes | no | no | - | - | - | - |
| 2 | Obsidian | Retouched flake | yes | no | yes | polish | ventral and dorsal | wood | cutting |
| 2 | Obsidian | Flake | no | no | no | - | - | - | - |
| 2 | Obsidian | Flake | yes | no | yes | polish | ventral | wood | scraping |
| 2 | Obsidian | Chip | yes | no | no | - | - | - | - |
| 2 | Obsidian | Shaping flake | yes | no | yes | polish | ventral | wood | scraping |
| 2 | Non-obsidian | Flake | yes | no | no | - | - | - | - |
| 2 | Non-obsidian | Flake | yes | no | no | - | - | - | - |
| 2 | Non-obsidian | Flake | no | no | no | - | - | - | - |
| 2 | Non-obsidian | Flake | yes | no | no | - | - | - | - |
| 2 | Non-obsidian | Flake | yes | no | no | - | - | - | - |
| 2 | Non-obsidian | Flake | no | no | no | - | - | - | - |
| 2 | Non-obsidian | Flake | no | no | no | - | - | - | - |
| 2 | Non-obsidian | Flake | no | no | no | - | - | - | - |

**S3 Table: list of studied samples for use-wear**
