# Supplementary material for: Short-term occupations at high elevation during the Middle Paleolithic at Kalavan 2 (Republic of Armenia)
Source: PLoS One. 2021 Feb 4;16(2):e0245700. doi: 10.1371/journal.pone.0245700 (PMC7861461; doi:10.1371/journal.pone.0245700)
Supplement: S4 Table — 1: Faunal size class categories. 2: Teeth suitable for study for mesowear and dental microwear texture analysis (DMTA). 3: Small mammal postcranial elements per trench. Trench 4 has been subdivided in units due to the richness of post-cranial elements. (ZIP) [file pone.0245700.s011.zip › S4 Table 1 fauna_size_class.docx]

| Size class | Weight range | Examples of taxa | Taxa at Kalavan 2 |
| --- | --- | --- | --- |
| 0 | Some gram, to a couple of 100 gram | Micro mammals |  |
| 1 | Less than and around 20 kg | Hare, fox, badger |  |
| 2 | 20 to around 120 kg | Small cervids (roe deer), goat, sheep | Capra/Ovis (e.g. Mouflon (25-87kg), wild goat (25-95kg), rupicapra (24-50kg)) |
| 3 | 120 to around 260 kg | Medium cervids (red deer, reindeer), fallow deer and boar. | Red deer (120-240kg),  Onager (200-260kg) |
| 4 | Around 300 to 1000 kg | Large bovids (bos, bison), horse | Bos/Bison, Horse |
| 5 | 1000 and more kg | Megaherbivores (Proboscidea, rhino) |  |
| 99 |  | other animals |  |

Sources:

Brain, C. K., 1981. *The Hunters or the Hunted? An Introduction to African Cave Taphonomy.* The University of Chicago Press: Chicago and London.

Gaudzinski-Windheuser, S., 2005. *Subbsistenzstrategien frühpleistozäner Hominiden in Eurasien. Taphonomische Faunenbetrachtungen der Fundstellen der `Ubediya Formation (Israel).* Monographien Bd. 61. Mainz: RGZM.

Isaac, G. L. & Isaac, B., ed., 1997. *Koobi Fora Research Project.* Volume 5. *Plio-Pleistocene Archaeology*. Clarendon Press: Oxford.

Heptner, V. G. et al., 1988-1992. *Mammals of the Soviet Union.* In three Volumes. Smithsonian Institution Libraries and The National Science Foundation: Washington D.C.

Hutchins, M. et al., 2003. *Grzimek’s Animal Life Encyclopedia*. 2nd Ed. Vol. 12-16 *(Mammals I-V).* Farmington Hills, MI, USA: Gale Group.

Kindler, L., 2012. *Die Rolle von Raubtieren bei der Einnischung und Subsistenz jungpleistozäner Neandertaler. Archäozoologie und Taphonomie der mittelpaläolithischen Fauna aus der Balver Höhle.* Monographien Bd. 99. Mainz: RGZM.

Nowak, R. M. & Paradiso, J. L., 1991. *Walker’s Mammals of the World*. Ed. 5. Baltimore: Johns Hopkins Univ. Press.
