# Supplementary material for: Short-term occupations at high elevation during the Middle Paleolithic at Kalavan 2 (Republic of Armenia)
Source: PLoS One. 2021 Feb 4;16(2):e0245700. doi: 10.1371/journal.pone.0245700 (PMC7861461; doi:10.1371/journal.pone.0245700)
Supplement: S4 Table — 1: Faunal size class categories. 2: Teeth suitable for study for mesowear and dental microwear texture analysis (DMTA). 3: Small mammal postcranial elements per trench. Trench 4 has been subdivided in units due to the richness of post-cranial elements. (ZIP) [file pone.0245700.s011.zip › S4 Table 2 meso and microwear.docx]

| **ID** | **Trench** | **Unit** | **Species** | **Preservation** | **Mesowear** | **DMTA** |
| --- | --- | --- | --- | --- | --- | --- |
| F6d-267 | 1 | 1b | *Bos*/*Bison* | OK |  | x |
| F7b-275 | 1 | 1b | *Bos*/*Bison* | Pieces | x |  |
| D79-17 | 1 | 5/6 | *Equus hemionus* | Broken enamel |  |  |
| K49-28 | 1 | 7 | *Equus* sp. | Pieces |  |  |
| L49-45 | 2 | 4 | *Cervus elaphus* | OK | x | x |
| J49-170 | 2 | 4 | *Equus hemionus* | OK | x | x |
| I48-34 | 2 | 4 | *Equus hemionus* | OK | x | x |
| J48-7 | 2 | 4 | *Equus* Sp. | Broken enamel |  |  |
| C3-U+C-114 | 3 | 2b | *Bos*/*Bison* | OK | x | x |
| C2a-138 | 3 | 2b | *Bos*/*Bison* | Individual too old |  |  |
|  |  |  |  | **n** | **5** | **5** |

S4 Table 2: Teeth suitable for study for mesowear and dental microwear texture analysis (DMTA).
