# Supplementary material for: Short-term occupations at high elevation during the Middle Paleolithic at Kalavan 2 (Republic of Armenia)
Source: PLoS One. 2021 Feb 4;16(2):e0245700. doi: 10.1371/journal.pone.0245700 (PMC7861461; doi:10.1371/journal.pone.0245700)
Supplement: S4 Table — 1: Faunal size class categories. 2: Teeth suitable for study for mesowear and dental microwear texture analysis (DMTA). 3: Small mammal postcranial elements per trench. Trench 4 has been subdivided in units due to the richness of post-cranial elements. (ZIP) [file pone.0245700.s011.zip › S4 Table 3 Small mammal postcranial elements per trench.docx]

|  | **T2**  Combined units | | **T3**  Combined units | |
| --- | --- | --- | --- | --- |
| Elements | Count | Unfused | Count | Unfused |
| Humerus | 2 | 1 |  |  |
| Radius | 2 |  | 1 |  |
| Ulna | 2 |  | 1 |  |
| Femur | 2 |  |  |  |
| Tibia | 4 |  |  |  |
| Metapodial | 2 |  |  |  |
| Longbone | 1 |  |  |  |
| Total | 15 | 1 | 2 |  |

| **Trench 4** | **Unit 2** | | **Unit 3** | | **Unit 4** | | **Unit 5a** | | **Unit 5b** | | **Unit 5c** | | | **Unit 5d** | | **Totals** |  |
| --- | --- | --- | --- | --- | --- | --- | --- | --- | --- | --- | --- | --- | --- | --- | --- | --- | --- |
| Elements | Count | Unfused | Count | Unfused | Count | Unfused | Count | Unfused | Count | Unfused | Count | Unfused | Count | | Unfused | Elements | Unfused |
| Clavicle | 1 |  |  |  |  |  |  |  |  |  |  |  |  | |  | 1 | 0 |
| Costae | 2 |  | 2 |  | 4 |  |  |  | 5 |  | 6 |  |  | |  | 19 | 0 |
| Vertebrae | 1 |  | 1 |  | 8 |  | 1 |  | 6 | 1 | 5 | 1 | 1 | |  | 23 | 2 |
| Scapula |  |  |  |  | 3 |  |  |  | 1 |  |  |  | 1 | |  | 5 | 0 |
| Humerus | 4 | 2 |  |  | 7 | 3 |  |  | 3 | 3 | 7 |  | 2 | | 1 | 23 | 9 |
| Radius | 1 |  | 1 |  | 1 |  |  |  |  |  | 3 |  |  | |  | 6 | 0 |
| Ulna |  |  |  |  | 7 |  |  |  | 4 |  | 1 |  |  | |  | 12 | 0 |
| Pelvis | 2 |  |  |  | 1 |  | 1 |  | 3 |  | 6 |  | 2 | |  | 15 | 0 |
| Femur | 5 | 1 | 3 |  | 6 | 2 |  |  | 6 | 2 | 2 | 2 | 2 | |  | 24 | 7 |
| Tibia |  |  | 1 | 1 | 7 | 4 |  |  | 6 | 1 | 2 | 1 | 5 | | 3 | 21 | 10 |
| Fibula |  |  |  |  | 1 |  |  |  |  |  | 1 | 1 |  | |  | 2 | 1 |
| Metapodial |  |  | 1 |  | 5 |  | 2 |  | 8 |  | 7 |  | 1 | |  | 24 | 0 |
| Longbone |  |  |  |  | 1 |  |  |  | 1 |  | 4 |  | 1 | |  | 7 | 0 |
| **Total** | 16 | 3 | 9 | 1 | 51 | 9 | 4 | 0 | 43 | 7 | 44 | 5 | 15 | | 4 | 182 | 29 |

S4 Table 3: Small mammal postcranial elements per trench. Trench 4 has been subdivided in units due to the richness of post-cranial elements.

| Trench 4 | Elements | Clavicle | Costae | Vertebrae | Scapula | Humerus | Radius | Ulna | Pelvis | Femur | Tibia | Fibula | Metapodial | Longbone | Total |
| --- | --- | --- | --- | --- | --- | --- | --- | --- | --- | --- | --- | --- | --- | --- | --- |
| Unit 2 | Count | 1 | 2 | 1 |  | 4 | 1 |  | 2 | 5 |  |  |  |  | 16 |
|  | Unfused |  |  |  |  | 2 |  |  |  | 1 |  |  |  |  | 3 |
| Unit 3 | Count |  | 2 | 1 |  |  | 1 |  |  | 3 | 1 |  | 1 |  | 9 |
|  | Unfused |  |  |  |  |  |  |  |  |  | 1 |  |  |  | 1 |
| Unit 4 | Count |  | 4 | 8 | 3 | 7 | 1 | 7 | 1 | 6 | 7 | 1 | 5 | 1 | 51 |
|  | Unfused |  |  |  |  | 3 |  |  |  | 2 | 4 |  |  |  | 9 |
| Unit 5a | Count |  |  | 1 |  |  |  |  | 1 |  |  |  | 2 |  | 4 |
|  | Unfused |  |  |  |  |  |  |  |  |  |  |  |  |  | 0 |
| Unit 5b | Count |  | 5 | 6 | 1 | 3 |  | 4 | 3 | 6 | 6 |  | 8 | 1 | 43 |
|  | Unfused |  |  | 1 |  | 3 |  |  |  | 2 | 1 |  |  |  | 7 |
| Unit 5c | Count |  | 6 | 5 |  | 7 | 3 | 1 | 6 | 2 | 2 | 1 | 7 | 4 | 44 |
|  | Unfused |  |  | 1 |  |  |  |  |  | 2 | 1 | 1 |  |  | 5 |
| Unit 5d | Count |  |  | 1 | 1 | 2 |  |  | 2 | 2 | 5 |  | 1 | 1 | 15 |
|  | Unfused |  |  |  |  | 1 |  |  |  |  | 3 |  |  |  | 4 |
| Totals | Elements | 1 | 19 | 23 | 5 | 23 | 6 | 12 | 15 | 24 | 21 | 2 | 24 | 7 | 182 |
|  | Unfused | 0 | 0 | 2 | 0 | 9 | 0 | 0 | 0 | 7 | 10 | 1 | 0 | 0 | 29 |
