# Supplementary material for: Short-term occupations at high elevation during the Middle Paleolithic at Kalavan 2 (Republic of Armenia)
Source: PLoS One. 2021 Feb 4;16(2):e0245700. doi: 10.1371/journal.pone.0245700 (PMC7861461; doi:10.1371/journal.pone.0245700)
Supplement: Attachment — Submitted filename: PONE-D-20-30812_reviewer.pdf [file pone.0245700.s012.pdf]

PLOS ONE

Short-term occupations at high elevation during the Middle Paleolithic at Kalavan 2 (Republic of Armenia)

--Manuscript Draft--

**Manuscript Number:**	PONE-D-20-30812
**Article Type:**	Research Article
**Full Title:**	Short-term occupations at high elevation during the Middle Paleolithic at Kalavan 2 (Republic of Armenia)
**Short Title:**	Short-term occupations at Kalavan 2
**Corresponding Author:**	Ariel Malinsky-Buller<br>Monrepos<br>Neuwied, <None> GERMANY
**Keywords:**	Middle Paleolithic, Armenian highland, lithic technology, subsistence strategies, settlement system, dating, paleoenvironmental reconstruction, seasonality
**Abstract:**	The Armenian highlands encompasses rugged and environmentally diverse landscapes and is characterized by a mosaic of distinct ecological niches and large temperature gradients. Strong seasonal fluctuations in resource availability along topographic gradients likely prompted Pleistocene hominin groups to adapt by adjusting their mobility strategies. However, the role that higher elevated landscapes played in hunter-gatherer settlement systems during the Late Pleistocene (Middle Palaeolithic [MP]) remains poorly understood. The MP site of Kalavan 2 (Armenia) is currently the only excavated open-air locality in the Armenian highlands with a stratified sequence that preserves faunal remains and lithic artifacts in association. At 1640 m above sea level, the site is ideally positioned for testing hypotheses involving elevation-dependent seasonal mobility and subsistence strategies. The results of our recent excavations and the analysis of chronologically and environmentally contextualized archaeological data suggest repeated ephemeral occupations, likely related to hunting in a high-elevation steppe landscape.
**Order of Authors:**	Ariel Malinsky-Buller
	Philip Glauberman
	Vincent Ollivier
	Tobias Lauer
	Rhys Timms
	Ellery Frahm
	Alexander Brittingham
	Benno Triller
	Lutz Kindler
	Monika V Knul
	Masha Krakovsky
	Sebastian Joannin
	Micheal Wren
	Olivier Bellier
	Alexander M Clark
	Simon P.E Blockley
	Dimidry Arakelyan
	Joao Marreiros

Powered by Editorial Manager® and ProduXion Manager® from Aries Systems Corporation

	Eduardo Paixao
	Ivan Calandra
	Robert Ghukasyan
	David Nora
	Nadav Nir
	Ani Adigyozalyan
	Hayk Haydosyan
	Boris Gasparyan
**Additional Information:**	
**Question**	**Response**
**Financial Disclosure**<br><br>Enter a financial disclosure statement that describes the sources of funding for the work included in this submission. Review the submission guidelines for detailed requirements. View published research articles from *PLOS ONE* for specific examples.<br><br>This statement is required for submission and **will appear in the published article** if the submission is accepted. Please make sure it is accurate.<br><br>**Unfunded studies**<br>Enter: *The author(s) received no specific funding for this work.*<br><br>**Funded studies**<br>Enter a statement with the following details:<br>• Initials of the authors who received each award<br>• Grant numbers awarded to each author<br>• The full name of each funder<br>• URL of each funder website<br>• Did the sponsors or funders play any role in the study design, data collection and analysis, decision to publish, or preparation of the manuscript?<br>• **NO** - Include this sentence at the end of your statement: *The funders had no role in study design, data collection and analysis, decision to publish, or preparation of the manuscript.*<br>• **YES** - Specify the role(s) played.<br><br>* typeset	For the essential financial and similar support, we gratefully thank the funding agencies of The Gerda Henkel Stiftung grant n. AZ 10_V_17 and n. AZ 23/F/19, the Leakey Foundation, and the Gfoeller Renaissance Foundation of USA. RT, AC, and SB would like to acknowledge support from the Leverhulme Trust-funded Palaeolithic Archaeology, Geochronology, and Environments of the Southern Caucasus (PAGES) project, and to thank Dr. C. Hayward at the University of Edinburgh for assistance with the microprobe analyses. VO et OB were supported by the ECCOREV Research Federation (Aix-Marseille Université, CNRS) and Labex OT-Med (n° ANR- 11-LABX-0061) funded by the French Government “Investissements d’Avenir” programme of the French National Research Agency (ANR) through the A*MIDEX project (n°ANR-11-IDEX-0001-02).
**Competing Interests**	The authors have declared that no competing interests exist.

Powered by Editorial Manager® and ProduXion Manager® from Aries Systems Corporation

Use the instructions below to enter a competing interest statement for this submission. On behalf of all authors, disclose any competing interests that could be perceived to bias this work—acknowledging all financial support and any other relevant financial or non-financial competing interests.<br><br>This statement **will appear in the published article** if the submission is accepted. Please make sure it is accurate. View published research articles from *PLOS ONE* for specific examples.<br><br>**NO authors have competing interests**<br><br>Enter: *The authors have declared that no competing interests exist.*<br><br>**Authors with competing interests**<br><br>Enter competing interest details beginning with this statement:<br><br>*I have read the journal's policy and the authors of this manuscript have the following competing interests: [insert competing interests here]*<br><br>* typeset	
**Ethics Statement**<br><br>Enter an ethics statement for this submission. This statement is required if the study involved:<br><br>• Human participants<br>• Human specimens or tissue<br>• Vertebrate animals or cephalopods<br>• Vertebrate embryos or tissues<br>• Field research<br><br>Write "N/A" if the submission does not require an ethics statement.<br><br>General guidance is provided below. Consult the submission guidelines for	N/A

Powered by Editorial Manager® and ProduXion Manager® from Aries Systems Corporation

detailed instructions. **Make sure that all information entered here is included in the Methods section of the manuscript.**<br><br>**Format for specific study types**<br><br>**Human Subject Research (involving human participants and/or tissue)**<br>• Give the name of the institutional review board or ethics committee that approved the study<br>• Include the approval number and/or a statement indicating approval of this research<br>• Indicate the form of consent obtained (written/oral) or the reason that consent was not obtained (e.g. the data were analyzed anonymously)<br><br>**Animal Research (involving vertebrate animals, embryos or tissues)**<br>• Provide the name of the Institutional Animal Care and Use Committee (IACUC) or other relevant ethics board that reviewed the study protocol, and indicate whether they approved this research or granted a formal waiver of ethical approval<br>• Include an approval number if one was obtained<br>• If the study involved *non-human primates*, add *additional details* about animal welfare and steps taken to ameliorate suffering<br>• If anesthesia, euthanasia, or any kind of animal sacrifice is part of the study, include briefly which substances and/or methods were applied<br><br>**Field Research**<br><br>Include the following details if this study involves the collection of plant, animal, or other materials from a natural setting:<br>• Field permit number<br>• Name of the institution or relevant body that granted permission	
**Data Availability**<br><br>Authors are required to make all data underlying the findings described fully available, without restriction, and from the time of publication. PLOS allows rare	Yes - all data are fully available without restriction

Powered by Editorial Manager® and ProduXion Manager® from Aries Systems Corporation

exceptions to address legal and ethical concerns. See the PLOS Data Policy and FAQ for detailed information.<br><br>A Data Availability Statement describing where the data can be found is required at submission. Your answers to this question constitute the Data Availability Statement and **will be published in the article**, if accepted.<br><br>**Important:** Stating 'data available on request from the author' is not sufficient. If your data are only available upon request, select 'No' for the first question and explain your exceptional situation in the text box.<br><br>Do the authors confirm that all data underlying the findings described in their manuscript are fully available without restriction?	
**Describe where the data may be found in full sentences. If you are copying our sample text, replace any instances of XXX with the appropriate details.**<br><br>• If the data are **held or will be held in a public repository**, include URLs, accession numbers or DOIs. If this information will only be available after acceptance, indicate this by ticking the box below. For example: *All XXX files are available from the XXX database (accession number(s) XXX, XXX.*).<br>• If the data are all contained **within the manuscript and/or Supporting Information files**, enter the following: *All relevant data are within the manuscript and its Supporting Information files.*<br>• If neither of these applies but you are able to provide **details of access elsewhere**, with or without limitations, please do so. For example:<br><br>*Data cannot be shared publicly because of [XXX]. Data are available from the XXX Institutional Data Access / Ethics Committee (contact via XXX) for researchers who meet the criteria for*	The material presented in the paper is stored in the Institute of Archaeology and Ethnography of the National Academy of Sciences of the Republic of Armenia and available to study upon request to the authors.

access to confidential data.<br><br>*The data underlying the results presented in the study are available from (include the name of the third party and contact information or URL).*<br>• This text is appropriate if the data are owned by a third party and authors do not have permission to share the data.<br><br>* typeset	
Additional data availability information:	Tick here if the URLs/accession numbers/DOIs will be available only after acceptance of the manuscript for publication so that we can ensure their inclusion before publication.

Powered by Editorial Manager® and ProduXion Manager® from Aries Systems Corporation

Manuscript
Click here to access/download;Manuscript;Malinsky Buller et al Kalavan 2.docx

Short-term occupations at high elevation during the Middle Paleolithic at Kalavan 2 (Republic of Armenia)

Malinsky-Buller A.[1], Glauberman P[2,3], Ollivier V[4,5], Lauer T[6], Timms R[7], Frahm E[8], Brittingham A[9], Triller B[10], Kindler L[1], Knul M. V[11], Krakovsky M[12], Joannin S[13], Hren M.T [14,15], Bellier, O[16,5], Clark, A. M[7], Blockley S. P.E[7], Arakelyan, D[17], Marreiros, J[18,19], Paixao, E[18,19], Calandra I[18], Ghukasyan R[2], Nora D[19], Nir N[20], Adigyozalyan A[2], Haydosyan, H[2], Gasparyan, B[2]

1. MONREPOS, Archaeological Research Centre and Museum for Human Behavioural Evolution, Neuwied, Germany. Schloss Monrepos, Neuwied.
2. Institute of Archaeology and Ethnography, National Academy of Sciences of the Republic of Armenia, Yerevan, Armenia.
3. Xi'an Jiaotong-Liverpool University, Suzhou, China.
4. CNRS, Aix Marseille Univ, Minist Culture, LAMPEA, Aix-en-Provence, France.
5. Aix Marseille Univ, CNRS, FR ECCOREV, Aix-en-Provence, France.
6. Max Planck Institute for Evolutionary Anthropology – Department of Human Evolution, Leipzig, Germany.
7. Centre for Quaternary Research, Department of Geography, Royal Holloway, University of London, Egham, Surrey, UK.
8. Yale Initiative for the Study of Ancient Pyrotechnology, Council on Archaeological Studies, Department of Anthropology, Yale University, New Haven, CT, USA.
9. University of Connecticut, Department of Anthropology, Old World Archaeology, Storrs, CT, USA.
10. Institute of Ancient Studies, Johannes Gutenberg-University, Mainz, Germany.
11. Department of Archaeology, Anthropology and Geography, University of Winchester, Department of Archaeology, Winchester, UK.
12. Israel Antiquities Authority, Jerusalem, Israel.
13. Institut des Sciences de l'Evolution de Montpellier, Université de Montpellier, CNRS, EPHE, Montpellier, France
14. Department of Geoscience, University of Connecticut, Storrs, CT, USA.
15. Department of Chemistry, University of Connecticut, Storrs, CT, USA.
16. Aix Marseille Univ, CNRS, IRD, INRAE, Coll France, CEREGE, Aix-en-Provence, France

17. Institute of Geological Sciences, National Academy of Sciences of the Republic of Armenia, Yerevan, Armenia
18. TraCEr, Laboratory for Traceology and Controlled Experiments at MONREPOS Archaeological Research Centre and Museum for Human Behavioural Evolution, RGZM, Schloss Monrepos, 56567 Neuwied, Germany.
19. ICArEHB – Interdisciplinary Center for Archaeology and Evolution of Human Behaviour, Universidade do Algarve, Portugal
20. Department of Earth Sciences, Physical Geography, Freie Universität Berlin, Berlin, 12249, Germany.

Abstract

The Armenian highlands encompasses rugged and environmentally diverse landscapes and is characterized by a mosaic of distinct ecological niches and large temperature gradients. Strong seasonal fluctuations in resource availability along topographic gradients likely prompted Pleistocene hominin groups to adapt by adjusting their mobility strategies. However, the role that ~~higher~~ elevated landscapes played in hunter-gatherer settlement systems during the Late Pleistocene (Middle Palaeolithic [MP]) remains poorly understood. The MP site of Kalavan 2 (Armenia) is currently the only excavated open-air locality in the Armenian highlands with a stratified sequence that preserves faunal remains and lithic artifacts in association. At 1640 m above sea level, the site is ideally positioned for testing hypotheses involving elevation-dependent seasonal mobility and subsistence strategies. The results of our recent excavations and the analysis of chronologically and environmentally contextualized archaeological data suggest repeated ephemeral occupations, likely related to hunting in a high-elevation steppe landscape

Introduction

Modern states of Armenia, Azerbaijan, and Georgia occupy an area ca. 240,000 km², located between the Greater Caucasus Mountains in the north and the Araxes River in the south (Fig. 1). This area which geographically covers the southern Caucasus and the north-eastern part of the Armenian highlands encompasses a rugged and environmentally diverse landscape, with a mosaic of different ecological niches and a prominent temperature gradient. (Fig. 1). This area encompasses a rugged and environmentally diverse landscape, with a mosaic of different ecological niches and prominent temperature gradients. Strong seasonal fluctuations occur across elevations that range from sea level to more than 4000 m asl [1]. Such a terrain posed challenges – and offered opportunities – for hunter-gatherer populations, especially regarding seasonal resource availability.

Hunter-gatherers' decisions regarding their mobility and settlement strategies took into account the temporal and spatial accessibility of resources as well as social factors [2]. These coping strategies are aimed at mitigating ecological risks, thereby enabling their adaptation to environmental constraints. The archaeological record contains an archive that echoes the past cumulative decisions regarding hunter-gatherers mobility and land-use [3]. Environmental conditions during Marine Isotope Stages (MIS) 4 to 2 (ca. 70–29 ka), indicated by regional long-term climatic records, were characterized by abrupt, millennial-scale temperature and precipitation oscillations between cold (stadial) and warm (interstadial) stages [4–12]. Yet, during this period, there is an abundance of Middle and Upper Paleolithic sites spread across various topographic gradients and a diverse array of biomes [13–25]. The role of mountainous environments in settlement systems during the Late Pleistocene i.e. the Middle Palaeolithic (MP), is however poorly integrated with current understandings of MP settlement patterns and lifeways.

The open-air MP site of Kalavan 2 (Republic of Armenia; Fig. 1) is situated at 1640 m asl and is an ideal locality for testing the role of high-elevation sites within their corresponding settlement systems. The site is located on the northern slopes of the Areguni Mountains, ca. 8 km north of the northern shores of Lake Sevan, and 70 km northeast of Yerevan (Fig. 1). Kalavan 2 is positioned at the confluence of the Barepat River and one of its tributaries. The Barepat is connected to the Kura River watershed by the Getik and Aghstev Rivers (Fig. 2). The site was initially excavated by

an Armenian-French team in 2006–2007 that exposed MP horizons of lithic artifacts and faunal remains distributed in at least 4 m of stratified alluvial deposits [14].

Here we focus on the results of renewed excavations at Kalavan 2, conducted between 2017 and 2019 (the Institute of Archaeology and Ethnography, Republic of Armenia and Monrepos Archaeological Research Centre and Museum for Human Behavioural Evolution). We first describe the model of elevational mobility strategies and its testable implications for the Kalavan 2 record, particularly concerning the timing and duration of occupation. Next, we update the site's stratigraphy and chronology based on new sedimentological, X-Ray Fluorescence Elemental (pXRF) analyses, tephra, and luminescence (pIRIR) dating analyses. We then report our findings based on recently recovered lithic and faunal assemblages. Additionally, two paleovegetation proxies – pollen and leaf waxes – were analyzed to better characterize the environment during MP occupations. Together our results highlight MP hunter-gatherer lifeways, specifically with respect to their subsistence, mobility, and land-use. This record, therefore, enables us to test hypotheses regarding elevational mobility patterns and the nature of hominin-environmental interactions in a mountainous environment.

Testing elevational mobility strategies

The area between the Greater Caucasus Mountains and the Araxes River is a hotspot of biodiversity, hosting a large number of endemic plant and animal species related to its role as a glacial refugium [1,26]. Modern climatic records from Armenia reveal large temperature and precipitation gradients across different elevations and strongly seasonal climates. For example, mean July temperatures range from 19 to 33°C, whereas January temperatures typically span between -4 to -27° C, depending on elevation. Elevational gradients have a strong influence on floral and faunal community distributions [1] Fig. 15.4). During the Late Pleistocene, seasonal fluctuations in temperature and humidity were likely even more pronounced than today [4]. During glacial periods, winters were thought to have lasted longer, while summer droughts were more frequent, whereas during interglacials precipitation occurred mainly in spring to early summer [27–29]. These hydrological changes impacted distributions of terrestrial biota, such as shifts in the treeline elevation [10,11]. The seasonal distribution of resources currently shapes animal migrations

[30–34] and most likely will also then have governed the migrations of animals and the hominins that followed them [35,36].

Mobility is an inherent strategy of population sustainability among hunter-gatherers [2]. It seems as well to be the primary strategy used to locate resources, but social networks are also crucial in providing a "safety net" against fluctuating resource availability [37,38]. Recent models have identified habitat suitability as the best predictor of hunter-gatherer mobility [39]. Thus, decision-making regarding mobility and settlement strategies likely took into account the spatio-temporal accessibility of subsistence and/or social resources [40,41]. Based on the current local conditions in Armenia (e.g., annual precipitation distributions, snow coverage, seasonal animal migration patterns), we postulate that the Late Pleistocene landscape of Armenia consisted of a fairly predictable, yet topographically segregated, distribution of diverse resources over short distances. Consequently, seasonally fluctuating resource availability along topographic gradients in the region most likely prompted Late Pleistocene hominin groups to adapt through adjustments in their mobility strategies.

We can hypothesize that the settlement strategies of Late Pleistocene hunter-gatherer groups were strongly conditioned by these elevation-dependent seasonal temperature and precipitation gradients [35]. If this hypothesis cannot be refuted, we expect:

(i) in winter, higher altitudes being inaccessible, populations would move to lower-elevation areas/enclosed sites, possibly taking advantage of the moderating effects of river valleys and paleo-lake basins;

(ii) in spring and autumn, hominins groups would follow migrating game and sporadically extend their mobility range into higher elevations; and

(iii) during summer and autumn, hominin groups would spend longer periods at higher altitudes.

Archaeological sites at the highest elevations are expected to be occupied mainly during the summer and not in other seasons. Elevation-dependent seasonal hunter-gatherer mobility creates a set of testable expectations displayed in the archaeological proxies related to the roles of sites within the settlement system [35,36,42]. At 1640 m asl, Kalavan 2 is situated within an elevational ecotone of diverse habitats [43], providing an ideal case study for integrating high elevation adaptations within hunter-gatherer`s settlement systems.

Hunter-gatherers organize settlement strategies along a continuum of logistical and residential mobility [37]. We dichotomize between two hypothetical extremes of this continuum: (i) residential sites ("home bases") and (ii) ephemerally occupied, task-specific sites. Residential sites reflect long-term occupations with varied types of activities, whereas task-specific sites refer to short-term occupations with restricted sets of activities. At high elevations during summer, we should expect more ephemeral occupations, specifically task-specific sites that are represented by low-density lithic artifact assemblages exhibiting fragmented core reduction sequences and abundant retouched pieces, highly curated and used for a narrow range of activities (see also [44,45]). During winter, Late Pleistocene hominin settlement and mobility systems should relocate down to lower elevations, creating longer-duration residential sites. Such sites should contain dense palimpsests of lithic artifacts, encompassing a greater proportion of reduction sequences and tools used for diverse activities (see also [45,46]). From a faunal perspective, residential sites should contain diverse hunted assemblages with selective transport of meat and fat-rich body parts. At task-specific sites dedicated to butchery, we expect monospecific faunal assemblages containing the non-nutritional bone elements [47–49]. These lithic and faunal expectations provide a framework for testing our hypothesis and its expectations, as outlined above.

The stratigraphy and depositional environments at Kalavan 2

Initial excavations at Kalavan 2 were conducted in 2006-2007 by an Armenian-French exped[illegible]n. This team opened three trenches within a Late Bronze Age tomb field [50]. The main excavation area – Trench 2 – exposed 7 m^2 to a depth of 99–115 cm below the ground surface. Within this upper portion of the site's deep stratigraphy, eight layers were identified. An additional 260 cm were excavated in a 1 m^2 sounding that exposed twelve additional layers. In total, within the 380 cm thick sequence, 19 stratigraphic layers were identified by the excavators [14]. Layers 19 to 16 are part of an alluvial terrace with major changes in the deposited material and particle size. Layers 15 to 11 were interpreted as a cyclical alluvial sequence with alternating sub-angular gravel and sandy-silt lenses that might correspond to seasonal deposition. Layers 10 to 6 contain a mixture of colluvial/alluvial deposits that have undergone weak pedogenesis. Layers 5 to 3 contain numerous sub-angular to sub-rounded gravels and stones in erosive contact, the product of major hydrological changes that began with torrential slope erosion followed by alluvial deposition. Layer 2 is a silty-

sandy deposit with very small and diffuse sub-rounded gravels that represents a low-energy alluvial phase. Layer 1 corresponds to a humic horizon related to recent edaphic conditions with soil development based on the alteration of Layer 2 [14].

In 2017-2019, new excavations were undertaken by our international research group. The three original trenches were re-opened and expanded, and an additional trench was excavated (Trench 4) (Fig. 3). A total of 2532 lithic artifacts from 13 sedimentary units and 2163 bones were recovered from 15 sedimentary units. Trench 2 was expanded to enlarge the sample of archaeological materials, re-evaluate the sedimentary sequence, and collect samples to refine the chronology. Trenches 1, 2, 3, and 4 (T1–T4 hereafter) were excavated to assess the spatial distribution of the archaeological materials and the lateral changes in the depositional sequence (Fig. 3). A total area of 50 m^2 was excavated (T1 = 17 m^2; T2 = 13 m^2; T3=12 m^2; T4= 8 m^2). All excavated sediments were dry-sieved through 5 mm mesh; a ca. 10% sample was wet sieved. In T4, two sub-squares were sampled for wet sieving over the full sequence (2 m^3 – one metric ton of sediments).

The distances between trenches and the lack of clear stratigraphic continuation necessitated that each trench has its own numbering of sedimentary units (Table 1-4; Fig. 4-7).

In T1, 17 m^2 were excavated to 250 cm below the ~~ground~~ surface (1626.04-1623.64 m asl). The stratigraphy ~~contains~~ seven units, alternating between gravels (1a, 2a, 3a, and 4) and alluvial deposits (1b, 2b, 3b, 5, 6, and 7; for a detailed description of each unit see Table 1; Fig. 4a,b). Faunal remains and lithic artifacts were recovered from Units 1b, 2b, and the interface of Units 5 and 6, while the highest artifact density appears in Unit 1b (Table 6). The thickness of the archaeological horizons in Units 1b and 2b varies between ca. 10 and 20 cm. The lithic and faunal assemblages in Units 5-6 are dispersed over 40-50 cm at the undulating interface of those units. In Unit 1b, the find density is 307 lithic artifacts and 225 bones per m^3, whereas the density in Units 5-6 is much lower, at 34 lithic artifacts and 28 bones per m^3 (Table 6 and 13).

In T2, 13 m^2 were excavated to 100 cm below the ground surface (1629.42-1628.42 m asl). The lower layers exposed in the earlier excavations (Layers 10–20) were not re-exposed during recent excavations. The T2 stratigraphy contains eight sedimentary units. Among them, three phases of gravel were deposited (1a, 2a, and 3a), while two units (4 and 6) are alluvial sediments. Unit 5 is ~~thought to represent~~ a higher colluvial input. Unit 7 has an isolated lens (ca. 10 cm thick) of coarse- to very-

coarse-grained volcanic ash (see Table 2 for more detailed descriptions; Fig. 5). Our observations of the exposed stratigraphy are similar to those of the earlier excavations [14], thereby allowing us to draw correlations (i.e., Unit 4 in the present stratigraphy = Level 7 in the former stratigraphy; Units 5-6 = Levels 5-6; Unit 7 = Level 2) The lithics and faunal assemblages derive from Units 4, 5-6, and 7. The thickness of these archaeological horizons is usually $\lesssim$ 10 cm. Lithic and faunal find densities for these sedimentary units can be found in Tables 6 and 13. The artifact density of layer 7 from previous excavation, as reported in [14] is 145 lithics and 75 bones per m^3. In Units 5 and 6, the density of finds is 20 lithic artifacts and 30 bones per m^3, and in Unit 7, 135 lithic artifacts and 50 bones per m^3 (Tables 6 and 13).

In T3, 12 m^2 was excavated to 140 cm below the ~~ground~~ surface (1628.91-1627.77 m asl). The stratigraphy ~~includes~~ eight sedimentary units that alternate between alluvial deposits (Units 1, 2b, 3b, and 7) and alluvial/ colluvial gravels (2a and 3a) (see Table 3 for a detailed description; Fig. 6). Lithic artifacts were scattered in Units 1 (n=3), 2b (n=16), and 4 (n=21) at an extremely low densities of finds. Fauna remains were only found in Units 2b and 4.

In T4, 8 m^2 was excavated to 400 cm below the ~~ground~~ surface (1631.60-1627.60 m asl). The initial accumulation of the sedimentary sequence is alluvial (Units 1 and 2), with Unit 1 containing an abundance of medium-coarse grained volcanic ash. Units 3-6 reflect successive paleosols with a lens of tephra (Unit 6a) composed of medium-grained ash (see Table 4 for a detailed description; Fig. 7). Only a few lithic artifacts (Units 2, 5, and 6) and macro-faunal remains (Units 2, 5d, 6b, 7, and 8) were found, although microfaunal remains were abundant throughout the sequence.

Geomorphology and sedimentology

Kalavan 2 is located at the confluence of the Barepat River and a tributary locally known as the Dani River. The reconstructed depositional dynamics at Kalavan 2 reflect inputs from an alluvial fan of the Dani catchment and the wider watershed of the Barepat River (Fig. 2). The Barepat River is a third-order stream [51], and its catchment covers 58.8 km^2, descending from 2300 to 1217 m asl over 16 km (maximum slope = 28%; average slope = 7.5%). The Dani River is a second-order stream and its watershed covers 3.3 km^2, descending from 2423 to 1660 m asl over 3.4 km (maximum slope = 49%; average slope = 22%). The expected contribution of the Dani River should manifest as rapid flood events with poor clast sorting, including

larger, more angular clasts mixed with colluvial inflow from the slopes. The Barepat River's expected input would be deposits of smaller grain sizes (due to the wider distal floodplain) that should be better sorted and typically alluvial with much more rounded and/or sub-rounded clasts. The Dani watershed transports three main lithologic types: Cretaceous limestone, Paleogenic sandstone, and various volcanogenic rocks. The Barepat watershed also encompasses conglomerates and metamorphic rocks as well as a few Cretaceous limestones within its upstream basin. Furthermore, the Barepat watershed includes numerous spring and travertine deposits (Fig. 2).

During the Late Pleistocene, Kalavan 2 was located at the end of the Dani watershed at the meeting point of the Dani catchment alluvial fan with the Barepat River, creating alternating braided channels and thalwegs in response to shifting hydroclimatic and meteorological conditions. As attested in the excavation trenches, sedimentary dynamics between these rivers changed over time. The ground surface of the site area overlooks the valley of the Dani River by ca. 8 m above the current stream and ca. 18 m above the current Barepat stream. The Trenches 1-4 lie in different positions relative to the Dani and Barepat rivers. T1 is the closest to the Barepat while T3 is closest to the Dani (Fig. 3). Both T2 and T4 are located between the two, yet the current surface of T4 is 2 m higher in elevation than T2. The distance between T1 and T2 is ca. 25 meters, while T3 and T2 are closer to each other, less than 5 meters apart. The distance between T2 and T4 is ca. 30 meters.

Two methods were used to characterize the sediments. First, portable X-ray fluorescence (pXRF) was used to analyze 43 sediment samples from four trenches (see S2 for the pXRF protocols). Utilizing pXRF, we sought to geochemically differentiate between sediments derived from the Barepat watershed and those from the Dani. Second, micromorphological analysis was conducted on four thin sections from the T1 and T2 stratigraphic levels (also see S2 for the micromorphological methods). These analyses aimed to better understand the site formation processes.

pXRF sediments results

For all trenches, the major detectable elements of sedimentary deposits (Table 5) are Si, Al, Ca, Fe, and K. Light elements (LE; H through Na) are not measured but constitute between 57 and 88% of the samples by mass. Mg is also present but only at detectable concentrations in certain levels exposed sequences.

In T1, the mean LE values are the lowest (64.2%), while Si, Al, and K have the highest mean values (Si = 18%; Al = 6.2%; K = 2.4%). The mean elemental values are

comparable to T1, however, its Fe values are the highest measured in all trenches (μ = 4.0%). In contrast, T3 and T4 are different from T1 and T2, including higher LE mean values (T3 = 85.1% and T4 = 82.4%). T3 has the lowest mean values for the major elements (Si = 4.3%; Al = 1.5%; Ca = 3.4%; K = 0.4%). T4 is very similar to T3 in terms of elemental values, but it does have a higher mean Ca content (4.9%).

To summarize, the pXRF data reflect the spatial relationships of the trenches: the major-element chemistries of T1 and T2 are more similar to each other, whereas T3 and T4 are chemically similar to one another (See S2, Fig. 1). T1 and T2 are closer to the current Barepat riverbed, while T3 and T4 are closer to the Dani (Fig. 2). An elemental signature of the Barepat appears to be higher Mg content. The sources of Mg are most likely spring-derived (see [52] reference therein). Many springs related to local tectonic faulting and fracturation feed into the Barepat River watershed (n = 31 for the Barepat; n = 7 for the Dani River watersheds), likely contributing Mg into the sedimentary input. The lack of Mg in the gravel units (i.e., Units 1a and 3a in T1; Units 1a, 2a, and 3a in T2) supports this interpretation. Those units most likely formed as an outcome of flooding events within the Dani watershed given that the large, angular calcareous blocks could only derive from upstream in the Dani catchment (Fig. 2).

Micromorphological analysis results

Four thin section samples from T1 (n=2) and T2 (n=2) were examined (Sp. 2 Fig. 2). A summary of our findings is presented here, while additional details can be found in the supplementary materials (S2 Table 2 and S2, Fig. 2). All descriptions follow Stoops and Jongerius [53].

T1 Unit 1b (sediment sample MM3; thin sections MM3.1 and MM3.2) exhibits a random distribution with volcanic, metamorphic, and sedimentary subrounded heterogeneous grains in the matrix (Sp. Fig. 2, 3). MM3.1 is principally composed of fluviatile deposits with weak traces of pedogenesis. It also includes micro-fragments of obsidian flakes and bone (Sp. Fig. 2). MM3.2 has a similar fabric and texture as MM3.1 but with Mn, less evidence of pedogenesis, and more colluvial sediments in a fluviatile deposition (Sp. Fig. 3).

T1 Unit 5 (sample MM4) has a random distribution of grains (Sp. Fig. 4). It has a slightly denser grain texture than MM3 and includes more sub-angular to angular material with light rock alteration (i.e., low degree of chemical or mechanical erosion).

Structures in MM4.1 are indicative of fluviatile and colluvial deposits with evidence of bioturbation (Sp. Fig. 4) but without notable pedogenetic development. MM4.2, relative to MM4.1, shows a slightly denser grains texture within the matrix and the appearance of reworked soil elements or aggregates with secondary veins of gypsum (Sp. Fig. 5). Despite some bioturbation traces, the fluviatile component remains dominant in the structure and organization of the sediments (Sp. Fig. 5).

T2 Unit 4 at the contact with Unit 5 (sample MM1) exhibits a lower frequency of grains in the matrix (Sp. Fig. 6,7) than do MM3 and MM4 in T1 (Sp. Fig. 2-5). Grains in MM1 are rounded to sub-rounded and coated by secondary veins of gypsum/calcite (Sp. Fig. 6,7). MM 1.1 indicates an alluvial deposit mixed with light colluvial supply. Little evidence of pedogenesis and bioturbation is visible. Some evidence of sediment compaction indicates short-term top-soil formation (Sp. Fig. 6). MM1.2 contains fewer grains than MM1.1 (Sp. Fig. 6 vs. 7). Gypsum and calcite are also present, as are micro-fragments of sub-angular bones. MM1.1 also indicates an alluvial deposit mixed with colluvial elements, and sediment compaction is visible as well (Sp. Fig. 7).

T2 Unit 5 at the contact with Unit 6 (sample MM2) has a random to clustered basic distribution pattern (Sp. Fig. 8, 9). MM2.1 shows poorly sorted sub-rounded particles and light rotation structures, attesting to chemical and/or mechanical erosion. Such erosion shows grain crushing, fragmentation by ice lensing, grain stacks, and smooth fractures typical of frost microstructures. This thin section is mainly constituted by alluvial deposits affected by preliminary to medium frost action (Sp. Fig. 8). MM2.2 exhibits clustered basic distribution patterns with sandy lenses and sub-rounded particles, and it has reworked sub-angular blocky peds and channel biostructures due to bioturbation. Hence, it reveals an alluvial deposit that reworked soil peds and was affected by a light post-depositional pedogenesis (Sp. Fig. 9).

Most thin sections from T1 and T2 show fluvisols, colluvial soils, or alluvial deposits with poorly evolved pedogenesis. This analysis agrees with the stratigraphic observations, recording the discontinuous geomorphic dynamics of an alluvial fan at the confluence of the Dani and Barepat Rivers during the Late Pleistocene. However, some specific observations can be made. In T2 Unit 4, there is some evidence of compaction, which could correspond to human and/or animal activity (trampling) during a short time on a soft, saturated sediment [54]. In T2 at the contact of Units 5 and 6, frost action reveals short-term weather trends such as a severe winter season. This frost feature appears very light without heavy modification of the sedimentary

structure (no ice-wedge casts or sand wedges, fragipan horizons, flat bottomed cryoturbations, gelifluction deposits, etc.). During short cold events, when flows of water are less uniformly distributed, crystals such as gypsum or carbonates are formed, as it has been observed in the field and under the microscope [55]. This type of crystallization does not appear in all thin sections (e.g., MM4.1, MM1.1, MM1.2). There is ~~not support~~ for a periglacial climate or long-term cold climatic conditions, given the evidence of frost was only preserved in MM1.1 and MM1.2. Thus, the evidence supports short periods of frost similar to the conditions prevailing today in the Kalavan area during winter.

Chronology of the sedimentary sequence

The prior site chronology was based on an analysis of four ^{14}C samples from the 2006-2007 excavations. Their results placed the MP occupation between 42 and 16 ka; however, these ages have since been questioned due to laboratory challenges and stratigraphic inconsistencies (see S1). Our project used two dating methods to establish a new chronological framework for the sedimentary sequences under investigation, and refine the timing of MP occupations. The first method is luminescence dating, which is based on the accumulation of trapped charge in natural dosimeters such as quartz or feldspar minerals. It has the potential to determine the last sunlight exposure and, therefore, the burial ages of sediment grains [56] (see S1 for details). The second method is tephrochronology, the goal of which is to create a relative, comparable chronology that can be linked to the volcanic origins of visible and microscopic ash layers (tephra and cryptotephra, respectively). Our protocols for the visible tephra followed standard sedimentological methods [57], while our crypto-tephra analytical methods followed Lane et al. [58] and Blockley et al. [59]. Specifically, volcanic glass shards extracted from the sedimentary sequence were measured for major and minor elements using electron microprobe analysis (EMPA) [60] (see S1 for the complete methods).

Luminescence chronology

Fifteen post-infrared infrared stimulated luminescence (pIRIR) samples have been analyzed [61,62] on fine-grain (4-11 µm) polyminerals. Fig. 4-7 and Tables 1-4 document the stratigraphic locations of pIRIR sampling in the various trenches. Age estimates from the bottom- and middle parts of the sedimentary sequences (T1 Units

1b-5, T2 Units 1b-4, T3 Units 1-3b, and T4 Units 2-4) range between 59.4 ± 4.5 and 51.3 ± 4.8 ka and overlap within the uncertainty intervals. Hence, the luminescence ages do not allow us to distinguish among several periods of sedimentation, but they point to the formation of the bottom- and middle sedimentary units at ca. 55 ka, during early Marine Isotope Stage (MIS) 3. The latest ages corresponding to the last MP occupations are ca. 45 ka (T1 top of Unit 6 = 46.4 ± 4.1 ka and T2 Unit 7 = 44.1 ± 3.4 ka). All ages are shown in Table 5 and Figs. 4-7.

Tephra results and interpretation

Three visible volcanic ash horizons were identified across the trenches at Kalavan 2. In T2 Unit 7 the tephra is composed of coarse – very coarse-grained ash and forms an isolated lens (ca. 10 cm thick) (T17-0174) (Fig. 5). In T4, two ash layers were identified: a lower tephra composed of medium-coarse grained ash forming a constituent of Unit 1 and (T17-0172); and a second tephra composed of medium-grained ash, and forming Unit 6a (ca. 5 cm thick), (T17-0173) (Fig. 7). Mean analyses from the vitreous phase of this tephra are listed in S1 Table 1, with the complete chemical datasets presented in S1 Fig. 5 and S1 Table 2. Concentrations of cryptotephra shards were detected in almost all of the 10-cm scan samples from T1 and 2 (S1 Fig. 6.). In T1, two distinct zones of glass shards were identified: a lower one at 2.56-2.86 m and an upper one at 2.24-2.50 m below ground surface. Refining the most concentrated samples from the zones revealed two peaks in shard concentration: a lower one at 2.62-2.64 m (T18-0098; Unit 1b; 1379 shards/g) and an upper one at 2.32-2.34 m below ground surface (T18-0095; Unit 3b; 2912 shards/g). In T2, glass shards were detected in every scan sample between 0.06-1.04 m below the ground surface; however, a greater concentration was found between 0.46-0.66 m below the ground surface (S1 Fig. 6). This interval was refined to a 2-cm resolution, which highlighted two peaks in glass shard concentration: a lower one at 0.64-0.66 m (T18-0110; Unit 6; 1839 shards/g) and an upper one at 0.54-0.56 m below ground surface (T18-0105; Unit 7; 2146 shards/g) (S1 Fig. 6).

Chemical interpretation

Data from the analysis of the vitreous phase of these tephra are presented in S1 Fig. 5 and S1 Table 2.

The potential source regions for volcanic material deposited at Kalavan 2 and over the Armenian highlands is particularly wide-ranging. Local volcanic centers are numerous (see [63–65]), and dominant atmospheric circulation patterns place the

Armenian highlands downwind of Mediterranean, Anatolian, and Carpathian sources, as well as in the likely dispersal envelope of volcanoes from the Greater Caucasus [1,66].

Two of the three visible tephra (T17-0172 and T17-0174) have a mildly alkaline trachyandesite geochemical signature, whereas the third is mildly alkaline trachyte/ trachydacite (T17-0173). Their compositions and visibility in the trench suggest that they originate from a relatively proximal volcanic source. During the Late Pleistocene, centers in the Gegham Highlands are known to have produced lavas with an alkaline trachyandesitic composition [63–65], so it is possible that these eruptions (ca. < 50 ka) generated plumes of ash that dispersed over the short distance across Lake Sevan to the site (ca. 40-60 km). Unfortunately, at present, the lack of grain-specific elemental data and detailed tephrostratigraphies from these volcanic centers in the Gegham Highlands limit the ability to support or refute such a hypothesis. Other potential source candidates are volcanoes located in the Eastern Anatolian Volcanic Province (EAVP), which produced trachyandesites and trachydacites throughout the Pleistocene [67–70]. One possible correlation is the Çekmece Formation (S1 Fig. 7): a series of eruptions thought to originate from Nemrut volcano near Lake Van [71,72]. The estimated age of this series is debated, but it is thought to have occurred sometime ca. 60-30 ka [72]. Such a broad age estimate reflects the current stratigraphic uncertainty of this volcanic series, but the estimate overlaps well with the pIRIR dates at Kalavan 2.

Several cryptotephra populations exhibit a distinct peralkaline rhyolite signature (T18-0098_a; T18-0095_a; T18-0110_a; T18-0106_a; T18-0105_a,b; (S1 Fig. 5; S1 Table 1). In the local-medial environment, one prominent volcano is known to have yielded tephras of this composition: Nemrut in the EAVP. This volcano has been particularly active during the last ca. 200 ka, producing several large eruptions (VEI≥5), including the HP-10/AP-6 eruption (ca. 61.6 ka), Çekmece Formation (ca. 60-30 ka), Tatvan Ignimbrite (ca. 45 ka) and Nemrut Formation (ca. 30 ka) [47–51]. Establishing if the cryptotephra at Kalavan 2 relates to any of these large-scale eruptions is difficult at present due to a lack of chemical data for the vitreous phase of Nemrut eruptions and the ongoing chrono-stratigraphic refinement of these events (see [72]). Nevertheless, based on peralkalinity, a tentative link between Nemrut and the Kalavan peralkaline cryptotephras can be proposed, but an exact match remains elusive.

Three of the cryptotephras (T18-0098_b, T18-0095_b, T18-0110_b) are calc-alkaline rhyolite with chemical signatures very similar to tephras from local-medial volcanic centers in Armenia [63,65] and the EAVP [67,70–79] as well as more distant volcanoes from the Aeolian Islands (ca. 2500 km east of Kalavan) [80], the East Carpathians (ca. 1700 km northwest of the site) [81–84], and the Central Anatolian Volcanic Province (CAVP; ca. 940-860 km east of Kalavan) [66,85–90]. At present, it is not possible to provide a more reliable conclusion regarding the provenance of the Kalavan 2 calc-alkaline cryptotephras.

Lithic artifact assemblages

During the previous excavations in T2, lithic artifact assemblages were recovered from five layers: Layers 2, 3, 6, and 7 over the entire 7 m^2 excavation area and layer 11, which was exposed only in a deep test pit [14]. The lithics from Layers 1 and 2 were a mix of Medieval, Late Bronze Age, Chalcolithic ceramics, and MP artifacts, whereas lithic artifacts from Layers 3, 6, and 7 were attributed solely to the MP. Among the MP artifacts, the dominant raw material is obsidian (ca. 59% in Layer 7, for example). Sedimentary rocks including silicified limestone, chert, flint, and jasper as well as very few volcanic rocks such as basalt are also present [12, Table 1]. The authors stressed the dominance of the recurrent convergent unipolar Levallois flaking method and laminar production. Retouched pieces were classified mainly as side-scrapers and retouched Levallois points [14].

In the current excavations, lithic artifacts were found in all trenches in 13 sedimentary units (Tables 7-12). Artifacts from T1 Unit 1b, 2b, and T2 Unit 4 and 7 are vertically dispersed over ca. 10 to 20 cm. The few artifacts from T4 and T3 are vertically dispersed over 40-50 cm thick sedimentary units. In T3, only two artifacts were recovered in Unit 1, 16 in Unit 2b, and 21 in Unit 4 (Table 11). Similarly, low numbers of artifacts were found in T4 Unit 2 (nine artifacts, of which five are < 2 cm), Unit 5 (53 artifacts; 39 of them < 2 cm), and Units 6 and 7 (five artifacts each) (Table 12). The low densities of small, dispersed artifacts in T4 and T3 mainly represent episodic use, maintenance, and discard events. In T1, the lithic artifacts were dispersed in ca. 40-50 cm at the undulating interface of Units 5-6. The artifact density is relatively low: 34 artifacts/m^3 (Fig. 4; Tables 6-7). Lithic artifacts retain well-preserved dorsal scars and edges, and only minor apparent post-depositional alterations in all assemblages, except for artifacts from T1 at the Unit 5 - 6 interface. The edges of the artifacts from

this interface are slightly more abraded, suggesting that they were more likely reworked and in secondary depositional context. All assemblages display MP techno-typological affinities and a low frequency of Levallois flaking, consistent with previous observations [14]. The only later intrusions at the site stem from Late Bronze Age burials that cut into the Paleolithic layers. No diagnostic artifacts indicating later prehistoric periods were found.

The recovered lithic artifacts were made from seven materials: obsidian, basalt, dacite, welded tuff, chert, limestone, and an unidentified metamorphic rock. Here we distinguish between obsidian and the rest of the materials (i.e., non-obsidian). Obsidian sourcing was conducted using pXRF (see below). Sourcing the non-obsidian materials is still in the initial stages and, as such, will not be detailed here. The frequencies of raw material types vary unit-by-unit among excavated assemblages (Tables 7-12).

Obsidian is the most common material with abundances ranging from 55% to 82% of the artifacts. A notable exception occurs in T2, Unit 4, where obsidian artifacts are much less common: 23.5% (Tables 7-12). This differs from the results of past excavations in the same layer, where obsidian constituted 59% of the artifacts [14: Table 1].

Within the obsidian component in all units, there are no indications of the first stages of core preparation and reduction, as there is a small number of cores and core trimming elements (CTEs) (Tables 7-12). Obsidian debitage consists mainly of flakes < 2 cm (up to 75% of artifacts in the assemblages; Tables 7-12). In the earlier excavations, flakes < 2 cm amounted to 61.7% of the obsidian component in T2 Layer 7 (Unit 4 in our new stratigraphic scheme) [14]. Among the obsidian retouched pieces, there is an extremely narrow range of tool types, and the majority can be classified as retouched points ([91]; see also [14], Fig. 9, 1-7, and also fig, 8: 6,7).

Several technological features indicate a range of flaking practices associated with retouched obsidian pieces. "Shaping flakes" is an inclusive term used here to define all by-products of tool modification. Their identification is based on the presence of retouch scars on their dorsal faces. Retouch negatives are defined by their small size (< 0.5 cm), regularity in shape and distribution, and intrusiveness (Fig. 8: 2-3). Shaping flakes remove the previous retouch located on either the proximal edge (Fig. 8: 2-3) or lateral edges (Fig. 8: 1, 5). Most shaping flakes are < 2 cm in maximal dimension. Some of the shaping flakes were removed using soft-hammer percussion

(antler or soft-stone [92] Figs. 2-3), as many of them have diffuse bulbs of percussion (Fig. 8: 2-5). The suggested use of soft hammer percussion on the shaping flakes differs from that observed on most of the obsidian flakes, which exhibit characteristics of hard hammer percussion[92] Figs. 2-3), such as prominent bulbs of percussion ([14], Fig. 8: 6).

Another shaping feature is the removal of the tips from retouched convergent pieces. Some of the removed tips were removed from the direction of a side blow (Fig. 8: 4-5). A unique shaping flake from T2 Unit 7 removed the distal part of a retouched point (Fig 8: 1). Despite constant modification via retouching and removals of tips, the tool typology (i.e., a prevalence of pointed retouched pieces) appears not to have changed through cycles of removals and rejuvenation until discard.

The non-obsidian component exhibits greater indications of core reduction as attested by higher frequencies of cores, CTEs, and hammerstones. Moreover, there are also few indications of initial phases of core reduction, as indicated by a few cores (Fig. 9: 4) and primary elements (flakes with >50% cortex, Fig. 9: 2), (Tables 7-11). The few cores at the site were mostly made on flakes, while some were made on split cobbles (Fig. 9: 4). Non-obsidian assemblages are characterized by flakes, Kombewa flakes, and Levallois flakes (Tables 7-10; Fig. 9: 2-3). Levallois points appear in lower frequencies, for example, in Unit 1 in T1 (Fig. 9: 3). In T2, Unit 4, sixteen blades (Fig. 9: 1) comprise 4.4% of the assemblage, similar to findings from the prior excavations [14] (Fig. 4, 8-7, 18). Refitted blades were also found [14] (Fig. 4, 15). In contrast to the obsidian assemblages, the frequency of the < 2 cm objects among the non-obsidian artifacts in all assemblages is lower and never exceeds 50% (Tables 7-12).

In brief, the behavioral signal in all units indicates high intensities of obsidian tool reduction with repeated cycles of shaping and rejuvenation, while the non-obsidian component suggests core reduction and blank production occurred on-site.

Traceology

A pilot study considered the preservation of use-wear on Kalavan 2 artifacts and the feasibility of its identification. Twenty-two obsidian and two non-obsidian artifacts were studied from T1 Unit 1b, while twelve obsidian and eight non-obsidian artifacts were studied from T2 Unit 4. This sample included the most representative techno-typological categories, including obsidian flakes (n = 2), smaller than 2 cm pieces (21), shaping flakes (5), and tools (6), as well as non-obsidian flakes (8) and cores (2) (see S3 for the protocols and results of the analysis).

Obsidian surfaces and edges, due to the brittle properties of the raw material, are more prone to post-depositional alteration compared to non-obsidian materials. The non-obsidian artifacts, though, did not appear to preserve any microwear traces except for one core on split pebble. Macroscopic traces of percussive activities were identified on this chert core on split pebble (Fig. 9: 4). Among the analyzed obsidian artifacts, nine out of 34 presented specific distributions and orientations of striations and wear polish, indicative of use. Such microwear traces were observed on obsidian scrapers, points, and shaping flakes. Previous research [93,94] suggests that such orientations, distributions, and types of polish and/or striations on scrapers can be related to hard animal material (Fig. 10: a, b). On retouched points and shaping flakes, the use-wear patterns point to woodworking activities [93,94](Fig. 10: c,d). These preliminary findings hint that the shaping flakes and the point fragments may reflect continuous rejuvenation during activities performed on worked materials and motions related to woodworking. This proposal, though, must still be further tested via detailed techno-typological, use-wear, and refit analyses on larger assemblages.

In T2, Unit 4, a group of four stone objects, unique in raw material among lithic artifacts (limestone and dacite) and sizes in comparison to the bulk lithic assemblage, were found in close spatial association near a concentration of broken bones (Fig. 11; see SP3). A preliminary analysis shows the presence of damaged areas characterized by surface crushing/fracturing and abrasive traces (i.e., striations, scratching), which are reflected in abrupt alterations on the surface topography (Fig. 11). The location and distribution of these alterations suggest that the rocks could have been used as anvils for percussive activities. Prior use-wear and residue studies, as well as experimental work on limestone and dacite, have linked pitted anvils to plant processing [95,96], bone processing [97–100], or bipolar lithic production [101]. Since there is no evidence for bipolar production in the lithic assemblages, most likely the pitted stones possible function was for other purposes, such as processing of vegetal materials and/or faunal resources, which will be tested in the future. Further microscopic analyses supported by experiments using materials with similar features are possible avenues for better understanding the function of these artifacts.

Obsidian sourcing

Geochemically matching obsidian artifacts to their volcanic origins across the Armenian highlands offers a means to reconstruct the mobility of the site's occupants.

We analyzed 928 obsidian artifacts following our published pXRF protocols [102–104]. The identified obsidian sources principally reflect two mountainous areas of central Armenia (Fig. 12; S3, Fig. 1) – the Tsaghkunyats (Kamakar, Ttvakar, and Damlik) and Gutansar, Hatis, and Gegham volcanic range (Geghasar)– followed by sources in the Syunik area of southern Armenia (Satanakar and Sevkar). Artifacts from more distant obsidian sources in western Armenia (Pokr Arteni), eastern Turkey (Digor 1 in the Kars region), and southern Georgia (Chikiani) are also present within the analyzed sample. Fig. 13 breaks down the major source areas in each trench by stratigraphy to highlight diachronic trends, such as, for example, the different proportions of source areas in T2 Units 4 and 7.

These results allow us to largely circumscribe the movements of the Kalavan 2 visitors to what is now central Armenia, in particular the vicinity of the Hrazdan River, which acts as an interface between the Gegham and Tsaghkunyats ranges. Gutansar, Hatis, and the three Tsaghkunyats sources are all ca. 50-60 km linearly from Kalavan 2; however, these distances increase to ≥ 120-140 km on foot based on topography. The Syunik sources and Pokr Arteni are 120-130 km from the site linearly, but each of these distances is ≥ 200 km along the most direct routes. The two farthest sources are Chikiani in Georgia (150 km linearly, ≥ 240 km on foot) and Kars-Digor 1 in Turkey (160 km linearly, ≥ 200 km on foot).

Fig. 14 breaks down the sourcing results by lithic artifact class for all four trenches. For simplicity, obsidian sources are grouped by their source areas (e.g., Kamakar, Ttvakar, and Damlik as the Tsaghkunyats sources). Most types in each of the sedimentary units are, as anticipated from our overall dataset and especially Gutansar, Hatis, and Gegham volcanic range (Geghasar). Based on the data in Fig. 14, it would be expected that, for any particular lithic class, Geghasar, as well as Gutansar and Hatis obsidians should be most abundant, followed by Tsaghkunyats obsidians and, finally, Syunik obsidians. Most artifact classes indeed fit such a pattern. An exception is a Levallois flake from the Sevkar source in the Syunik area. Bladelets are another exception: the numbers of Geghasar, Gutansar, Hatis, and Tsaghkunyats bladelets are equal, but the sample is small. Obsidian from the Geghasar, Gutansar, and Hatis sources is equally high in knapping quality as obsidian from the Tsaghkunyats sources. Thus, we do not interpret such an observation as a stronger preference for Tsaghkunyats obsidian for bladelets relative to other lithic classes. Instead, it is likely to be a reflection of mobility patterns coupled with spatiotemporally

distributed production and discard of different tools as needed. Three distant obsidian sources are each represented by only one (Pokr Arteni and Kars-Digor 1) or two (Chikiani) smaller than 2 cm pieces and there is also a single tool made from Pokr Arteni obsidian and another one made from Chikiani obsidian.

Faunal remains

The faunal assemblage from the prior excavations consists mainly of bovid (cf. *Bos primigenius*), horse (*Equus caballus*), wild goat/ibex (*Capra* sp.), and red deer (*Cervus elaphus*). The anthropogenic origin of this assemblage was suggested by the presence of cut marks on a small number of bones and a few impact fractures on bovid bones [14]. Our excavations yielded > 2163 bone specimens (i.e., number of specimens [NSP], following Lyman [105]: Table 13). The bones were cleaned in the field, and a basic taxonomic/taphonomic analysis was performed (see S4 for methods). Faunal remains were excavated in all trenches and retrieved from 15 units (Table 13). The two richest faunal assemblages derive from T2 Unit 4 (330/m^3; 39.5% of the entire NSP) and T1 Unit 1b (225/m^3; 35.3%). The bones are highly fractured long bones (Table 14) and display various stages of weathering: outer surfaces are often heavily affected by root etching, decalcification, and carbonate coating. Taxonomic and bone element identifications, as well as preserved modifications from the early stages of the taphonomic sequence, are limited. Thus, anthropogenic modifications are scarce. No carnivore gnawing was observed, while a minor frequency of rodent gnawing could be identified (T1 Unit 5: n = 2; T1 Unit 3a: n = 1; T1 Unit 1b: n = 3; T2 Unit 4: n = 3).

To date, 79 bones have been ascribed to specific taxa (Table 15). Among the larger ungulates, *Bos/Bison* and a horse (*Equus* sp.) are most prevalent. Three molars belong to *Equus hemionus*, indicating the presence of an additional smaller Equid among the bones. Ten bones, including an antler fragment, belong to *Cervus elaphus*, and nine bones to the subfamily Caprinae (*Capra* and/or *Ovis*). Due to the limited taxonomic identification, bones were attributed to size classes (i.e., categorizing the different life weights of different species; see S4 Table 1 [106,107]). Small cervids (roe deer), goat, and sheep with a weight range of 20–120 kg are attributed to size class 2. Size class 3 represents a range of 120–260 kg of medium ungulates (e.g., red deer, reindeer, fallow deer, wild ass, boar). The size class 4 category includes the larger horses and *Bos/Bison* (ca. 300–1000 kg). Thus, 677 additional bones could be assigned to a size class based on bone robusticity, compacta thickness, and

morphology (Table 16). The results confirm the predominance of larger species (*Bos/Bison* and *Equus* sp.) in all faunal assemblages.

Cut marks can be observed on at least three long bone fragments with almost unaltered bone surfaces from T2 Unit 4 (Fig. 15). In addition, ten bones with possible cutmarks were found in T1 Unit 1b. Long bone shafts with hammerstone-induced fractures were found in T2 Units 4 and 5 and T1 Unit 1b (Fig. 16). Furthermore, eight bones with possible impacts, as well as a few bones that demonstrate color changes potentially related to burning and/or staining [108,109], were found. In T1, such bones were retrieved from Units 5 (n = 4), 2b (n = 1), and 1b (n = 12). Some were calcined, similar to observations made by the prior excavators where they interpreted seven surface modifications as traces of burning [14]. Those bones suspected of having been burnt will be investigated using Fourier Transform infrared spectrometry (FTIR) as a means to either validate or refute hypotheses of thermal alteration [110].

Following the protocols of Sánchez-Hernández [111,112], we sought to discern the seasons at which the animals died by combining meso- and microwear analyses. For animals with seasonal diets, mesowear analyses show an average-diet signal, and the microwear signal corresponds to the seasonal diet. For example, in a given layer, if the animals died in different seasons (e.g., a long-term occupation site), the microwear signal would encompass the full dietary range, and microwear and mesowear analyses would converge. But if all animals died in the same season, be it in the same year or over several different years (e.g., a short-term seasonal hunting site), the microwear signal would correspond to this particular season only, and signals from microwear and mesowear would instead diverge. Nine teeth have been recovered from three trenches (*Bos/Bison*: n = 4; *Equus hemionus*: 3; *Cervus elaphus*: 1; *Equus* sp.: 1), and only five are in suitable condition for wear analysis. Just three individual teeth can be studied using both wear methods (see S3 Table 4), so the current sample size is not sufficient to produce meaningful results based on these methods.

Micro-faunal remains

The micro-faunal analysis (see S4 for detailed methods) is based on NISP and the minimum number of individuals (MNI) obtained from 138 molars (Table 17). NISP and MNI include all identifiable dental elements, including upper and lower molars. For MNI, the most abundant single element is used (e.g., all lower-left m1, all upper-right M1). The species at the site are dominated by the common hamster (*Cricetus*

cricetus) and also includes the Turkish hamster (*Mesocricetus brandti*), molevole (*Ellobius lutescens*), European watervole (*Arvicola terrestris*), common or field vole (*Microtus arvalis/agrestis*), and single occurrences of the common shrew (*Sorex* cf. *araneus)* and marmot (*Marmota* sp.*)*.

In T4, a large number of post-cranial elements are attributed to the common hamster, and the high rate of completeness, lack of digestive marks, and grouping of skeletal elements suggests a non-predatory driven assemblage (following [113]). The presence of unfused bones in the distal femur, proximal tibia, and distal humerus (S4 Table 3) indicate the presence of juvenile hamsters (6-12 months old) [114]. Thus, post-cranial elements in T4 suggest that the common hamster may have been accumulated via fossorial death during winter months. T1 to T3, in contrast, are mainly predator assemblages based on prevalent digestive corrosion and breakage (except for elements from the common hamster). Thus, it is assumed that the small mammals are contemporaneous with their respective units, although no changes are observed in faunal composition among units (Table 17).

Paleovegetation proxies

Two proxies were analyzed to reconstruct the environment during site occupations. The first is identifying pollen preserved in the sediments, and the second is analyzing leaf wax-derived lipid biomarkers. Among wax lipids, *n*-alkanes have become popular leaf-derived biomarkers due to their relatively high persistence against degradation (i.e., insolubility in water, chemical inertness) and their potential to serve as molecular proxies for paleovegetation and paleoclimate reconstruction.

Pollen

The studied sediment samples from all trenches mostly contained 150 pollen grains or fewer. Only two samples from T4 Unit 5 had more than 150 grains (S2 Table 1). The results from T1, T2, and T3 suggest either no preservation of pollen or biased preservation dominated by *Asteraceae Chicoroideae* (T2 Unit 6, T4 Unit 7, T4 Unit 5c, T4 Unit 5b). The pollen from *Chicoroideae* is one of the known resistant pollen types with *Tilia*, *Brassicaceae*, *Caryophyllaceae*, *Asteraceae* that persists despite unfavorable preservation conditions [115]. In the two counted T4 samples, the proportion of *Asteraceae Chicoroideae* is lower (14.8 and 62.5%) than in the previously mentioned samples. The amount of tree pollen is not significant, while the steppic taxa of Amaranthaceae, Centaurea, and Artemisia are more prevalent (S2

Table 1). The depositional conditions of T1, T2, and T3 differ from those of T4. At T4, possible successive paleosols accumulation provided less oxic conditions that enabled pollen grain preservation; however, the studied pollen from T4 is most likely modified from the original assemblage due to post-depositional processes.

Leaf wax (*n*-alkanes) analysis

In the Armenian highlands and the southern Caucasus, the distribution of *n*-alkanes has been demonstrated to differentiate between soils formed under deciduous vegetation and grasses [10,116]. Soils under grasses typically have higher average chain length (ACL) values (~30), and the most abundant *n*-alkane is nC_{31}. Soils under deciduous trees have lower ACL values (~29), and the most abundant n-alkane is nC_{29}. Grassy soils within the Caucasus also typically have nC_{27}/nC_{33} ratios <1, whereas deciduous soils have nC_{27}/nC_{33} ratios >1. There is no significant difference in a metric known as odd-over-even predominance (OEP) between deciduous (8.6) and grassy (8.4) soils. Such high OEP values, though, indicate that modern soils in the Southern Caucasus have not undergone significant bacterial or thermal alteration, which tends to lower OEP values.

Seventeen samples were taken from 17 sedimentary units in T1-T3 (see S2 and Table 18 for method and sampling). The distribution of *n*-alkanes shows fluctuating environmental conditions during sedimentation and occupation of the site (Table 18). High OEP values throughout the entire sequence indicate little to no post-depositional alteration of the *n*-alkanes, suggesting a well-preserved environmental signal (S2 Fig. 10). Most samples align with present-day grassy landscapes or indicate a mix of grass and deciduous sources of *n*-alkanes (S2 Fig. 11), as the concentration of nC_{31} and nC_{33} is typically higher than nC_{27} and nC_{29}). In the units dated ca. 50-60 ka, most samples either overlap with present-day grass soils or fall between the disruption of grass and deciduous soils with one exception (T2 Unit 2b), which has a low ACL value that overlaps with present-day deciduous soils (S2 Fig. 11).

Discussion

Our data elucidate three aspects regarding Kalavan 2 behavior and their environment background. The first aspect permits us to contextualize the site within its environmental contexts and the formation of the archaeological horizons. The second aspect would test the seasonal occupational patterns hypothesized across the

region's topographic gradients. The third pertains to the activities and intensities of site occupation and position the site within the late MP regional settlement dynamics.

Depositional environments and site formation

During our excavations, trenches exposed two parts of the landscape. In T1 to T3, the sedimentary sequence is a product of the smaller Dani catchment meeting the larger Barepat River catchment. At this confluence, alluvial fans stemming from Dani catchment created smaller-scale braided channels. For example, those channels were documented in T1, where the main channel width was ca. 3 m. Flooding from the Dani catchment most likely represents episodic snow and rain melt overflowing from its mountainous catchment area, whereas the Barepat River distributed finer-grain sediments.

The T4 sedimentary sequence differs from those in T1, T2, and T3 in that no major gravel unit occurs in T4. The T4 deposits regularly display a medium- to low-/very-low-energy alluvial dynamic or surface runoff, reworking colluvial-alluvial inputs of endogenous rocks from nearby outcrops. The succession of paleosols indicates that the location of T4 was a more stable section of the landscape.

The T1-T3 sedimentary sequences represent short-term deposition and erosion events at the interface of the Dani and Barepat watersheds. The Barepat watershed is larger, while the Dani watershed is smaller and steeper (Fig. 2). Sediments from the Barepat are generally finer than those from the Dani, echoing different depositional energies. In the slopes along the course of the Barepat, we have observed numerous springs, the waters from which are likely sources of magnesium (Mg). Such springs occur in much lower frequencies along the course of Dani. Therefore, higher Mg levels can be considered a characteristic of the Barepat sediment supply. Our pXRF analyses of the excavated sediments reflect the watershed sources in the different trenches. Within the sedimentary sequences of our trenches, we attribute the more gravelly/stony sediments and those without Mg in T1 (Units 1a, 3a) and T2 (Units 1a, 2a, 3a) to the Dani. In some cases, given the confluence context, gravelly deposits are a result of simultaneous inflows from both rivers.

Micromorphological analyses from T1 Unit 1b and T2 Unit 4 indicate weak pedogenesis during periods of morphogenic stability. Lateral migrations of braided channels, as manifested in the gravely units of T1 to T3, reflect short-term deposition events. These dynamics probably indicate that the periods of stasis were short and did

not allow complete development of soils. The intricate contacts between the deposits from Dani catchment with fine-grained deposits of the Barepat impede a more refined correlation of sedimentary units among the excavation trenches.

The chronology of the T1-T3 ~~accumulations~~, based on the pIRIR dates, indicates sediment depositional ages of ca. 60-45 ka, that is, at the end of MIS 4 and beginning of MIS 3. T4 Units 1–4 are penecontemporaneous with the formation of the alluvial fans of T1-T3. The paleosol sequence of Units 5a-5d in T4, (ca. 51-36 ka) overlaps with the T1-T3 sedimentary sequence but also accumulated slightly later.

Reconstructing the environments during hominin occupations is complex. For example, paleovegetation proxies differ in spatial resolution. Pollen reflects the wider region since pollen grains are transported by wind, while leaf wax remains represent accumulation within the watersheds of the Dani and/or Barepat Rivers. Pollen from the T4 deposits (51-36 ka) suggests an open landscape with a forest at lower altitudes, unlike the current forested environment of Kalavan today. Due to the preservation and taphonomic limitations, palynological data from T4 was unable to offer unequivocal support for a treeless landscape. Analyses of *n*-alkanes (nC_{25}-nC_{35}) from T1-T3 shows that most samples reflect grassy landscapes or a mix of deciduous and grassy, with one exception (T2 Unit 2b), which is similar to modern deciduous soils. Future chain length and isotopic analysis of *n*-alkanes (nC_{25}-nC_{35}) from T4, in comparison with those from T1-T3, will help to test the suggested environmental reconstruction of these two portions of this landscape. Additionally, sampling of present-day soil and stream sediments along the Dani catchment can yield a framework for a detailed comparison of the present-day sediments with those sampled from the trenches [10,116]. The results of these analyses will enable a more nuanced examination of the *n*-alkane variations along topographic gradients in the landscape surrounding Kalavan 2 [117].

The faunal assemblages include a diverse species of ungulate fauna, such as *Bos*/*Bison*, *Equus* sp., *Equus hemionus, Cervus elaphus*, and *Capra*/*Ovis*. Bovids (*Bos primigenius* or *Bison*) and red deer (*Cervus elaphus*) are quite tolerant of a range of micro-habitats. Caprinae is more prevalent in mountainous and rocky habitats. Horse and onager prefer open steppes and grassland, and the onager is adapted to rather dry conditions [32,118]. Such a diversity of habitats may reflect seasonal ecological conditions where specific habitats of different species overlapped at Kalavan 2. Alternatively, the faunal assemblage may be biased by hominin hunting behaviors [119] (see below). In T4, the few faunal remains that could be identified, including

those that could be assigned to size class, mainly represent red deer (*Cervus elaphus*) and goat/ibex (*Capra* sp.), while the large size classes of *Bos*/*Bison* are absent (Tables 13 and 14).

Small mammal bones are dominated by the common hamster, followed by the Turkish hamster, European ~~watervole~~, common/field vole, and single occurrences of the common shrew and marmot. T1-T4 reflects different accumulation ~~agencies~~ and, in turn, interpretations regarding timing and environment. Abundant common hamster remains in T4 indicate a non-predatory assemblage accumulated by fossorial winter deaths but may be intrusive among units. In contrast, remains in T1 and T2 represent predator-accumulated assemblages and, therefore, reflect deposition during the time of the sediment accumulation. Intriguingly, despite the manner of accumulation differences, the spectrum of microfaunal species is similar among all trenches.

Today the common hamster is extinct in Armenia, but relict populations exist in southern Russia [120]. At the nearby Hovk 1 cave site (2000 m asl), where predator-accumulated assemblages were unearthed, common hamster remains were found in levels from MIS 5 to 4/3. [121]. The European watervole and vole (either common or the field) are absent from Hovk 1 [121] and the Upper Paleolithic (UP) cave site of Aghitu 3 [15]. The common hamster, Turkish hamster, marmot, mole vole, and common vole tend to inhabit steppic or grassland environments [122–126]. The water vole occurs near streams, while the field vole and shrew are associated with moist meadows and forest steppes [127–129]. This taxa combination indicates an open environment with a water body nearby, as supported by our other environmental proxies. Further in-depth study of the common hamsters at Kalavan 2 will also include direct radiocarbon dating to refine their history of accumulation and site formation processes in general.

Regional MIS 3 climatic records are characterized by abrupt temperature and precipitation oscillations over millennia, indicative of changes between cold (stadial) and warm (interstadial) stages [4–9]. During stadial periods, the winters are thought to have been longer and summer droughts more frequent, while there was increased spring and early summer precipitation during interstadial periods [27–29]. Ollivier [130] suggested that these seasonal conditions during interstadials provided greater runoff and sedimentary transfers, which supplied torrential geomorphic components

(e.g., pebbely deposits and developed alluvial fans) observed within the Kalavan 2 trenches.

Between 60 and 45 ka, the Kalavan 2 inhabitants repeatedly occupied alluvial parts of the local landscape (i.e., T1 and T2). The formation processes that preserve or distort archeological horizons are also an outcome of similar landscape geomorphological processes [e.g. 129]. The highest density archaeological deposits were stratigraphically confined within a relatively thin horizon ca. 10-20 cm thick. Some horizons are confined laterally as well (e.g., the archaeological horizons in T2 do not continue into T3). Moreover, lateral changes within a single horizon can be suggested by the differences in density of finds (both lithic and fauna) as well as the relative frequency of the different raw materials frequencies as suggested in T2 Unit 4 and the previous excavations of the same horizon. These spatial variations as seen in the different trenches suggest an anthropogenic non-random distribution of both lithic and faunal remains (Table 6 and 13). Only a few horizons suggest a secondary redistribution of finds (e.g., the wavy contact at the interface of T1 Units 5 and 6, with artifacts vertically distributed over ca. 50 cm).

Fauna from T1 Unit 1b and T2 Unit 4 are characterized by high fragmentation, resulting in limited bone element identification. The few anthropogenic modifications of fauna were found in these two units, in which long bone shafts dominate (Table 14). Such a pattern can be explained by three possible scenarios: (1) poor syn- and post-depositional conditions for bone perseveration [132,133]; (2) bone accumulation as a result of a high hydro-dynamic input [133,134]; and (3) selective transport of animal elements by hominins [135]. Micromorphological analyses, however, reveal the initial stages of stabilization and no indications of hydrodynamic sorting. Moreover, lithics < 2 cm in maximal dimensions occur in high frequencies (i.e., T1 Unit 1b: 85.1% of the obsidian, 46.7% of the non-obsidian assemblages; T2 Unit 4: 57.4% of the obsidian, 42.4% of non-obsidian assemblages). In experimental core reduction and tool production assemblages, this size class comprises ca. 60-80% of lithic artifacts [136,137]. Given that lithics < 2 cm are more susceptible to displacement than larger artifacts [136–138], the high frequencies of small artifacts points to a lack of ~~hydraulic~~ size-sorting. Altogether, our datasets suggest that the hydro-dynamic sorting of bone elements is unlikely. Instead, they suggest that these two faunal assemblages are a product of hominin activities and provide an opportunity to study such behaviors with high fidelity.

Articulating Kalavan-2 within late MP regional settlement patterns

Kalavan 2 preserves evidence of repeated occupation by MP hunter-gatherers ca. 60-45 ka. The site's location at 1640 m asl and its dynamic geomorphological setting suggest that this site was prone to more extreme seasonal changes during the Late Pleistocene than today [27–29]. In this paper, we aimed to test hypotheses involving elevational mobility at Kalavan 2, yet reconstructing the timing of the site's occupations at a seasonal scale is challenging. Micromorphological evidence suggests that, like today, the area suffered from winter freezing conditions, but there are too few faunal dental remains to conduct meso- and microwear analyses to establish seasonality reconstruction. Fortunately, other behavioral proxies at the site allow us to consider occupational duration, technological organization, mobility and subsistence strategies, and the sites' role in regional settlement systems.

Higher elevations sites such as Kalavan 2 experienced frost conditions as well as heavy snow cover during winter. Those conditions most likely would prohibit the accessibility of the area during this time of the year. Thus, Kalavan 2 will most likely have been occupied mainly during the rest of the year and for shorter periods. Considering the geomorphological evidence, interpretation of technological organization, raw material sourcing of the lithic assemblage, and the faunal evidence will be used to test the duration and intensity of occupation at the site.

From a lithic economy point of view, the assemblage has two complementary facets. In all units, obsidian artifacts outnumber non-obsidian ones (with the notable exception of T2 Unit 4; however, the earlier excavations of the same unit found more obsidian artifacts than non-obsidian ones [14]). The non-obsidian component includes a wide variety of raw materials including basalt, dacite, welded tuff, chert, and limestone. There are indications that early reduction stages of those materials occurred on-site (e.g., the presence of few non-obsidian cores and cortical pieces). However certain non-obsidian artifacts attest to their provisioning of blanks that where knapped off-site into the locale (e.g., a large Levallois point on basalt; Fig. 9: 3). [139] Such an indication suggests that the fragmentation of core reduction sequences across the landscape occurred at the non-obsidian components at the site [140]. The non-obsidian component also exhibits a greater variety of technological variants, such as flake (non-Levallois and Levallois) and blade production.

The obsidian assemblages include a high frequency of flakes < 2 cm in maximal dimensions, including high ratios of shaping flakes including those that removed the

tips of retouched points (Fig. 8: 1-5). Abundant small flakes occur despite the lack of on-site blank-production indicators (minimal numbers of cores and CTEs). This suggests intense on-site obsidian tool use, maintenance, and reduction compared to non-obsidian artifacts. The shaping flakes are categorized according to the scars on their dorsal face removing the previous phases of retouch. Most of the shaping flakes are < 2 cm. It is worth noting that the technological role(s) of shaping flakes might be diverse, especially because, in the current phase of research, this category is an inclusive term. Shaping flakes may reflect part of the initial stages of retouch or secondary retouch after use when the edge has become dull, jagged, or uneven (see ethnographic example [141]). These technological signatures reflect maintenance behaviors [142–144] intended to extend tool use-life. Obsidian retouched points and shaping flakes from the site exhibit use-wear, perhaps reflecting activities related to woodworking. These preliminary results suggest a scenario of continuous rejuvenation of retouched points during their use within an activity possibly woodworking. Initial use-wear observations for the obsidian scrapers, in contrast, hint at activities related to processing hard animal materials.

Similar obsidian modes of shaping that geared toward the removal and rejuvenation of the tips of retouched points have been documented at Yerevan 1 cave in its upper sequence [145]. The pieces include both lateral ([145], Fig. 14: 5), and distal [145], Fig. 14: 9, 15, 19; [19], Fig. 6: 9) shaping flakes. Yeritsyan and Semenov ([146]), based on use-wear analysis, suggested that the majority of analyzed obsidian tools were used for a diverse range of activities including skin scraping, wood, and bone processing, meat processing, perforating or cutting activities [146]. The main characteristic of the wear on the obsidian pieces is the crushing of the working edges. Both Yerevan 1 cave and Kalavan 2 are located beyond the expected daily foraging distance from the nearest obsidian sources. Obsidian sourcing has not been conducted for Yerevan 1 cave; however, the closest sources are ca. 30-40 km from the site [19]. It is not clear if such shaping techniques as observed in both sites are an outcome of raw material scarcity or rather an outcome of the obsidian raw material property that requires constant rejuvenation of the edges of the tools. A similar scenario was suggested when coarse grain dolerite was contrasted with the finer grain hornfels in the MSA layers at the site of Sibudu, South Africa, where it was suggested that dolerite edges are more durable in comparison to those made on hornfels that demanded constant rejuvenation by retouching [147]. Such suggestions regarding the effects of

raw material properties upon the maintenance behaviors at Kalavan 2 will be examined in the future using in-depth experimental, technological, refitting, and comprehensive use-wear analysis.

Sourcing of the non-obsidian raw materials at Kalavan 2 remains in the initial stages; however, obsidian sourcing by pXRF is well developed in Armenia. Obsidian is not available near Kalavan 2, and the closest sources lie well beyond the expected daily foraging range from the site following the principles of [148,149]. Their model assumptions are based on ethnographic hunter-gatherer studies that suggest that daily walking distance is confined to ca. 120 minutes one-way pedestrian travel and in a flat, homogeneous topography, this travel time is equal to a distance of ca. 10 km [148,149]. However, when applying Tobler's [150] formulae for the relationship between slope angle, distance, and walking time to the Armenian highland topography, would result in reduced distances than an area of circumference of ca. 10 km as suggested by [148,149].

Obsidian sourcing of 928 artifacts, conducted in the Kalavan 2 field lab, revealed that the Geghasar source in Gegham Range and sources along the Hrazdan River valley, in particular Gutansar and Hatis, were routinely exploited. The Tsaghkunyats sources were utilized to a lesser degree. These source areas are a linear distance of 50–60 km from Kalavan 2 but ≥ 120–140 km on foot. Less frequently, Syunik highlands sources were exploited as well: 120–130 km from the site linearly and at least ≥ 200 km along the most direct routes. Artifacts from the three distant obsidian sources (Pokr Arteni, Kars-Digor 1, and Chikiani) were found only in T1 Unit 1b; however, this might only be a function of sample sizes. Pokr Arteni is located ≥ 200 km to the west along the most direct routes from Kalavan 2. Further to the west lies Kars-Digor 1 in modern-day Turkey, 160 km linearly, and ≥ 200 km on foot from Kalavan 2. To the northwest of Kalavan 2, the farthest identified obsidian source is Chikiani in Georgia, 150 km linearly, and ≥ 240 km on foot (Fig. 13).

This raw material movement suggests contacts with other Late Pleistocene hominins. The source area of Gutansar and Hatis, was the most frequented and was shared with MP occupants of other sites such as Lusakert Cave 1 and Alapars-1 [23,25] (Fig. 12). The source of Pokr Arteni is located 1–2 km from the MP site of Barozh 12 [16,17,151]. Chikiani sources to the northwest were utilized at MP and UP occupations in the Imereti Region in the Rioni River basin (southern Caucasus), at a distance > 100 km [20,35,152]. The lithic assemblages at Ortvale Klde, a cave site in the Imereti

Region dated to MIS 3, show a similar duality in the raw material economy to that observed at Kalavan 2 [153]. Locally available chert quantitatively dominates the Ortvale Klde assemblage, which exhibits a low frequency of retouch, and the few obsidian artifacts were transported a notable distance and appear as highly reduced tools [35,153].

Barton and Riel-Salvatore [44] and Clark and Barton [45] modeled short-term occupations as lithic assemblages with low artifact densities, high frequencies of retouched pieces, and few cores (however see discussion in [46,154,155]). Plotting the frequency of retouched artifacts against artifact density for each unit with the highest artifact abundances, we observe that densities range between 20 and 307 artifacts/m^3 (Table 6) and the retouch frequencies range between 0.09 and 0.14 (Fig. 18). When these results are compared to those from the chronologically contemporaneous MP site of Barozh 12 [16,17,151], it is apparent that all of the Kalavan 2 units have very-low-density assemblages with significantly higher tool frequencies ($t = 4.9$, $df = 7$, $p = 0.002$; Fig. 17). Barozh 12 is a locality typified by very high artifact densities and a wide range of tool frequencies among sedimentary units [16,17,151]. Based on these data as well as the other lines of evidence discussed above, we propose that the Kalavan 2 assemblages are the outcome of relatively short-term occupations with activities that mainly involved tool use, maintenance and discard.

The faunal data may also be used to consider the occupation duration and site function within a wider settlement system. At task-specific sites dedicated to butchery activities, we expect monospecific faunal assemblages containing the non-nutritional bone elements (i.e., an assemblage with a narrow range of hunted animals and a high representation of non-nutritional elements), whereas residential sites should contain a diverse hunted faunal assemblage with meat-rich body parts represented (e.g., upper limbs, axial bones) [47,49]. Hominins played a major role in the formation of the Kalavan 2 faunal assemblages, as attested by the presence of anthropogenic bone modifications, lack of evidence for carnivore activity, and uneven spatial distribution of bones, mostly in association with lithic concentrations (tables 6 and 13). The varied ecological preferences of the present species could be the result of seasonal habitat overlap in the surroundings of Kalavan 2. Alternatively, it may reflect hominin use of different altitudinal zones/elevational ecotones [43] along the mountain flanks and subsequent transport of food to the site.

Our results from Kalavan 2 deviate from the expectations of a short-term kill and/or butchery site, as indicated by the animal diversity represented in the faunal assemblages and the representation of their body parts. The assemblages are biased toward a high representation of long bone fragments, which are less meat-rich. This pattern could be explained by either (1) post-depositional density-mediated attrition or (2) selective transport of skeletal elements to the site. Selective transport of body parts from kill sites to Kalavan 2 is likely given the rather steep landscape where such a decision optimizing the costs of transport. The preliminary results from our faunal analysis may suggest that Kalavan 2 was a secondary meat-processing or marrow consumption locale. However, the question wheater selected body parts were transported according to their nutritional value still needs to be further tested [156]. The various occupations at Kalavan 2 resemble short-term ephemeral visits to high elevations. Such a scenario is supported by sedimentological and micromorphological analyses as well as artifact densities and lithic assemblage composition.

Kalavan 2 adds to a growing corpus of MIS 3/2 sites that can be situated within wider subsistence patterns, especially concerning mobility strategies. At Ortvale Klde (530 m asl) in Georgia, faunal analysis suggests repeated exploitation of migratory herds of Caucasian tur (*Capra caucasica*) in late fall and/or early spring [35,157]. In Armenia, at Lusakert Cave 1 (1300 m asl), ca. 60–35 ka [24], the faunal assemblages consist of mainly *Capra* sp. and *Equus* sp., and a few *Bos/Bison* remains [158]. High densities of lithic finds, together with the preservation of hearth features [159], suggest that the site might have been occupied for a longer duration rather than a task-specific locale. At the UP site of Aghitu-3 (1601 m asl), where the main occupation layers are dated to 36–32 and 29–24 ka cal BP, the site occupations are characterized as short, repeated, and seasonal, exploiting mainly *Ovis* and equids in spring or summer based on tooth wear [15,156]. At the UP site of Kalavan 1, located only 1 km from Kalavan 2, repeated occupations dated to 16 ka cal BP were suggested to be seasonal occupations in summer and/or autumn, focused on hunting migrating mouflon (*Ovis orientalis*) based on isotopic and faunal analysis [36,160,161].

Among the above MIS 3 sites, some were assigned a putative role within settlement systems. Ortvale Klde was suggested to function as a residential site where different hunter-gatherer groups aggregated for economic and social purposes. This interpretation is based on the density of anthropogenic sediments and archaeological materials, especially in Layers 7–4 [35]. At Lusakert Cave 1, a similar line of evidence

was used to place the site as a focal point within the settlement system [158,159]. The contemporaneous site of Barozh 12 was also interpreted as a focal point in hominin settlement systems based on high find densities [16,17,151]. When comparing the find densities and the retouch intensity from Kalavan 2 and Barozh 12 (Fig. 17), sharp differences in technological organization, as reflected in different modes of settlement pattern, can be observed. At Barozh 12, relatively longer occupations focused on core reduction and blank production alongside tool use and discard [16,17,151]. Kalavan 2, on the other hand, reflects the opposite end of the continuum: repeated, short-term occupations with a narrow range of activities.

The geographic range of the settlement system in which Kalavan 2 occupants moved is illuminated by obsidian sourcing. Lithic procurement behaviors during MIS 3 in this region suggest mainly the exploitation of nearby raw material sources with infrequent transport of non-local material beyond the daily exploitation territory. Non-local obsidians were transported to sites over a range of 50-200 km as the crow flies and up to 300 km as the shortest walking distance [21,23,33,148,150,160]. Kalavan 2 is among the few MIS 3 sites where obsidian is not locally available (e.g., [19,21,145]. Our current obsidian sourcing results attest to a diversity of sources, the closest ones being at least 120–140 km on foot. The most distant sources reflect distances greater than 200 km on foot. Such distances appear much greater in comparison with those identified in Mediterranean Europe and the Levant in the same period [162–164]. Among the Mediterranean European and Levantine MIS 3 sites, nearly 90% of the lithic raw materials were derived from daily foraging territories [162–166]. The evidence from Kalavan 2, in contrast, attests to high-mobility lifeways over large territories.

Concluding remarks

Kalavan 2 is located in a rugged mountainous landscape where winter temperatures are freezing today and, as shown here, were likely similar during the Late Pleistocene. The available resources for hominins within such an ecological niche fluctuated drastically according to the seasons. As such, the site was most likely not occupied during the harsh winter periods. The results of the renewed excavations at Kalavan 2 refined the chronological, stratigraphical, and paleoenvironmental framework for contextualizing hominin activities within the mountainous landscape from ca. 60 to 45 ka. The rich behavioral and environmental corpus unearthed at

Kalavan 2 enables us to develop hypotheses regarding elevational mobility patterns and the nature of hominin-environmental interactions in a mountainous environment. Evidence from Kalavan 2 as observed in technological organization, resource procurement, and subsistence practices, have illuminated late MP hunter-gatherer lifeways and enables us to place the site within the settlement systems in the Armenian highlands.

Currently, Kalavan 2 is the only stratified, open-air site in the region with the preserved fauna in association with lithic artifacts within a stratified context. The faunal assemblage comprises of diverse hunted animals including *Bos*/bison, horse, wild goat/ibex, and red deer, dominated by ~~long bones~~ elements. These results suggest Kalavan 2 represents a secondary processing locale, rather than a hunting or primary butchery locale. The lithic assemblages contain two main technological components: (1) an obsidian assemblage with narrow techno-typological variation and an emphasis on highly reduced retouched pieces, hinting at a limited range of activities including woodworking and hard animal material; and (2) a non-obsidian assemblage with higher techno-typological variation, including knapping of flakes and blades. The geochemical sourcing of the obsidian artifacts largely circumscribe movements of the Kalavan 2 visitors to central Armenia, in particular, the vicinity of Hrazdan River (≥ 120-140 km on foot); however, there are also artifacts from four sources that lie ≥ 200 km on foot in different directions. Considered together, these lines of evidence support our interpretation of Kalavan 2 as the site of repeated, ephemeral occupations involving task-specific activities.

These results from Kalavan 2 illustrate that high-elevation locales played a pivotal and integral role in late MP settlement dynamics and behavioral adaptations in the rugged landscapes of Armenian highlands.

Acknowledgments

We would like to thank the Kalavan villagers for their help, support, and hospitality: especially the Ghukasyan family for providing us a home away from home. We warmly acknowledge the debt we owe to the students and volunteers that worked with us over the years. We also thank Suren Kesejyan, Hovhannes Partevyan, and Vardan Stepanyan. We express our sincere gratitude to the Institute of Archaeology and Ethnography of the National Academy of Sciences of the Republic of Armenia, and to Pavel Avetisyan, the Director of the Institute for Archaeology and Ethnography, National Academy of Sciences, Republic of Armenia, for his continued support of our research. For the essential financial and similar support, we gratefully thank the funding agencies of The Gerda Henkel Stiftung grant n. AZ 10_V_17 and n. AZ 23/F/19, the Leakey Foundation, and the Gfoeller Renaissance Foundation of USA. RT, AC, and SB would like to acknowledge support from the Leverhulme Trust-funded Palaeolithic Archaeology, Geochronology, and Environments of the Southern Caucasus (PAGES) project, and to thank Dr. C. Hayward at the University of Edinburgh for assistance with the microprobe analyses. VO et OB were supported by the ECCOREV Research Federation (Aix-Marseille Université, CNRS) and Labex OT-Med (n° ANR- 11-LABX-0061) funded by the French Government "Investissements d'Avenir" programme of the French National Research Agency (ANR) through the A*MIDEX project (n°ANR-11-IDEX-0001-02).
AMB and PG dedicate this paper to the memory of their grandmothers Hanna Malinsky and Bessie Singer who laid the foundation of the current project with their spirit and attitude.

Figure captions

Fig. 1: Location map of Kalavan 2 within southern Caucasus and north-eastern Armenian highlands main Lower, Middle and Upper Paleolithic sites

Fig. 2: Plateau topography and location of trenches at Kalavan 2

Fig. 3: Geological map of Kalavan area showing the larger watershed of the Barepat and in blue line the Dani watershed

Fig. 4: A: Section drawing of the Kalavan 2, Trench 1 north section including sample locations; B: Section drawing of the Kalavan 2, Trench 1 south section including sample locations

Fig. 5: Kalavan 2, Trench 2 east and south sections with sample locations

Fig. 6: Kalavan 2, Trench 3 south section with sample locations

Fig. 7: Kalavan 2, Trench 4 north section with sample locations

Fig. 8: Obsidian lithic artifacts: 1, 4-5. Shaping flakes that remove the tip of convergent retouched point; 2-3. Shaping flakes; 6-7. Retouched points.

Fig. 9: 1. Non-obsidian lithic artifacts: blade made on volcanic tuff; 2. cortical flake on volcanic tuff; 3. Levallois point made on Basalt; 4. Core on split pebble made on metamorphic material.

Fig. 10: Fragment of a retouched blade (upper left) and endscaper (upper right) with striations and polish associated with hard animal material. A retouched point (bottom left, Fig. 8:6) and a shaping flake of a retouched point (bottom right, Fig. 8:1) showing striations and polish diagnostic of woodworking activities.

Fig. 11: Limestone pitted anvil showing evidence of a) impact marks and b) surface crushing. Bottom: c) Limestone slab with abraded surface and percussion impact feature; d) Limestone pitted anvil with several impact marks and multidirectional flake scars resulting from percussive actions (arrows indicate the orientation of the flakes removal).

Fig. 12: Locations of obsidian sources relative to Kalavan 2 (SRTM3 digital elevation data). Presence or absence of the obsidians at Kalavan 2 is represented by each source's circular symbol. The empty circles correspond to obsidian sources that are not currently reflected in the Kalavan 2 assemblage.

Fig. 13: Obsidian sources and the corresponding source areas identified at Kalavan 2 by trench.

Fig. 14: Obsidian source areas identified in all trenches by lithic class.

Fig. 15: Kalavan 2 Cervid (red deer sized) metacarpus with multiple cut marks (Id: N50-44)

Fig. 16: Kalavan 2: Large bovid femur diaphysis with impact notch (Id: A77-1)

Fig. 17: Binary plots of artifact density versus retouch frequency for sedimentary units at Barozh 12 (open circles) and Kalavan 2 (filled circles), with obsidian artifacts

(A) and non-obsidian artifacts at Kalavan 2 (B). Data exclude artifacts < 2 cm in maximal dimensions to avoid differences among sedimentary units in assemblage preservation and winnowing of small artifacts due to post-depositional processes.

Unit	Description	Color	pIRIR225 results	Micro-morph	Lithic	Fauna	Sediment sources	Interpretation
	Gravels and small pebbles grain size coarsening upwards. Pebbles size at their long axis ranges between 0.5-1 cm.	7.5 yr 3/3 dark brown					Dani watershed	No Mg, granulometric classification showing torrential sediment transport.
1b	Silty sand deposit with diffused rounded gravels manganese and iron linear concentrations	7.5 yr 3/3 dark brown	54.9 ± 3.8	North section (MM3)	+	+	Barepat watershed influence	Mg 1,7% gravel abrasion (rounded) showing significant longitudinal transport. More Si than level 1a (>20%) and the appearance of aluminum element (Al>8%). The presence/apparition of potassium (K=1,19%) is probably linked to reworking of the soils and endogenic rocks of the surrounding slopes and watershed.
2a	Gravely pebbly level with silty sand matrix with heteromeric gravel pebble 3-4 cm, sub-angular sub-rounded. At the basis of the Unit intensive manganese and iron concentration.	7.5 yr 3/2 dark brown		North section			Barepat watershed influence, discrete mixing with Dani	Mg 1,9% Si >20% Al around 8% Manganese (Mn) and Iron (Fe) concentration showing fluctuations of the water table in a quasi-permanent flowed/saturated sediment. Dani sediment supply could be related to sub-angular elements and a higher rate of carbonated rocks (Ca >6% instead of 1 to 3% in the underlying levels). The presence of potassium (K) is probably linked to the reworking of the soils and endogenic rocks of

								the surrounding slopes and watershed.
2b	Silty sand level with diffused gravels subrounded and rounded, 5 mm, sub polyhedric structure	7.5yr 3/3 dark brown		North section	+	+	Dani watershed	Pedogenetic features showing less sediment input from Barepat river indicating the lateral movement of the Barepat riverbed.<br>No Mg.<br>The presence of Al and the poorer Ca rates underline the stasis of the deposition and the remnants and pedogenic evolution of some sediment supplied by the Barepat. The presence of potassium is probably linked to the reworking of the soils and endogenic rocks of the surrounding slopes and watershed.
3a:	Pebble level with small gravel of 5-6 cm. Silty sand matrix. Calcareous and siliceous blocks 10-40 cm.	10yr 4/3 brown					Dani watershed	Same elemental characteristics as Unit 2b. In addition, the presence of bulky calcareous blocks which are the main lithological signature of the Dani river watershed.
3b:	Sandy silty with fine gravel of 1 mm in longest axis, diffused pebbles. In the middle of the unit, there is a line of small rounded sub-rounded pebbles less than 1 cm. in max axis.	10yr 4/3 brown	52.7 ± 3.7				Barepat watershed influence	Presence of Mg (1,4%), higher rates of<br>Si (>20%) and lower rates of<br>Ca (<1,8%).<br>Rounded pebbles showing significant longitudinal transport and a higher quantity of thin grained sediment. The presence of potassium (K) is probably linked to the recycling of the soils and endogenic

								rocks of the surrounding slopes and watershed.
4:	Gravely layer, pebbles sub-rounded and sub-angular element, heteromeric between 1 cm to 10 cm of max. axis. Sandy matrix and some spots of manganese and altered pebbles	10 yr 3/4 dark yellowish-brown					Barepat watershed influence, mixing with Dani	The presence of 1,4% of Mg, high rates of Si (>23%), but higher rates of Ca (>3%), sub-rounded, and sub-angular calcareous pebbles could be the signature of a sedimentary input from the Dani river. The remarkable growth of the Potassium (K=2,44%) is probably linked to the erosion of the surrounding soils from the Dani watershed.
5:	Sandy silty layer with very small rounded gravel but very diffused, with traces of carbonates associated with the roots.	10 yr 4/3 brown	59.4 ± 4.5	South section (MM4	+	+	Barepat watershed influence, mixing with Dani	Presence of 1,2% of Mg, still high rates of Si (>19%), and rates of Ca (>3%). Again, sub-rounded and sub-angular calcareous pebbles. Here again, the presence of Potassium is probably more linked to the erosion of the surrounding soils from the Dani watershed.
6:	Sandy layer with little silt with very small sub-rounded gravel	10yr 5.2 grayish brown	46.4 ± 4.1	South section	+	+	Barepat watershed influence, mixing with Dani	Presence of 1,8% of Mg, rates of Si (>10%), and sub-rounded calcareous pebbles. The highest punctual rates of Ca (>12%) could be the signature of a brief Dani river sedimentary input.
7	Top soil- silty argillaceous clay with polyhedric structure with small gravel angular around 1 cm. in max. axis	7.5.yr 3/2 dark brown			South section		Mainly Barepat watershed.	The presence of 1,7% of Mg, growing rates of Si (>14%), as well as a significant drop of Ca rates (>6%) and thin grained deposits plead for a major sediment supply from the Barepat watershed.

								However, the small angular gravels and the re-appearance of K (>1.1%) could indicate a discrete input from the Dani watershed slopes.

Table 1: Description and interpretation of sedimentological units (from bottom to top) in Kalavan 2, Trench 1

Unit	Description	Color	pIRIR225 results	Micro-morph	Lithic	Fauna	Sediment sources	Interpretation
1a	Pebbles and blocks, sub-rounded heteromeric, 5-30 cm. The gravel matrix sand and gravel, 1 to 5 cm. Angular, subangular, middle size sand, and 1% silt.	10yr 4/3 brown					Dani watershed	Calcareous blocks and homogenous elemental composition similar to the sediment supply generally attributed to the Dani watershed. To be noticed the presence of Al (1,1%) which can be linked with a relative proximity of the Barepat water table. The presence of Potassium (K) is probably linked to the erosion of the soils from the surrounding slopes of the Dani catchment.
1b	Silty sand deposits with diffused gravels, 1 mm, sub abraded, structured lightly polyhedric, week pedogenesis. Sandy lens with coarse grain and medium sand	Color: 10yr 3/4 dark yellowish-brown	55.3 ± 4.1				Dani watershed	Same characteristics as in level 1a. The presence of Potassium (K) is probably linked to the erosion of the soils from the surrounding slopes but also to a weak pedogenetic development observable in the sediment structure.
2a	Gravel- matrix – small pebbles, 5-25 cm, and sands heteromeric, sub-angular to sub-rounded with traces of manganese and iron and diffused carbonates.	4/2 dark grayish brown					Dani watershed	Same general characteristics as observed in the previous levels. The sub-angular to sub-rounded pebbles indicates a modest longitudinal sedimentary transit following a Dani river origin. The presence of manganese (Mn=0,8%) is linked to a rise and fluctuations of the water table.
2b	Silty sand with carbonate traces very diffused gravel, linked the contact with another gravel,	10yr 4/3 brown					Barepat watershed influence	Appearance of the Magnesium (Mg= 1,7%). Decrease in Ca, rounded gravels and fine-grained sediment.

	erosive contact gravel rounded								
3:	Gravel, sand and gravel matrix, heteromeric 5 mm – 4 cm, sub-angular to sub-rounded, some of the blocks (clasts) size range 10 – 25 cm. generally sub-rounded and rounded, upper part is weathered.	10yr 4/3 brown					Dani watershed	Same general characteristics as observed in the underlying unitsattributed to Dani river. The sub-angular to sub-rounded pebbles and the calcareous blocks indicate a modest longitudinal sedimentary transit in accordance with a Dani river origin. The upper part of the level shows traces of the progressive influence of an another Barepat sedimentary supply.
4:	Silty sand with small aggregate of secondary carbonatation, with grass roots , structure polyhedric but very light.	4/3 brown	56.9 ± 4.4	MM1	+	+	Barepat watershed influence	Highest magnesium values (Mg= 2,3%) in the sequence, indicating Barepat influence. Elemental composition and weak pedogenetic evolution, suggests sediment structure/composition could be evidence of a proximal “floodplain” position relative to the Barepat River.
5:	Medium size sand with small gravels ca.1 mm, sub-angular to angular.	2.5y 5/3 light olive brown		MM2	+	+	Barepat watershed influence mixed with Dani sedimentary supply	The low Mg (1,5%) andAl values, and a higher Ca value (9,15%) compared to overlying Unit 4, could indicate a bigger mix between the Barepat and the Dani sediment supply/waters.
6:	Siltly sand with very diffused pebbles, 0.5 – 2 cm, sub rounded and heterometric.	10yr 4/3 brown		MM2	+	+	Dani watershed	Same general characteristics as observed in units attributed to the Dani river.
7:	Silty clay with diffused gravel, polyhedric	10yr 4/2 dark grayish brown	44.1 ± 3.4		+	+	Dani watershed	Same general characteristics as observed in units attributed to Dani river.
8:	Top soil- silty clay soil more structured	10yr 2/2 – very dark brown					Dani watershed	Same general characteristics as observed in units

	than Unit 7, with sub-angular to angular gravels ca 1 mm to 5mm.							attributed to the Dani river.

Table 2: Description and interpretation of sedimentological unit (from bottom to top) in Kalavan 2, Trench 2

Unit	Description	Color	OSL sample	Lithic	Fauna	Sediment sources	Interpretation
1	Silty-sand sediments with small carbonated roots with light polyhedric structure	10yr 3/2 very dark grayish brown	56.6±4.4	+		Barepat watershed influence	Mg (2,5%), Fine-grained sedimentation, floodplain environment.
2a	Pebbly gravely with sub-angular - subrounded gravel and pebbles, heteromeric, 2 mm - 30 cm. and sand.	Color: 10yr 3/4 dark yellowish -brown				Dani watershed	Same general characteristics as observed in units attributed to Dani river. The presence of sub-angular to angular gravel and pebbles shows a short longitudinal sedimentary transit.
2b	Sandy-silt with very diffused angular to sub-angular gravels, 2-3 mm, traces of carbonates roots, polyhedric structure.	10yr 3/3 dark brown	62.7 ± 4.5	+	+	Barepat watershed influence mixed with Dani river	Mg (1,8%), fine- grained sedimentation but angular to sub-angular gravels showing intermittent input from slopes, local channel, and surface runoff.
3a:	Gravel- pebbly with heteromeric pebbles, subrounded and sub-angular, carbonated. Sandy silt matrix.	10yr 4/2 dark grayish brown				Dani watershed	Same general characteristics as observed in units attributed to Dani river. The presence of sub-angular to angular gravel and pebbles shows a short longitudinal sedimentary transit.
3b:	Sandy-silt with some pebbles diffused sub-rounded 5 mm - 3 cm. Traces of root	10yr 4-4 Dark yellowish brown	60.3 ± 4.4			Dani watershed	Same general characteristics as observed in units attributed to Dani river.

	carbonates, carbonate aggregates						
4a:	Alluvial reworked volcanic sand with gravels < 1 - 5 mm	2.5.y 2/5/1 Black		+	+	Dani watershed	Same general characteristics as observed in units attributed to Dani river. The presence of sub-angular to angular gravel and pebbles shows a short longitudinal sedimentary transit.
4b:	Sand with rare gravels subrounded. 0.5 cm, the level reworked the previous unit	4a/ black		+	+	Dani watershed	Same general characteristics as observed in units attributed to Dani river. The presence of the highest Ca value (6,48%) confirmssediment supply from Dani watershed.
5:	Reworked of unit 4b					Dani watershed	Same general characteristics as observed in units attributed to Dani river. The sediment reworking is visible in the elemental composition
6:						Dani watershed	Same general characteristics as observed in units attributed to Dani river.
7:	Top soil, silty sandy with sub-angular gravels diffused top part polyhedric structure, with charcoal	10yr 4/2 dark grayish brown				Dani watershed	Presence of sub-angular gravels and discrete development of a soil formation shows a short longitudinal sedimentary transit.

Table 3: Description and interpretation of sedimentological unit (from bottom to top) in Kalavan 2, Trench 3

Unit	Description	Color	OSL	Lithic	Fauna	Sediment sources	Interpretation
1:	Thin bedded medium sands, diffused and alternated gravels, 2 mm	5y 2.5/1 black				Dani watershed	Same general characteristics as observed in units attributed to Dani river. Presence of small gravels showing surface runoff from the Dani watershed.
2:	Medium-size sand with sub-rounded, diffuse gravels 1-5 mm	5y 3-1 very dark black grayish	59.5 ± 6.1	+	+	Dani watershed	Same general characteristics as observed in units attributed to Dani river.
3:	Sandy silt level with very diffuse small gravel subangular, traces of roots and bioturbation with light pedogenesis.	Color: 2.5y 3/2 very dark brown grayish				Dani watershed with Barepat watershed influence	The presence of Mg (1,8%) could indicate a former sedimentary supply from the Barepat mixed with the Dani river.
4:	Sandy silt with small carbonated roots, with sand lenses	10yr 4/4 dark grayish brown	51.3 ± 4.8			Dani watershed	Same general characteristics as observed in units attributed to Dani river. Carbonated roots indicate a soil development.
5a:	Silty sand carbonated with a root traces, bioturbation and polyhedric structure	10yr 5.2 grayish brown		+		Dani watershed with Barepat watershed influence	The presence of Mg (1,5%) indicates a discrete sedimentary supply from the Barepat mixed with Dani river input.
5b:	Sandy-silt level with carbonate nodules, sub-angular gravel heteromeric 1-3 mm and discrete polyhedric structure	10yr 4/2 dark grayish brown		+		Dani watershed	Most of the level characteristics are observed in units attributed to Dani river.
5c:	Sandy silt with carbonate root casts with gypsum, polyhedric structure	10yr 3/2 very dark grayish brown		+		Dani watershed	Same general characteristics as observed in units attributed to Dani river. Gypsum

							underline the influence of the alluvial context (humidity variation) in the pedogenesis.
5d	Sandy-silt with very diffuse, rounded gravels	10yr 4/3 brown			+	Barepat watershed influence	The presence of the highest Mg values in the sequence (2,1%) indicate a sedimentary supply from the Barepat. Fine-grained sedimentation and rounded gravels support this observation.
6a:	sandy silt level, ash and, reworked dark sand	10yr4-2 dark grayish brown				Dani watershed	Some similar characteristics as observed in units attributed to Dani river but with the presence of ash and dark sand.
6b:	Silty-sand with roots traces and very diffuse, small aggregates of sediment	10yr 4-3 brown		+	+	Dani watershed	Same general characteristics as observed in units attributed to Dani river.
7:	Silty sand with root traces, little carbonate, polyhedric structure	10yr 5-2 grayish brown		+	+	Dani watershed	some similar characteristics as observed in units attributed to Dani river but with light pedogenic process.
8:	silty sandy roots with carbonates polyhedric structure	10yr 4/3 brown	36.2 ± 2.7		+	Dani watershed	Same general characteristics as observed in units attributed to Dani river, roots presence indicate the first development of pedogenesis.
9:	Sand with small carbonates aggregate	10yr 4/3 brown				Barepat watershed influence and Dani input.	Presence of Mg (1.3%) and a fine-grained sedimentary supply. High Ca value

							(9,15%) indicates weathering potentially associated with sedimentary supply from the Dani.
10a:	Silty sand layer carbonated with small and diffused sediment aggregates, light polyhedric structure with bioturbation	10yr 6/3 pale brown				Dani watershed	Same general characteristics as observed in units attributed to Dani river. Bioturbation and polyhedric structure underline the first step of the development of a soil.
10b:	Top soil: silty sand with small clay component, sub-angular gravels 0.5 to 2 cm, blocky structure	7.5 yr 3/2 Dark brown				Dani watershed	Same general characteristics as observed in units attributed to Dani river, then development of a top soil.

Table 4: Description and interpretation of sedimentological unit (from bottom to top) in Kalavan 2, Trench 4

Field code	Sample ID	total doserate (mGy/a)	De pIRIR225 (Gy)	pIRIR225 age (ka)
Kal-2-1-0	1681	2.9 ± 0.3	135 ± 1.1	46.4 ± 4.1
Kal-2-1-1	1682	3.3 ± 0.3	198 ± 1.1	59.4 ± 4.5
Kal-2-1-2	1683	4.1 ± 0.3	214 ± 1.5	52.7 ± 3.7
Kal-2-1-3	1684	3.6 ± 0.2	198 ± 1.9	54.9 ± 3.8
Kal-2-2-1 (2017)	1685	3.9± 2.7	109 ± 6.6	32.2 ± 3.2
Kal-2-2-1 / 2018	1688	3.9± 2.7	10.6 ± 0.1	2.7 ± 0.2
Kal 2-2-1/ 2018	1904	3.1 ± 0.2	138 ± 1.2	44.1 ± 3.4
Kal-2-2-2	1686	3.6 ± 0.3	204 ± 1.4	56.9 ± 4.4
Kal-2-2-3	1687	3.8 ± 0.3	208 ± 1.8	55.3 ± 4.1
Kal 2-3-1/ 2018	1906	3.6 ± 0.3	224 ± 3.0	62.7 ± 4.5
Kal-2-3-2	1689	3.6 ± 0.3	218 ± 1.7	60.3 ± 4.4
Kal-2-3-3	1690	3.4 ± 0.3	195 ± 2.6	56.5 ± 4.4
Kal-2-4-2	1692	2.6 ± 0.2	134 ± 1.4	51.3 ± 4.8
Kal 2-4-1	1691	3.5 ± 0.3	127 ± 0.6	36.2 ± 2.7
Kal 2-4-3	1693	3.0 ± 0.2	177 ± 11.0	59.5 ± 6.1

Table 5: pIRIR225 results (4-11 µm) from Kalavan-2

T1	**Unit 5-6** (8.5 m3)	**Items ≥2 cm**	**Items ≤2 cm**	**Density per M3 ≤2 cm**	**Total**	**Density of lithic artifacts per M3**
Obsidian	**N**	49	71		**120**	
	**%**	40.8	59.2		100	
Non-Obsidian	**N**	30	68		**98**	
	**%**	30.6	69.4		100	
**Total**	**N**	**79**	**139**	**16**	**218**	**27**
	**%**	36.2	63.8		100	
	**Unit 1a** (3.4 m3)	**Items ≥2 cm**	**Items ≤2 cm**	**Density per M3 ≤2 cm**	**Total**	**Density of lithic artifacts per M3**
Obsidian	**N**	748	131		879	
	**%**	85.1	14.9			
Non-Obsidian	**N**	77	88		165	
	**%**	46.7	53.3		100	
**Total**	**N**	**825**	**219**	**64**	**1044**	**307**
	**%**	79.0	21.0		100	
T2	**Unit 4** (2.6 m3)	**Items ≥2 cm**	**Items ≤2 cm**	**Density per M3 ≤2 cm**	**Total**	**Density of lithic artifacts per M3**
Obsidian	**N**	62	46		**108**	
	**%**	57.4	42.6		100	
Non-Obsidian	**N**	153	208		**361**	
	**%**	42.4	57.6		100	
**Total**	**N**	**215**	**254**	**98**	**469**	**180**
	**%**	45.8	54.2		100	
	**Unit 5-6** (3.9 m3)	**Items ≥2 cm**	**Items ≤2 cm**	**Density per M3 ≤2 cm**	**Total**	**Density of lithic artifacts per M3**
Obsidian	**N**	23	39		**62**	
	**%**	37.1	62.9		100.0	
Non-Obsidian	**N**	2	14		**16**	
	**%**	12.5	87.5		100	
**Total**	**N**	**25**	**53**	**14**	**78**	**20**
	**%**	32.1	67.9		100	
	**Unit 7** (2.6 m3)	**Items ≥2 cm**	**Items ≤2 cm**	**Density per M3 ≤2 cm**	**Total**	**Density of lithic artifacts per M3**
Obsidian	**N**	161	123		**284**	
	**%**	56.7	43.3		100	

Non-Obsidian	**N**	23	45		**68**	
	**%**	33.8	66.2		100	
**Total**	**N**	**184**	**168**	**65**	**352**	**135**
	**%**	52.3	47.7		100	

Table 6: Quantities and denisities of lithic artefacts in the different trenches and sedimentological units at Kalavan 2.

	**Unit 5-6**				**Unit 2b**				**Unit 1b**			
	**Obsidian**		**Non-obsidian**		**Obsidian**		**Non-obsidian**		**Obsidian**		**Non-obsidian**	
	**N**	**%**	**N**	**%**	**N**	**%**	**N**	**%**	**N**	**%**	**N**	**%**
**Core**	1	0.8	-	-	-	-	-	-	3	0.3	-	-
**Core-on- flake**	-	-	-	-	-	-	-	-	1	0.6	1	0.6
**Cortical element**	-	-	-	-	-	-	-	-	-	-	2	1.2
**Flake**	28	23.3	54	55.1	-	-	4	44.4	51	5.8	65	39.4
**Kombewa flake**	2	1.7		0.0	-	-	-	-	-	-	-	-
**Levallois flake**	2	1.7	4	4.1	-	-	-	-	-	-	-	-
**Levallois point**	-	-	-	-	-	-	-	-	-	-	2	1.2
**Levallois blade**	-	-	-	-	-	-	-	-	-	-	1	0.6
**Blade**	2	1.7	4	4.1	-	-	-	-	2	0.2	2	1.2
**Bladelet**	4	3.3	2	2.0	-	-	-	-	-	-	-	-
**Naturally backed knife**	-	-	-	-	-	-	-	-	-	-	1	0.6
**Core trimming element**	3	2.5	2	2.0	-	-	-	-	2	0.2	1	0.6
**Tool**	15	12.5	2	2.0	3	4.2	2	22.2	23	2.6	7	4.2
**Chip**	49	40.8	30	30.6	69	95.8	3	33.3	748	85.1	77	46.7
**Shaping flake**	14	11.7		0.0	-	-	-	-	50	5.7	-	-
**Chunk**	-	-	-	-	-	-	-	-	-	-	1	0.6
**Hammerstone**	-	-	-	-	-	-	-	-	-	-	4	2.4
**Total**	120	100	98	100	72	100	9	100	879	100	165	100

Table 7: The techno-typological composition of T1-Units 5-6,2b and 1b assemblages. of Trench 1 lithic assemblages

Unit 4	Obsidian		Non-obsidian	
	N	%	N	%
Core	-	-	5	1.4
Cortical element	-	-	3	0.8
Flake	10	9.3	145	40.2
Kombewa flake	1	0.9	5	1.4
Levallois flake	-	-	1	0.3
Levallois point	-	-	2	0.6
Levallois blade	-	-	2	0.6
Blade	2	1.9	16	4.4
Bladelet	-	-	2	0.6
Naturally backed knife	-	-		0.0
Core trimming element	2	1.9	7	1.9
Tool	12	11.1	11	3.0
Chip	62	57.4	153	42.4
Shaping flake	19	17.6	6	1.7
Hammerstone	-	-	2	0.6
Chunk	-	-	1	0.3
**Total**	**108**	**100**	**361**	**100**

Table 8: The techno-typological composition of Trench T2-Unit 4 lithic assemblage

	Obsidian		Non-obsidian	
	**N**	**%**	**N**	**%**
**Core**	-	-	-	-
**Cortical element**	-	-	-	-
**Flake**	12	19.4	9	56.3
**Kombewa flake**	-	-	-	-
**Levallois flake**	-	-	-	-
**Levallois point**	-	-	-	-
**Levallois blade**	-	-	-	-
**Blade**	-	-	1	6.3
**Bladelet**	-	-		0.0
**Naturally backed knife**	-	-	-	-
**Core trimming element**	-	-	2	12.5
**Tool**	3	4.8		0.0
**Chip**	23	37.1	2	12.5
**Shaping flake**	24	38.7	-	-
**Hammerstone**	-	-	-	-
**Chunk**	-	-	2	12.5
**Total**	62	100	16	100

Table 9: The techno-typological composition of Unit 5-6 lithic assemblage

**Kalvan 2- T2- Unit 7**	**Obsidian**		**Non-obsidian**	
	**N**	**%**	**N**	**%**
**Core**	1	0.4	1	1.5
**Cortical element**	1	0.4	3	4.4
**Flake**	30	10.6	25	36.8
**Kombewa flake**	-	-	-	-
**Levallois flake**	1	0.4	1	1.5
**Levallois point**	-	-	1	1.5
**Levallois blade**	-	-	1	1.5
**Blade**	-	-	7	10.3
**Bladelet**	5	1.8	1	1.5
**Naturally backed knife**	-	-	-	-
**Core trimming element**	6	2.1	-	-
**Tool**	12	4.2	3	4.4
**Chip**	161	56.7	23	33.8
**Shaping flake**	66	23.2	2	2.9
**Hammerstone**	-	-	-	-
**Chunk**	1	0.4	-	-
**Total**	284	100	68	100

Table 10: The techno-typological composition of Trench T2-Unit 7 lithic assemblage

T3						
	Unit 4		Unit 2b		Unit 1	
	Obsidian	Non-obsidian	Obsidian	Non-obsidian	Obsidian	Non-obsidian
	N	N	N	N	N	N
**Core**	-	-	-	-	-	-
**Cortical element**	-	-	-	-	-	-
**Flake**	3	3	-	10	-	-
**Kombewa flake**	-	-	-	-	-	-
**Levallois flake**	-	-	- -	-	-	-
**Levallois point**	-	-	- -	-	-	-
**Levallois blade**	-	-	- -	-	-	1
**Blade**	-	-	- -	2	-	-
**Bladelet**	1	-	-	-	-	-
**Naturally backed knife**	-	-	- -	-	-	-
**Core trimming element**	1	-	-	-	-	-
**Tool**	3	-	2	-	-	
**Chip**	10	-	-	2	-	2
**Chunk**	-	-	-	-	-	-
**Total**	18	3	2	14	-	3

Table 11: The techno-typological composition of Trench T3- lithic assemblages

T4								
	**Unit 7**		**Unit 6**		**Unit 5**		**Unit 2**	
	**Obsidian**	**Non-obsidian**	**Obsidian**	**Non-obsidian**	**Obsidian**	**Non-obsidian**	**Obsidian**	**Non-obsidian**
	**N**	**N**	**N**	**N**	**N**	**N**	**N**	**N**
**Core**	-	-	-	-	-	-	-	-
**Cortical element**	-	-	-	-	-	-	-	-
**Flake**	-	-	-	3	6	2	2	1
**Kombewa flake**	-	-	-	-	-	-	-	-
**Levallois flake**	-	-	-	-	-	-	-	-
**Levallois point**	-	-	-	-	-	-	-	-
**Levallois blade**	-	-	-	-	-	-	-	-
**Blade**	-	-	-	-	-	-	-	-
**Bladelet**	-	-	-	-	1	-	-	-
**Naturally backed knife**	-	-	-	-	1	-	-	-
**Core trimming element**	-	-	-	-	1	-	1	-
**Tool**	-	-	2	-	4	-	-	-
**Chip**	1	4	-	-	30	9	5	-
**Chunk**	-	-	-	-	-	-	-	-
**Total**	**1**	**4**	**2**	**3**	**41**	**11**	**8**	**1**

Table 12: The techno-typological composition of Trench T4- lithic assemblages

**Trench 1**	**Unit**	**5/6**	**3b**	**3a**	**2b**	**1b**	**total**
	NSP	183	17	13	74	764	**1051**
	% of T1	17.4	1.6	1.2	7.0	72.7	**100**
	% of total	8.5	0.8	0.6	3.4	35.3	**48.6**
	Density per M^3	22	-	-	15	90	
**Trench 2**	**Unit**	7	**5/6**	**4**	-	-	
	NSP	129	117	854	-	-	**1100**
	% of T2	11.8	10.7	77.6	-	-	**100**
	% of total	5.9	5.6	39.5	-	-	**50.1**
	Density per M^3	50	30	328	-	-	
**Trench 3**	**Unit**	**4**	**2b**				
	NSP	1	3	-	-	-	**4**
	% of T3	25	75	-	-	-	**100**
	% of total	0.1	0.1	-	-	-	**0.2**
**Trench 4**	**Unit**	**8**	7	**6b**	**5d**	**2**	
	NSP	3	2	1	1	1	**8**
	% of T4	37.5	25	12.5	12.5	12.5	**100**
	% of total	0.1	0.1	0.1	0.1	0.1	**0.4**

Table 13: Number of bones and density per m^3 from Kalavan 2 by trench and sedimentological unit

Anat. region	**Trench 1**						
**Unit**	**1b**	**2a**	**2b**	**3a**	**3b**	**5**	
**Cranial**	12	1	3			4	**20**
**Axial**	11	1	3	1		2	**18**
**Long bone**	83	2	2	2	1	6	**96**
**Autopodium**	8		1	2		2	**13**
	114	4	9	5	1	14	**147**
	**Trench 2**						
**Unit**	3	4	5	5/6	6	7	
**Cranial**		17		1		1	**19**
**Axial**		4					**4**
**Long bone**	2	10	1				**11**
**Autopodium**		4			1		**5**
	**2**	**35**	**1**	**1**	**1**	**1**	**39**
	**Trench 3**						
**Unit**	**2b**	**4**					
**Cranial**	2						**2**
**Axial**	1						**1**
**Long bone**		1					**1**
**Autopodium**							
**Total**	**3**	**1**					**4**
	**Trench 4**						
	**2**	**5d**	**6b**	**7**	**8**		
**Unit**					1		**1**
**Cranial**		1			1		**2**
**Axial**			1				**1**
**Long bone**	1			2	1		**4**
**Autopodium**							**0**
**Total**	**1**	**1**	**1**	**2**	**3**		**8**

Table 14: Number of individual specimens (NISP) of anatomical region by trench and sedimentological unit. Anat. region = anatomical region.

**Trench 1**	*Species*					
Unit	**Bos/Bison**	**Equus sp.**	**Equus hemionus**	**Cervus elaphus**	**Capra/Ovis**	**Total**
**5**	1				1	2
**3a**	1					1
**2b**	3	1				4
**2a**	1					1
**1b**	17	7		5	2	31
**Total**	**23**	**8**		**5**	**3**	**39**
**Trench 2**	*Species*					
Unit	**Bos/Bison**	**Equus sp.**	**Equus hemionus**	**Cervus elaphus**	**Capra/Ovis**	
7		1				1
**5/6**			1			1
**5**	1					1
**4**	16	6	2	4	1	29
**Total**	**17**	7	**3**	**4**	**1**	**32**
**Trench 3**	*Species*					
Unit	**Bos/Bison**	**Equus sp.**	**Equus hemionus**	**Cervus elaphus**	**Capra/Ovis**	
**2b**	2					2
**Total**	**2**					**2**
**Trench 4**	*Species*					
Unit	**Bos/Bison**	**Equus sp.**	**Equus hemionus**	**Cervus elaphus**	**Capra/Ovis**	
**8**					3	3
**6b**					1	1
**5d**					1	1
**2**				1		1
				**1**	**5**	**6**

Table 15: NISP of Species by trench and sedimentological unit

**Trench 1**	*Size class*						
Unit	**4**	**3 or 4**	**3**	**2 or 3**	**2**	**1**	**Total**
**5**	5	5	5	11	4		**30**
**3b**	1						**1**
**3a**	3	3	2				**8**
**2b**	6	2	2				**10**
**2a**	2		1	2	1		**6**
**1b**	106	37	30	25	21		**219**
**Total**	**123**	**47**	**40**	**38**	**26**		**274**
**Trench 2**	*Size class*						
Unit	**4**	**3 or 4**	**3**	**2 or 3**	**2**	**1**	
7		3		1			**4**
**6**		11			1		**12**
**5/6**	2	8	1		1		**12**
**5**	1	9					**10**
**4**	123	148	36	16	27	1	**351**
**3**			2				**2**
Total	**126**	**179**	**39**	**17**	**29**	**1**	**391**
**Trench 3**	*Size class*						
Unit	**4**	**3 or 4**	**3**	**2 or 3**	**2**	**1**	**Total**
**4**				1			1
**2b**	2	1					3
**Total**	**2**	**1**		**1**			**4**
**Trench 4**	*Size class*						
Unit	**4**	**3 or 4**	**3**	**2 or 3**	**2**	**1**	**Total**
**8**				3			**3**
7					2		**2**
**6b**				1			**1**
**5d**				1			**1**
**2**			1				**1**
**Total**	-	-	**1**	**5**	**2**	**0**	**8**

Table 16: Number of specimens (NSP) of size class by trench and sedimentological unit

Trench	Unit	ACL	OEP	Grass (%)[1]	Landscape[2]
1	6	29.8	9.9	75%	Open
	5	29.3	9.1	52%	Mixed
	4	29.8	10.6	69%	Grass
	3b	29.7	11.0	63%	Mixed
	2a	29.7	10.3	66%	Grass
	1b	29.6	7.2	77%	Grass
2	7	30.5	9.6	101%	Grass
	5	29.3	6.6	17%	Deciduous
	4	29.5	9.2	62%	Mixed
	2a	29.7	4.7	87%	Grass
	1b	29.1	5.3	62%	Mixed
3	4a	30.1	12.1	90%	Grass
	3b	29.2	8.1	50%	Mixed
	3a	29.8	10.8	73%	Grass
	2b	29.2	7.8	18%	Deciduous
	2a	30.1	8.2	92%	Grass
	1	29.9	8.1	94%	Grass

Table 18: Results of *n*-alkane quantification from sedimentary units in Trenches 1, 2 and 3 at Kalavan-2

References:

1. Volodicheva N. The physical geography of northern Eurasia. The physical geography of northern Eurasia. Oxford: Oxford University Press; 2002. pp. 350–376.

2. Kuhn SL, Raichlen DA, Clark AE. What moves us? How mobility and movement are at the center of human evolution. Evol Anthropol Issues, News, Rev. 2016;25: 86–97. doi:10.1002/evan.21480

3. Hovers E. Middle Paleolithic open-air sites. In: Enzel, Y., Bar-Yosef O, editor. Quaternary of the Levant Environments, Climate Change, and Humans. Cambridge: Cambridge University Press; 2017. pp. 593–600.

4. Wegwerth A, Ganopolski A, Ménot G, Kaiser J, Dellwig O, Bard E, et al. Black Sea temperature response to glacial millennial- scale climate variability. Geophys Res Lett. 2015;42: 8147–8154. doi:10.1002/2015GL065499

5. Djamali M, de Beaulieu J-L, Shah-hosseini M, Andrieu-Ponel V, Ponel P, Amini A, et al. A late Pleistocene long pollen record from Lake Urmia, Nw Iran. Quat Res. 2008;69: 413–420. doi:10.1016/j.yqres.2008.03.004

6. Randlett M-E, Bechtel A, van der Meer MTJ, Peterse F, Litt T, Pickarski N, et al. Biomarkers in Lake Van sediments reveal dry conditions in eastern Anatolia during 110.000-10.000 years B.P. Geochemistry, Geophys Geosystems. 2017;18: 571–583. doi:10.1002/2016GC006621

7. Pickarski N, Kwiecien O, Djamali M, Litt T. Vegetation and environmental changes during the last interglacial in eastern Anatolia (Turkey): A new high-resolution pollen record from Lake Van. Palaeogeogr Palaeoclimatol Palaeoecol. 2015;435: 145–158. doi:10.1016/j.palaeo.2015.06.015

8. Fleitmann D, Cheng H, Badertscher S, Edwards RL, Mudelsee M, Göktürk OM, et al. Timing and climatic impact of Greenland interstadials recorded in stalagmites from northern Turkey. Geophys Res Lett. 2009;36: L19707. doi:10.1029/2009GL040050

9. Ünal-İmer E, Shulmeister J, Zhao J-X, Tonguç Uysal I, Feng Y-X, Duc Nguyen A, et al. An 80 kyr-long continuous speleothem record from Dim Cave, SW

Turkey with paleoclimatic implications for the Eastern Mediterranean. Sci Rep. 2015;5: 13560. doi:10.1038/srep13560

10. Trigui Y, Wolf D, Sahakyan L, Hovakimyan H, Sahakyan K, Zech R, et al. First Calibration and Application of Leaf Wax n-Alkane Biomarkers in Loess-Paleosol Sequences and Modern Plants and Soils in Armenia. Geosciences. 2019;9: 263. doi:10.3390/geosciences9060263

11. Richter C, Wolf D, Walther F, Meng S, Sahakyan L, Hovakimyan H, et al. New insights into Southern Caucasian glacial–interglacial climate conditions inferred from Quaternary gastropod fauna. J Quat Sci. 2020; jqs.3204. doi:10.1002/jqs.3204

12. Wolf D, Baumgart P, Meszner S, Fülling A, Haubold F, Sahakyan L, et al. Loess in Armenia - stratigraphic findings and palaeoenvironmental indications. Proc Geol Assoc. 2016;127: 29–39. doi:10.1016/j.pgeola.2016.02.002

13. Bar-Yosef O, Belfer-Cohen A, Adler D. The implications of the Middle-Upper Paleolithic chronological boundary in the Caucasus to Eurasian prehistory. Anthropologie. 2006; 49–60. doi:10.1016/S0070-2153(07)00007-5

14. Ghukasyan, R., Colonge, D., Nahapetyan, S., Ollivier, V., Gasparyan, B., Monchot, H., Chataigner C. Kalavan-2 (North of Lake Sevan, Armenia): A new late middle paleolithic site in the Lesser Caucasus. Archaeol Ethnol Anthropol Eurasia. 2011;38: 39–51. doi:10.1016/j.aeae.2011.02.003

15. Kandel AW, Gasparyan B, Allué E, Bigga G, Bruch AA, Cullen VL, et al. The earliest evidence for Upper Paleolithic occupation in the Armenian Highlands at Aghitu-3 Cave. J Hum Evol. 2017;110: 37–68. doi:10.1016/j.jhevol.2017.05.010

16. Glauberman, P., Gasparyan, B., Wilkinson, K., Frahm, E., Raczynski-Henk, Y., Haydosyan, H., Arakelyan, D., Karapetyan, S., Nahapetyan, S. and Adler D. Introducing Barozh 12: A Middle Palaeolithic open-air site on the edge of the Ararat Depression, Armenia. Aram J Near East Stud. 2016;IX: 7–20.

17. Glauberman P, Gasparyan B, Sherriff J, Wilkinson K, Li B, Knul M, et al. Barozh 12: Formation processes of a late Middle Paleolithic open-air site in western Armenia. Quat Sci Rev. 2020;236: 106276.

doi:10.1016/j.quascirev.2020.106276

18. Pinhasi R, Gasparian B, Nahapetyan S, Bar-Oz G, Weissbrod L, Bruch AA, et al. Middle Palaeolithic human occupation of the high altitude region of Hovk-1, Armenia. Quat Sci Rev. 2011;30: 3846–3857. doi:10.1016/j.quascirev.2011.09.020

19. Gasparyan, B., Egeland, C.P., Adler, D.S., Pinhasi, R., Glauberman, P., Haydosyan H. The Middle Paleolithic occupation of Armenia: summarizing old and new data. In: Gasparyan, B., Arimura M, editor. Stone Age of Armenia: a Guide-book to the Atone Age Archaeology in the Republic of Armenia. Kanazawa: Kanazawa University Press; 2014. pp. 65–105.

20. Moncel M-H, Pleurdeau D, Pinhasi R, Yeshurun R, Agapishvili T, Chevalier T, et al. the Middle Palaeolithic Record of Georgia: a Synthesis of the Technological, Economic and Paleoanthropological Aspects. Anthropologie. 2015;LIII: 93–125.

21. Egeland CP, Gasparian B, Fadem CM, Nahapetyan S, Arakelyan D, Nicholson CM. Bagratashen 1, a stratified open-air Middle Paleolithic site in the Debed river valley of northeastern Armenia: A preliminary report. Archaeol Res Asia. 2016;8: 1–20. doi:10.1016/j.ara.2016.10.001

22. Pleurdeau D, Moncel MH, Pinhasi R, Yeshurun R, Higham T, Agapishvili T, et al. Bondi Cave and the Middle-Upper Palaeolithic transition in western Georgia (south Caucasus). Quat Sci Rev. 2016;146: 77–98. doi:10.1016/j.quascirev.2016.06.003

23. Frahm E, Feinberg JM, Schmidt-Magee BA, Wilkinson KN, Gasparyan B, Yeritsyan B, et al. Middle Palaeolithic toolstone procurement behaviors at Lusakert Cave 1, Hrazdan valley, Armenia. J Hum Evol. 2016;91: 73–92. doi:10.1016/j.jhevol.2015.10.008

24. Sherriff JE, Wilkinson KN, Adler DS, Arakelyan D, Beverly EJ, Blockley SPE, et al. Pleistocene volcanism and the geomorphological record of the Hrazdan valley, central Armenia: linking landscape dynamics and the Palaeolithic record. Quat Sci Rev. 2019;226: 105994. doi:10.1016/j.quascirev.2019.105994

25. Malinsky-Buller, A., Glauberman, P., Sherriff, J., Li, B., Frahm, E., Gasparyan,

B., Timms, R., Adler, D.S., Wilkinson K. Middle Palaeolithic occupation and environmental change in the Central Armenia: new evidence from the open-air site of Alapars-1. Quat Res. doi:10.1017/qua.2020.61

26. Zazanashvili, N., Mallon D. Status and Protection of Globally Threatened Species in the Caucasus. Tbilisi: CEPF Biodiversity Investments in the Caucasus Hotspot 2004-2009; 2009.

27. Stockhecke M, Timmermann A, Kipfer R, Haug GH, Kwiecien O, Friedrich T, et al. Millennial to orbital-scale variations of drought intensity in the Eastern Mediterranean. Quat Sci Rev. 2016;133: 77–95. doi:10.1016/j.quascirev.2015.12.016

28. Rowe PJ, Mason JE, Andrews JE, Marca AD, Thomas L, Van Calsteren P, et al. Speleothem isotopic evidence of winter rainfall variability in northeast Turkey between 77 and 6 ka. Quat Sci Rev. 2012;45: 60–72. doi:10.1016/j.quascirev.2012.04.013

29. Rowe PJ, Wickens LB, Sahy D, Marca AD, Peckover E, Noble S, et al. Multi-proxy speleothem record of climate instability during the early last interglacial in southern Turkey. Palaeogeogr Palaeoclimatol Palaeoecol. 2020;538: 109422. doi:10.1016/j.palaeo.2019.109422

30. Khorozyan, I.G., Weinberg, P.I., Malkhasyan G. Conservation strategy for Armenian mouflon (Ovis [orientalis] gmelini Blyth) and Bezoar goat (Capra aegagrus Erxleben) in Armenia. In: Zazanashvili, N., Mallon D, editor. Status and Protection of Globally Threatened Species in the Caucasus. Tiblisi: CEPF Biodiversity Investments in the Caucasus Hotspot 2004-2009; 2009. pp. 37–45.

31. Vereshchagin NK. The mammals of the Caucasus: A history of the evolution of the fauna. Jerusalem: Israel Program for Scientific Translations; 1967.

32. Heptner, V., Sludskii AA. Mammals of the Soviet Union. Washington DC: Smithsonian Institution Libraries and National Science Foundation; 1992.

33. Bleyhl B, Sipko T, Trepet S, Bragina E, Leitão PJ, Radeloff VC, et al. Mapping seasonal European bison habitat in the Caucasus Mountains to identify potential reintroduction sites. Biol Conserv. 2015;191: 83–92.

doi:10.1016/j.biocon.2015.06.011

34. Bleyhl B, Baumann M, Griffiths P, Heidelberg A, Manvelyan K, Radeloff VC, et al. Assessing landscape connectivity for large mammals in the Caucasus using Landsat 8 seasonal image composites. Remote Sens Environ. 2017;193: 193–203. doi:10.1016/j.rse.2017.03.001

35. Adler, D. S., Bar- Oz, G., Belfer- Cohen A, Bar- Yosef O. Ahead of the Game. Curr Anthropol. 2006;47: 89–118. doi:10.1086/432455

36. Tornero C, Balasse M, Bălăşescu A, Chataigner C, Gasparyan B, Montoya C. The altitudinal mobility of wild sheep at the Epigravettian site of Kalavan 1 (Lesser Caucasus, Armenia): Evidence from a sequential isotopic analysis in tooth enamel. J Hum Evol. 2016;97: 27–36. doi:10.1016/j.jhevol.2016.05.001

37. Binford LR. Willow Smoke and Dogs ' Tails : Hunter-Gatherer Settlement Systems and Archaeological Site Formation. Am Archaeol. 1980;45: 4–20.

38. Whallon R. Social networks and information: Non-"utilitarian" mobility among hunter-gatherers. J Anthropol Archaeol. 2006;25: 259–270. doi:10.1016/j.jaa.2005.11.004

39. Grove M. Hunter-gatherer movement patterns: Causes and constraints. J Anthropol Archaeol. 2009;28: 222–233. doi:10.1016/j.jaa.2009.01.003

40. Kelly RL. The Lifeways of Hunter-Gatherers: The Foraging Spectrum. Cambridg: Cambridge University Press; 2013.

41. Binford LR. Constructing Frames of Reference: An Analytical Method for Archaeological Theory Building Using Ethnographic and Environmental Data Sets. Berkeley: University of California Press; 2001.

42. Delagnes A, Rendu W. Shifts in Neandertal mobility, technology and subsistence strategies in western France. J Archaeol Sci. 2011;38: 1771–1783. doi:10.1016/j.jas.2011.04.007

43. Kark S. Ecotones and Ecological Gradients. Ecological Systems. New York, NY: Springer New York; 2013. pp. 147–160. doi:10.1007/978-1-4614-5755-8_9

44. Barton, C. M., Riel-Salvatore J. The formation of lithic assemblages. J Archaeol Sci. 2014;46: 334–352.

45. Clark GA, Barton CM. Lithics, landscapes & la Longue-durée – Curation & expediency as expressions of forager mobility. Quat Int. 2017;450. doi:10.1016/j.quaint.2016.08.002

46. Bicho N, Cascalheira J. Use of Lithic Assemblages for the Definition of Short-Term Occupations in Hunter-Gatherer Prehistory. 2020. pp. 19–38. doi:10.1007/978-3-030-27403-0_2

47. Binford LR. Bones: Ancient Men and Modern Myths. New York: Academic Press; 1981.

48. Binford LR. Long term land use patterns: some implications for archaeology. In: Dunnell, R.C., Grayson DK, editor. Lulu Linear Punctated: essays in honor of George Irving Quimby. Ann Arbor: University of Michiga: Museum of Anthropology:; 1983. pp. 27–53.

49. Speth J. Middle Palaeolithic subsistence in the Near East. Before Farming. 2014;2012: 1–45. doi:10.3828/bfarm.2012.2.1

50. Brunet O. Bronze and Iron Age carnelian bead production in the UAE and Armenia: new perspectives. Proc Semin Arab Stud. 2009;39: 57–68.

51. Strahler AN. Quantitative analysis of watershed geomorphology. Trans Am Geophys Union. 1957;38: 913–920.

52. Guo Q, Liu M, Luo L, Yan K, Guo W, Wu G, et al. Geochemical controls on magnesium and its speciation in various types of geothermal waters from typical felsic-rock-hosted hydrothermal systems in China. Geothermics. 2019;81: 185–197. doi:10.1016/j.geothermics.2019.05.006

53. Stoops G, Jongerius A. Proposal for a micromorphological classification of soil materials. I. A classification of the related distributions of fine and coarse particles. Geoderma. 1975;13: 189–199. doi:10.1016/0016-7061(75)90017-8

54. Goldberg P, Berna F. Micromorphology and context. Quat Int. 2010;214: 56–62. doi:10.1016/j.quaint.2009.10.023

55. Van Vliet-Lanoë B. Frost and soils: implications for paleosols, paleoclimates and stratigraphy. Catena. 1998;34: 157–183.

56. Aitken MJ. An introduction to optical dating: The dating of Quaternary

sediments by the use of photon-stimulated luminescence. Oxford: Oxford science publications; 1998.

57. Jones, A.P., Tucker, M.E., Hart J. The description & analysis of quaternary stratigraphic field sections, vol. 7,. London: Quaternary Research Association,Technical Guide, 7; 1999.

58. Lane, C.S., Cullen, V.L., White, D., Bramham-Law, C.W.F., Smith VC. Cryptotephra as a dating and correlation tool in archaeology. J Archaeol Sci. 2014;42: 42–50.

59. Blockley, S.P.E., Pyne-O'Donnell, S.D.F., Lowe, J.J., Matthews, I.P., Stone, A., Pollard, A.M., Turney, C.S.M. and Molyneux EG. A new and less destructive laboratory procedure for the physical separation of distal glass tephra shards from sediments. Quat Sci Rev. 2005;24: 1952–1960.

60. Hayward C. High spatial resolution electron probe microanalysis of tephras and melt inclusions without beam-induced chemical modification. The Holocene. 2012;22: 119–125. Available: 119-125

61. Thomsen KJ, Murray AS, Jain M, Bøtter-Jensen L. Laboratory fading rates of various luminescence signals from feldspar-rich sediment extracts. Radiat Meas. 2008;43: 1474–1486. doi:10.1016/j.radmeas.2008.06.002

62. Buylaert JP, Murray AS, Thomsen KJ, Jain M. Testing the potential of an elevated temperature IRSL signal from K-feldspar. Radiat Meas. 2009;44: 560–565. doi:10.1016/j.radmeas.2009.02.007

63. Arutyunyan E V., Lebedev VA, Chernyshev I V., Sagatelyan AK. Geochronology of Neogene-Quaternary volcanism of the Geghama Highland (Lesser Caucasus, Armenia). Dokl Earth Sci. 2007;416: 1042–1046. doi:10.1134/s1028334x07070136

64. Lebedev VA, Chernyshev I V., Yakushev AI. Initial time and duration of quaternary magmatism in the Aragats neovolcanic area (Lesser Caucasus, Armenia). Dokl Earth Sci. 2011;437: 532–536. doi:10.1134/s1028334x11040209

65. Lebedev VA, Chernyshev I V., Shatagin KN, Bubnov SN, Yakushev AI. The

quaternary volcanic rocks of the Geghama highland, Lesser Caucasus, Armenia: Geochronology, isotopic Sr-Nd characteristics, and origin. J Volcanol Seismol. 2013;7: 204–229. doi:10.1134/S0742046313030044

66. Cullen, V.L., Smith, V.C., Arz HW. The detailed tephrostratigraphy of a core from the south- east Black Sea spanning the last~ 60 ka. J Quat Sci. 2014;29.

67. Pearce, J.A., Bender, J.F., De Long, S.E., Kidd, W.S.F., Low, P.J., Güner, Y., Saroglu, F., Yilmaz, Y., Moorbath, S., Mitchell JG. Genesis of collision volcanism in Eastern Anatolia, Turkey. J Volcanol Geotherm Res. 1990;44: 189–229.

68. Şengör, A.M.C., Özeren, S., Genç, T., Zor E. East Anatolian high plateau as a mantle- supported, north- south shortened domal structure. Geophys Res Lett. 2003;30.

69. Keskin M. Eastern Anatolia: a hotspot in a collision zone without a mantle plume. Geol Soc Am. 2007;430: 693.

70. Yılmaz, Y., Güner, Y., Şaroğlu F. Geology of the Quaternary volcanic centres of the East Anatolia. J Volcanol Geotherm Res. 1998;85: 173–210.

71. Sumita, M., Schmincke H. Impact of volcanism on the evolution of Lake Van II: temporal evolution of explosive volcanism of Nemrut Volcano (eastern Anatolia) during the past ca. 0.4 Ma. J Volcanol Geotherm Res. 2013;253: 15–34.

72. Macdonald, R., Sumita, M., Schmincke, H. U. Bagiński, B. White JC, Ilnicki SS. Peralkaline felsic magmatism at the Nemrut volcano, Turkey: impact of volcanism on the evolution of Lake Van (Anatolia) IV. Contrib to Mineral Petrol. 2015;169: 1–22. doi:10.1007/s00410-015-1127-6

73. Çubukçu, H. E. Ulusoy, I. Aydar, E. Ersoy, O. Şen, E. Gourgaud, A. Guillou H. Mt. Nemrut volcano (Eastern Turkey): Temporal petrological evolution. J Volcanol Geotherm Res. 2012;209–210: 33–60. doi:10.1016/j.jvolgeores.2011.08.005

74. Sumita M, Schmincke H-U. Impact of volcanism on the evolution of Lake Van I: evolution of explosive volcanism of Nemrut Volcano (eastern Anatolia)

during the past 400,000 years. Bull Volcanol. 2013;75: 714. doi:10.1007/s00445-013-0714-5

75. Schmincke HU, Sumita M. Impact of volcanism on the evolution of Lake Van (eastern Anatolia) III: Periodic (Nemrut) vs. episodic (Süphan) explosive eruptions and climate forcing reflected in a tephra gap between ca. 14 ka and ca. 30 ka. J Volcanol Geotherm Res. 2014;285: 195–213. doi:10.1016/j.jvolgeores.2014.08.015

76. Lebedev, V.A., Chugaev, A.V., Ünal, E., Sharkov, E.V., Keskin M. Late Pleistocene Tendürek Volcano (Eastern Anatolia, Turkey). II. Geochemistry and petrogenesis of the rocks. Petrology. 2016;24: 234–270.

77. Landmann, G., Steinhauser, G., Sterba, J.H., Kempe, S., Bichler M. Geochemical fingerprints by activation analysis of tephra layers in Lake Van sediments, Turkey. Appl Radiat Isot. 2011;69: 929–935.

78. Özdemir, Y., Güleç N. Geological and Geochemical Evolution of the Quaternary Suphan Stratovolcano, Eastern Anatolia, Turkey: Evidence for the Lithosphere-Asthenosphere Interaction in Post-Collisional Volcanism. J Petrol. 2013;55: 37–62.

79. Lebedev, V.A., Sharkov, E.V., Ünal, E., Keskin M. Late Pleistocene Tendürek volcano (Eastern Anatolia, Turkey): I. Geochronology and petrographic characteristics of igneous rocks. Petrology. 2016;24: 127–152.

80. Albert, P.G., Tomlinson, E.L., Smith, V.C., Di Traglia, F., Pistolesi, M., Morris, A., Donato, P., De Rosa, R., Sulpizio, R., Keller, J. and Rosi M. Glass geochemistry of pyroclastic deposits from the Aeolian Islands in the last 50 ka: A proximal database for tephrochronology. J Volcanol Geotherm Res. 2017;336: 81–107.

81. Szakács, A., Seghedi, I., Pécskay, Z., Mirea V. Eruptive history of a low-frequency and low-output rate Pleistocene volcano, Ciomadul, South Harghita Mts., Romania. Bull Volcanol. 2015;77: 12.

82. Karátson, D., Wulf, S., Veres, D., Magyari, E.K., Gertisser, R., Timar-Gabor, A., Novothny, Á., Telbisz, T., Szalai, Z., Anechitei-Deacu, V., Appelt, O. 2016. 319, 29-51.Karátson, D., Wulf, S., Veres, D., Magyari, E.K., Gertisser, R., Timar-

Gabor A. The latest explosive eruptions of Ciomadul (Csomád) volcano, East Carpathians—a tephrostratigraphic approach for the 51–29 ka BP time interval. J Volcanol Geotherm Res. 2016;319: 29–51.

83. Wulf, S., Fedorowicz, S., Veres, D., Łanczont, M., Karátson, D., Gertisser, R., Bormann, M., Magyari, E., Appelt, O., Hambach, U., Gozhyk PF. The 'Roxolany Tephra' (Ukraine)– new evidence for an origin from Ciomadul volcano, East Carpathians. J Quat Sci. 2016;31: 565–576.

84. Hamann Y, Wulf S, Ersoy O, Ehrmann W, Aydar E, Schmiedl G. First evidence of a distal early Holocene ash layer in Eastern Mediterranean deep-sea sediments derived from the anatolian volcanic province. Quat Res. 2010;73: 497–506. doi:10.1016/j.yqres.2009.12.004

85. Druitt, T.H., Brenchley, P.J., Gökten, Y.E., Francaviglia V. Late Quaternary rhyolitic eruptions from the Acigöl Complex, central Turkey. J Geol Soc London. 1995;152: 655–667.

86. Deniel, C., Aydar, E., Gourgaud A. The Hasan Dagi stratovolcano (Central Anatolia, Turkey): evolution from calc-alkaline to alkaline magmatism in a collision zone. J Volcanol Geotherm Res. 1998;87: 275–302.

87. Kuzucuoglu, C., Pastre, J.F., Black, S., Ercan, T., Fontugne, M., Guillou, H., Hatté, C., Karabiyikoglu, M., Orth, P. and Türkecan A. Identification and dating of tephra layers from Quaternary sedimentary sequences of Inner Anatolia, Turkey. J Volcanol Geotherm Res. 1998;85: 153–172.

88. Slimak, L., Kuhn, S.L., Roche, H., Mouralis, D., Buitenhuis, H., Balkan-Atlı, N., Binder, D., Kuzucuoğlu, C., Guillou H. aletepe Deresi 3 (Turkey): Archaeological evidence for early human settlement in Central Anatolia. J Hum Evol. 2008;54: 99–111.

89. Tryon, C. A., Logan, M. A. V., Mouralis, D., Kuhn, S., Slimak, L., Balkan-Atlı N. Building a tephrostratigraphic framework for the Paleolithic of central Anatolia, Turkey. J Archaeol Sci. 2009;36: 637–652.

90. Schmitt, A.K., Danišík, M., Evans, N.J., Siebel, W., Kiemele, E., Aydin, F., Harvey JC. Acigöl rhyolite field, Central Anatolia (part 1): high-resolution dating of eruption episodes and zircon growth rates. Contrib to Mineral Petrol.

2011;162: 1215–1231.

91. Bordes F. Typologie du Paléolithique Ancien et Moyen. Paris: CNRS; 1961.

92. Pelegrin J. Les techniques de de´bitage laminaire au Tardiglaciaire : crite`res de diagnose et quelques re´flexions. In: Valentin, B., Bodu, P., Christensen M, editor. L'Europe centrale et septentrionale au Tardiglaciaire,. Nemours: Me´moires du Muse´e de Pre´histoire d'Ile de France 7; 2000. pp. 73–86.

93. Hurcombe L. Use wear analysis and obsidian: theory, experiments and results. Sheffield. Sheffield: Sheffield: JR Collis Publications; 1992.

94. Walton DP. An Experimental Program for Obsidian Use-Wear Analysis in Central Mexican Archaeology. J Archaeol Method Theory. 2018. doi:10.1007/s10816-018-9398-7

95. Vergès JM, Ollé A. Technical microwear and residues in identifying bipolar knapping on an anvil: experimental data. J Archaeol Sci. 2011;38: 1016–1025. doi:10.1016/j.jas.2010.11.016

96. Dubreuil L, Savage D. Ground stones: a synthesis of the use-wear approach. J Archaeol Sci. 2014;48: 139–153. doi:10.1016/j.jas.2013.06.023

97. Goren-Inbar N, Sharon G, Alperson-Afil N, Herzlinger G. A new type of anvil in the Acheulian of Gesher Benot Ya'aqov, Israel. Philos Trans R Soc B Biol Sci. 2015;370: 20140353. doi:10.1098/rstb.2014.0353

98. Revedin A, Aranguren B, Becattini R, Longo L, Marconi E, Lippi MM, et al. Thirty thousand-year-old evidence of plant food processing. Proc Natl Acad Sci. 2010;107: 18815–18819. doi:10.1073/pnas.1006993107

99. Oron M, Goren-Inbar N. Mousterian intra-site spatial patterning at Quneitra, Golan Heights. Quat Int. 2014;331: 186–202. doi:10.1016/j.quaint.2013.04.013

100. Beaune de S. Pour une archéologie du geste Broyer, moudre, piler, des premiers chasseurs aux premiers agriculteurs. Paris: CNRS; 2000.

101. Zaidner Y. Adaptive Flexibility of Oldowan Hominins: Secondary Use of Flakes at Bizat Ruhama, Israel. Petraglia MD, editor. PLoS One. 2013;8: e66851. doi:10.1371/journal.pone.0066851

102. Frahm E, Tryon CA. Later Stone Age toolstone acquisition in the Central Rift Valley of Kenya: Portable XRF of Eburran obsidian artifacts from Leakey's excavations at Gamble's Cave II. J Archaeol Sci Reports. 2018;18: 475–486. doi:10.1016/j.jasrep.2018.01.042

103. Frahm E, Brody LR. Origins of obsidian at the "Pompeii of the Syrian Desert:" Sourcing lithic artifacts from the Yale-French excavations at Dura-Europos. J Archaeol Sci Reports. 2019;24: 608–622. doi:10.1016/j.jasrep.2019.02.024

104. Frahm E. Can I get chips with that? Sourcing small obsidian artifacts down to microdebitage scales with portable XRF. J Archaeol Sci Reports. 2016;9: 448–467. doi:10.1016/j.jasrep.2016.08.032

105. Lyman RL. Quantitative Paleozoology. Cambridge: Cambridge University Press; 2008. doi:10.1017/CBO9780511813863

106. Kindler, L., Smith, G.M., Garcia-Moreno, A., Gaudzinski-Windheuser, S., Pop, E., Roebroeks W. The last interglacial (Eemian) lakeland of Neumark-Nord (Saxony-Anhalt, Germany). Sequencing Neanderthal occupations, assessing subsistence opportunities and prey selection based on estimations of ungulate carrying capacities, biomass production and energ. In: Garcia-Moreno, A., Hutson, J.M., Smith, G.M., Kindler, L., Turner, E., Villaluenga, A., Gaudzinski-Windheuser S, editor. Human Behavioural Adaptations to Interglacial Lakeshore Environments. Mainz and Heidelberg: RGZM-Tagungen; 2020. pp. 67–104.

107. Smith, F.A., Lyons, S.K., Ernest, S.K.M., Jones, K.E., Kaufman, D.M., Dayan, T., Marquet, P.A., Brown, J.H., Haskell JP. Body Mass of Late Quaternary Mammals. Ecology. 2003;84: 3403–3403.

108. Shipman P, Foster G, Schoeninger M. Burnt bones and teeth: an experimental study of color, morphology, crystal structure and shrinkage. J Archaeol Sci. 1984;11: 307–325. doi:10.1016/0305-4403(84)90013-X

109. Stiner MC, Kuhn SL, Weiner S, Bar-Yosef O. Differential Burning, Recrystallization, and Fragmentation of Archaeological Bone. J Archaeol Sci. 1995;22: 223–237. doi:10.1006/jasc.1995.0024

110. Reidsma FH, van Hoesel A, van Os BJH, Megens L, Braadbaart F. Charred

bone: Physical and chemical changes during laboratory simulated heating under reducing conditions and its relevance for the study of fire use in archaeology. J Archaeol Sci Reports. 2016;10: 282–292. doi:10.1016/j.jasrep.2016.10.001

111. Sánchez-Hernández C, Rivals F, Blasco R, Rosell J. Short, but repeated Neanderthal visits to Teixoneres Cave (MIS 3, Barcelona, Spain): a combined analysis of tooth microwear patterns and seasonality. J Archaeol Sci. 2014;49: 317–325. doi:10.1016/j.jas.2014.06.002

112. Sánchez-Hernández C, Rivals F, Blasco R, Rosell J. Tale of two timescales: Combining tooth wear methods with different temporal resolutions to detect seasonality of Palaeolithic hominin occupational patterns. J Archaeol Sci Reports. 2016;6: 790–797. doi:10.1016/j.jasrep.2015.09.011

113. Andrews P CJ. Owls, caves and fossils: predation, preservation and accumulation of small mammal bones in caves, with an analysis of the Pleistocene cave faunas from Westbury-sub-Mendip, Somerset, UK. University of Chicago Press; 1990.

114. Beatty MD, Hillemann HH. Osteogenesis in the Golden Hamster. J Mammal. 1950;31: 121. doi:10.2307/1375503

115. Tipping R, Bunting MJ, Davies AL, Murray H, Fraser S, McCulloch R. Modelling land use around an early Neolithic timber ‘hall’ in north east Scotland from high spatial resolution pollen analyses. J Archaeol Sci. 2009;36: 140–149. doi:10.1016/j.jas.2008.07.016

116. Bliedtner M, Schäfer IK, Zech R, Von Suchodoletz H. Leaf wax n-alkanes in modern plants and topsoils from eastern Georgia (Caucasus) - Implications for reconstructing regional paleovegetation. Biogeosciences. 2018;15: 3927–3936. doi:10.5194/bg-15-3927-2018

117. Bliedtner M, von Suchodoletz H, Schäfer I, Welte C, Salazar G, Szidat S, et al. Age and origin of leaf wax <i>n</i>-alkanes in fluvial sediment–paleosol sequences and implications for paleoenvironmental reconstructions. Hydrol Earth Syst Sci. 2020;24: 2105–2120. doi:10.5194/hess-24-2105-2020

118. Nowak, R. M., Paradiso, J. L. W. Walker's mammals of the world. 5th ed. Press. JHU, editor. Baltimore:; 1991.

119. Lyman RL. Paleoenvironmental Reconstruction from Faunal Remains: Ecological Basics and Analytical Assumptions. J Archaeol Res. 2017;25: 315–371. doi:10.1007/s10814-017-9102-6

120. Feoktistova, N.Y., Meschersky, I.G., Bogomolov, P.L., Sayan, A.S., Poplavskaya, Surov NS. Phylogeographic structure of the Common hamster (Cricetus cricetus L.): Late Pleistocene connections between Caucasus and Western European populations populations. PLoS One. 2017;12(11): e0. doi:Https://doi.org/10.1371/journal., E0187527. Pone.0187527

121. Pinhasi, R., Gasparyan, B., Nahapetyan, S., Bar-Oz, G., Weissbrod, L., Bruch, A., Hovsepyan, R. and Wilkinson K. Middle Palaeolithic occupation at Hovk-1, Armenia. In: Avetisyan, P., Bobokhyan A, editor. Archaeology of Armenia in Regional Context, Proceedings of the International Conference dedicated to the 50th Anniversary of the Institute of Archaeology and Ethnography Held on September 15–17, 2009 in Yerevan. Yerevan: National Academy of Sciences of the Republic of Armenia "Gitutyun" Publishing House,; 2012. p. 38–51.

122. Kryštufek B, Hoffmann IE, Nedyalkov N, Pozdnyakov A V V. Cricetus cricetus (Rodentia: Cricetidae). Mamm Species. 2020;52: 10–26.

123. Yiğit, N., Colak, E., Gattermann, R., Neumann, K., Ozkurt, S., Gharakheloom, M.M. et al. Morphological and biometrical comparisons of Mesocricetus Nehring, 1898 (Mammalia: Rodentia) species distributed in the Palaearctic Region. Turkish J Zool. 2006;30: 291–299.

124. Tesakov, A.S., Тесаков АС. Early Middle Pleistocene Ellobius (Rodentia , Cricetidae , Arvicolinae) from Armenia С лепушонка Ellobius (Rodentia , Cricetidae , Arvicolinae). начала среднего плейстоцена Армении. 2016;15: 151–158.

125. Armitage K. Climate change and the conservation of marmots. 2013.

126. Jacob J, Manson P, Barfknecht R FT. Common vole (Microtus arvalis) ecology and management: implications for risk assessment of plant protection products. Pest Manag Sci. 2014;70: 869–878.

127. Mate I, Barrull J, Salicrú M, Ruiz-Olmo J GJ. Habitat selection by Southern water vole (Arvicola sapidus) in riparian environments of Mediterranean mountain areas: a conservation tool for the species. Acta Theriol. 2013;58: 25–37.

128. Borowski Z. Habitat selection and home range size of field voles Microtus agrestis in Słowiński National Park, Poland. Acta Theriol. 2003;48: 325–333.

129. Dokulilová M SJ. Abundance of common shrew (Sorex araneus) in selected forest habitats of Moravia (Czech Republic). Acta Univ Agric Silv Mendelianae Brun. 2017;65: 401–409.

130. Ollivier V, Fontugne M, Lyonnet B. Geomorphic response and 14C chronology of base-level changes induced by Late Quaternary Caspian Sea mobility (middle Kura Valley, Azerbaijan). Geomorphology. 2015;230: 109–124. doi:10.1016/j.geomorph.2014.11.010

131. Hovers E, Ekshtain R, Greenbaum N, Malinsky-Buller A, Nir N, Yeshurun R. Islands in a stream? Reconstructing site formation processes in the late Middle Paleolithic site of 'Ein Qashish, northern Israel. Quat Int. 2014;331. doi:10.1016/j.quaint.2014.01.028

132. Lam YM, Pearson OM. Bone density studies and the interpretation of the faunal record. Evol Anthropol Issues, News, Rev. 2005;14: 99–108. doi:10.1002/evan.20053

133. Lyman RL. Vertebrate Taphonomy. Cambridge University Press; 1994. doi:10.1017/CBO9781139878302

134. Domínguez-Rodrigo M, Uribelarrea D, Santonja M, Bunn HT, García-Pérez A, Pérez-González A, et al. Autochthonous anisotropy of archaeological materials by the action of water: experimental and archaeological reassessment of the orientation patterns at the Olduvai sites. J Archaeol Sci. 2014;41: 44–68. doi:10.1016/j.jas.2013.07.025

135. Binford LR. Nunamiut ethnoarchaeology. New York, NY: Academic Press; 1978.

136. Schick DK. Stone Age Sites in the Making: Experiments in the Formation and

Transformation of Archaeological Occurrences. Oxford: BAR 319, International Series; 1986.

137. Bertran P, Lenoble A, Todisco D, Desrosiers PM, Sørensen M. Particle size distribution of lithic assemblages and taphonomy of Palaeolithic sites. J Archaeol Sci. 2012;39: 3148–3166. doi:10.1016/j.jas.2012.04.055

138. Chu W, Hosfield R. Lithic artifact assemblage transport and microwear modification in a fluvial setting: A radio frequency identification tag experiment. Geoarchaeology. 2020; gea.21788. doi:10.1002/gea.21788

139. Kuhn SL. Mousterian Lithic Technology: An Ecological Perspective. Princeton: Princeton University Press; 1995.

140. Turq A, Roebroeks W, Bourguignon L, Faivre J-P. The fragmented character of Middle Palaeolithic stone tool technology. J Hum Evol. 2013;65: 641–655. doi:10.1016/j.jhevol.2013.07.014

141. Gallagher JP. Contemporary Stone Tools in Ethiopia: Implications for Archaeology. J F Archaeol. 1977;4: 407. doi:10.2307/529396

142. Bamforth DB. Technological efficiency and tool curation. Am Antiq. 1986;51: 38–50.

143. Shott MJ. On Tool-Class Use Lives and the Formation of Archaeological Assemblages. Am Antiq. 1989;54: 9–30. doi:10.2307/281329

144. Shott MJ. An exegesis of the curation concept. J Anthropol Res. 1996;52: 259–280.

145. Yeritsyan BG. Nekotoriye ossobennosti namerennogo rassecheniya orudiy must'erskoy epokhi. Nauka" Pub. In: Kruglikova IT, editor. Kratkiye soobshcheniya Instituta Arkheologii. Nauka" Pub. Moskow; 1972. p. 53–60.

146. Yeritsyan, S.A., Semyonov BG. Novaya nizhnepaleoliticheskaya peshchera Yerevan /New Lower Paleolithic cave of Yerevan. Kratkiye soobshcheniya Instituta Arkheologii /Briefs of the Institute of Archaeology/, N126, Paleolit i Neolit /Paleolithic and Neolithic/. Moskow: "Nauka" Publishing; 1971.

147. Wadley, L., Kempson H. A review of rock studies for archaeologists, and an analysis of dolerite and hornfels from the Sibudu area, KwaZulu-Natal. South

African Humanit. 2011;23: 65–85.

148. Vita-Finzi, C., Higgs ES. Prehistoric economy in the Mount Carmel area of Palestine: site catchment analysis. Proc Prehist Soc. 1970;36: 1–37.

149. Bailey, G.N., Davidson I. Site exploitation of territories and topography: two case studies from paleolithic Spain. J Archaeol Sci. 1983;10: 87-116.

150. Tobler W. Non-isotropic Geographic Modeling. Three Presentations on Geographic Analysis and Modeling. National Center for Geogrphic Information and analysis. University of California, Santa Barbara. Technical Report: National Center for Geogrphic Information and analysis. University of California, Santa Barbara. Technical R eport; 1993.

151. Glauberman, P., Gasparyan, B., Wilkinson, K., Frahm, E., Nahapetyan, S., Arakelyan, D., Raczynski-Henk, Y., Haydosyan, H., Adler D. Late Middle Paleolithic technological organization and behavior at the open-air site of Barozh 12 (Armenia). J Paleolit Archaeol. 2020.

152. Le Bourdonnec FX, Nomade S, Poupeau G, Guillou H, Tushabramishvili N, Moncel MH, et al. Multiple origins of Bondi Cave and Ortvale Klde (NW Georgia) obsidians and human mobility in Transcaucasia during the Middle and Upper Palaeolithic. J Archaeol Sci. 2012;39: 1317–1330. doi:10.1016/j.jas.2011.12.008

153. Adler DS. Late Middle Palaeolithic Patterns of Lithic Reduction, Mobility, and Land Use in the Southern Caucasus. Boston: Harvard University; 2002.

154. Centi L, Zaidner Y. Variations in lithic artefact density as a tool for better understanding Middle Palaeolithic human behaviour: The case of Nesher Ramla (Israel). Quat Int. 2020. doi:10.1016/j.quaint.2020.03.025

155. Malinslky-Buller A, Ekshtain, R., Munro, N., Hovers E. Back to base: Re-thinking variations in settlement and mobility beahviors in the Levantine Late Middle Paleolithic as seen from Shovakh Cave. J Archaeol Anthropol Sci.

156. Bertacchi A, Gasparyan B, Gruwier B, Rivals F, Kandel AW. Upper Paleolithic animal exploitation in the Armenian Highlands: The zooarchaeology of Aghitu-3 cave. Quat Int. 2020. doi:10.1016/j.quaint.2020.04.029

157. Bar-oz G, Adler DS. Taphonomic History of the Middle and Upper Palaeolithic Faunal Assemblage from Ortvale Klde , Georgian Republic. J Taphon. 2005;3: 185–211. Available: http://www.getcited.org/pub/103424157

158. Adler, D.S., Yeritsyan, B., Wilkinson, K., Pinhasi, R., Bar-Oz, G., Nahapetyan, S., Bailey, R., Schmidt, B.A., Glauberman, P., Wales, N. and Gasparian B. The Hrazdan Gorge Palaeolithic Project, 2008-2009. In: Avetisyan, P., Bobokhyan A, editor. Archaeology of Armenia in Regional Context. Yerevan: NAS RA Gitutyn Publishing House; 2012. pp. 21–37.

159. Brittingham A, Hren MT, Hartman G, Wilkinson KN, Mallol C, Gasparyan B, et al. Geochemical Evidence for the Control of Fire by Middle Palaeolithic Hominins. Sci Rep. 2019;9. doi:10.1038/s41598-019-51433-0

160. Montoya C, Balasescu A, Joannin S, Ollivier V, Liagre J, Nahapetyan S, et al. The Upper Palaeolithic site of Kalavan 1 (Armenia): An Epigravettian settlement in the Lesser Caucasus. J Hum Evol. 2013;65: 621–640. doi:10.1016/j.jhevol.2013.07.011

161. Bălăşescu, A., Montoya, C., Gasparyan, B., Liagre, J., Chataigner C. The Upper Palaeolithic fauna from Kalavan 1 (Armenia): preliminary results. Archaeozoology of the Near East 88. 2017.

162. Ekshtain R, Malinsky-Buller A, Ilani S, Segal I, Hovers E. Raw material exploitation around the middle paleolithic site of 'EinQashish. Quat Int. 2014;331. doi:10.1016/j.quaint.2013.07.025

163. Ekshtain, R. Ilani, S., Segal, I., Hovers E. Local and Nonlocal Procurement of Raw Material in Amud Cave, Israel: The Complex Mobility of Late Middle Paleolithic Groups. Geoarchaeology. 2017;32: 189–214. doi:10.1002/gea.21585

164. Turq A, Faivre JP, Gravina B, Bourguignon L. Building models of Neanderthal territories from raw material transports in the Aquitaine Basin (southwestern France). Quat Int. 2017;433. doi:10.1016/j.quaint.2016.02.062

165. Ekshtain, R., Malinsky-Buller, A., Greenbaum, N., Mitki, N., Stahlschmidt, C., Shahack-gross R, Nir, N., Porat, N. Bar-Yosef-Mayer, D. E. Yeshurun, R. Been, E. Rak, Y. Agha, N., Brailovsky, L., Krakovsky, M., Spivak, P., Ullman, M., Vered, A. Barzilai, O., Hovers E. Persistent Neanderthal occupation of the

open-air site of ‘ Ein Qashish , Israel. 2019. doi:10.17605/OSF.IO/AMGQ7

166. Gómez de Soler B, Soto M, Vallverdú J, Vaquero M, Bargalló A, Chacón MG, et al. Neanderthal lithic procurement and mobility patterns through a multi-level study in the Abric Romaní site (Capellades, Spain). Quat Sci Rev. 2020;237: 106315. doi:10.1016/j.quascirev.2020.106315

Figure

Click here to access/download;Figure;Kalavan 2 Fig. 1 Armenia maps.jpg

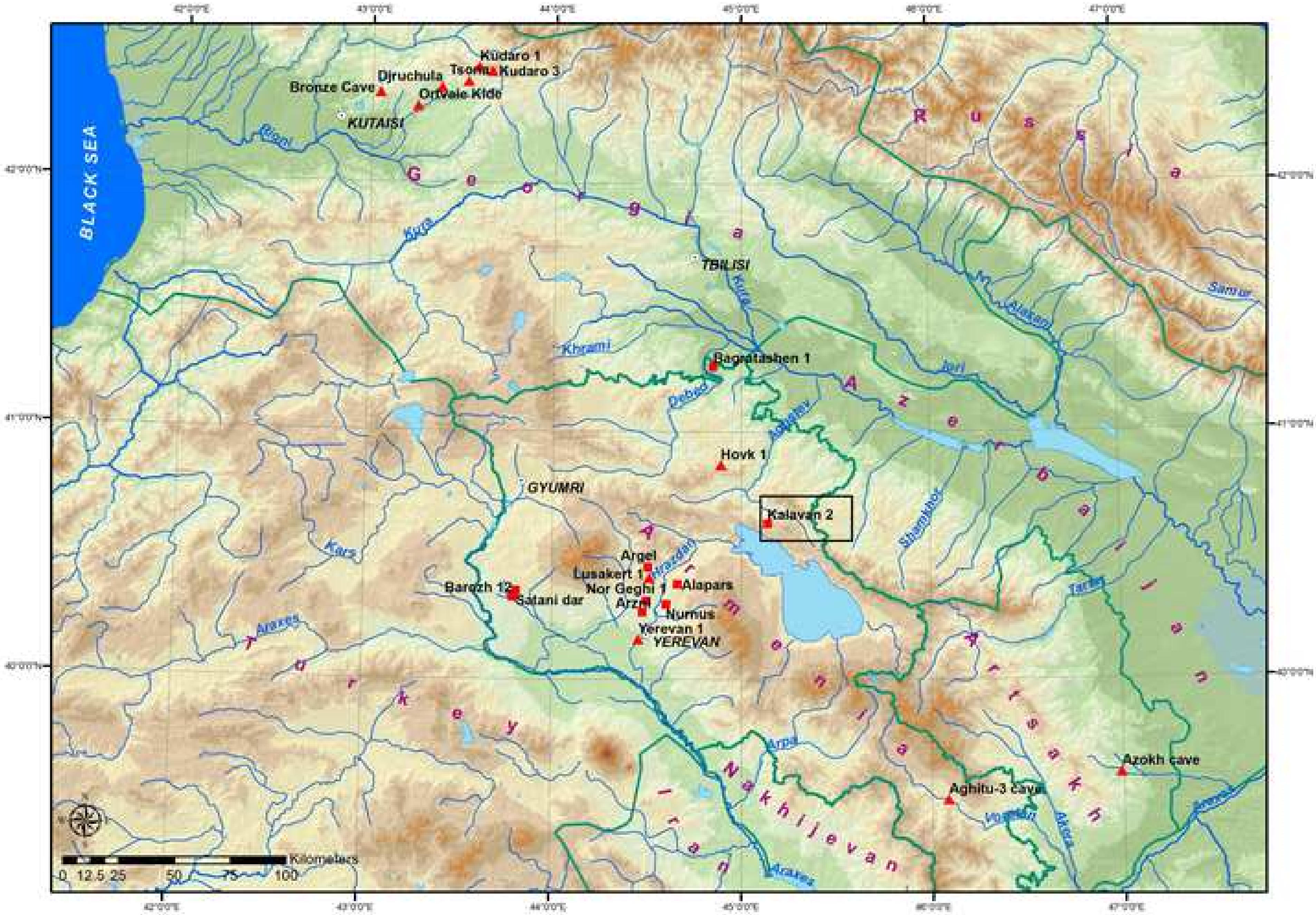

Figure Click here to access/download;Figure;Kalavan 2 Fig. 2.jpg

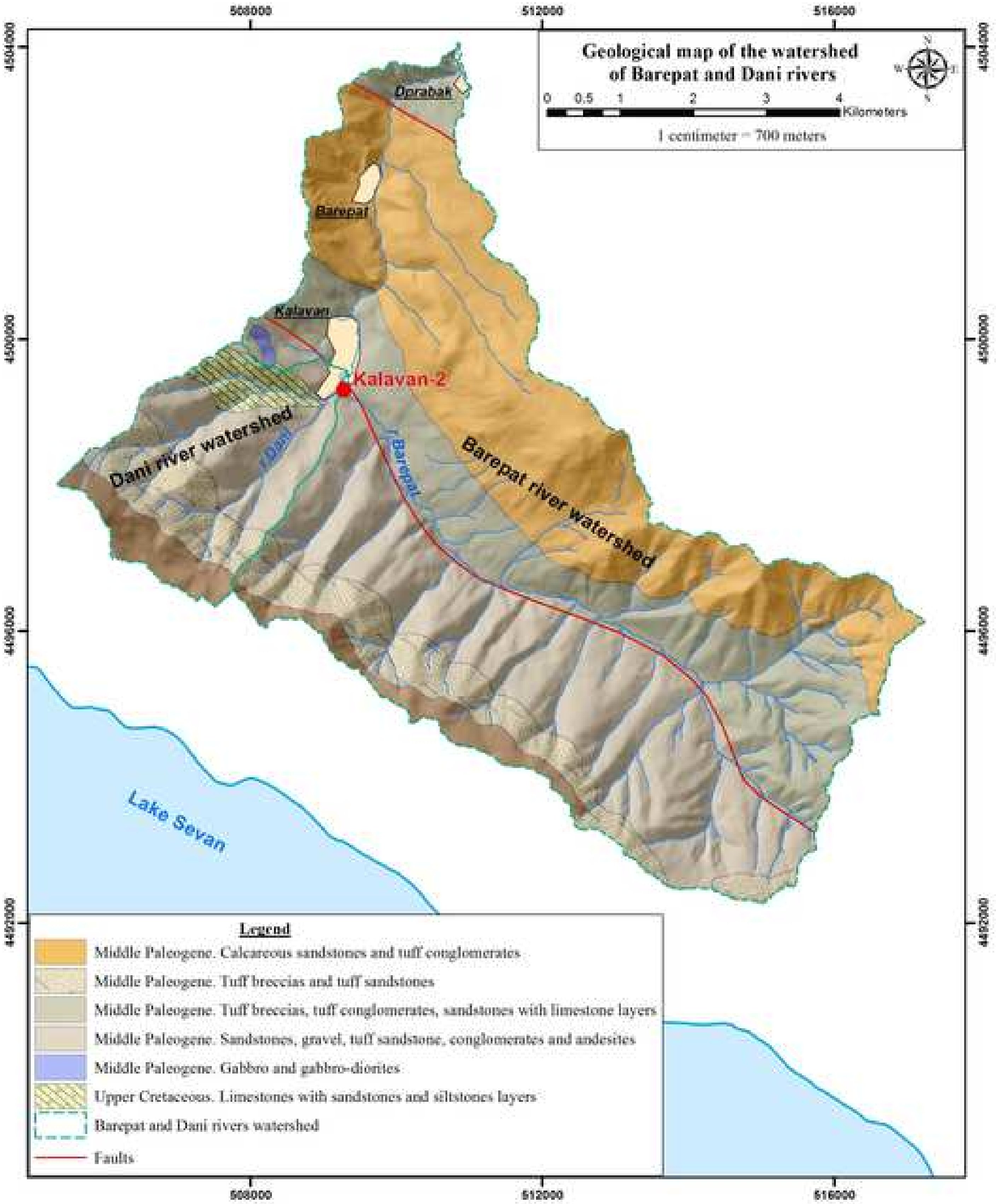

Figure

Click here to access/download;Figure;Kalavan 2 Fig. 3 Location of trenches.tif

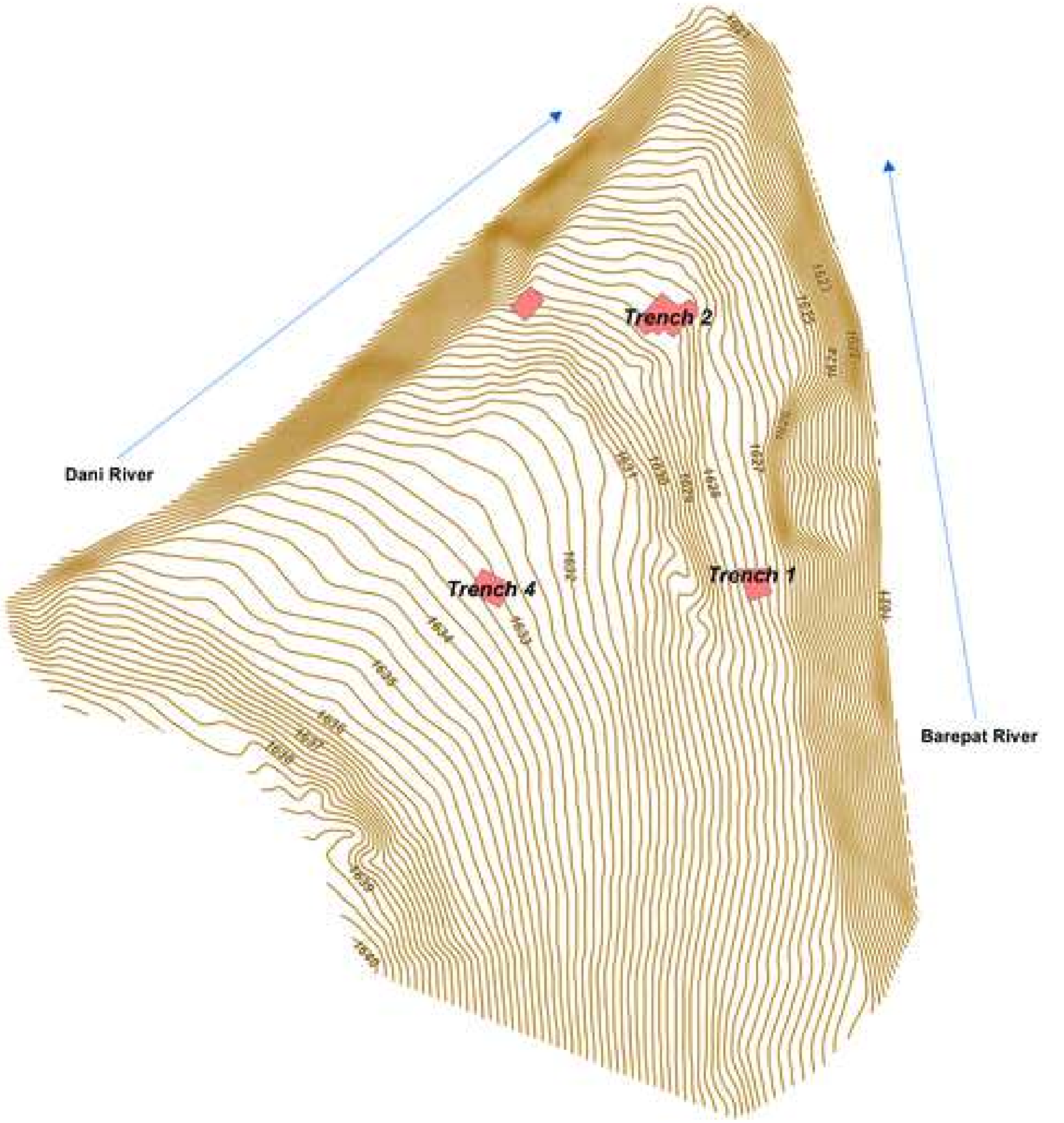

Figure

Click here to access/download;Figure;Kalavan 2 Fig. 4.jpg

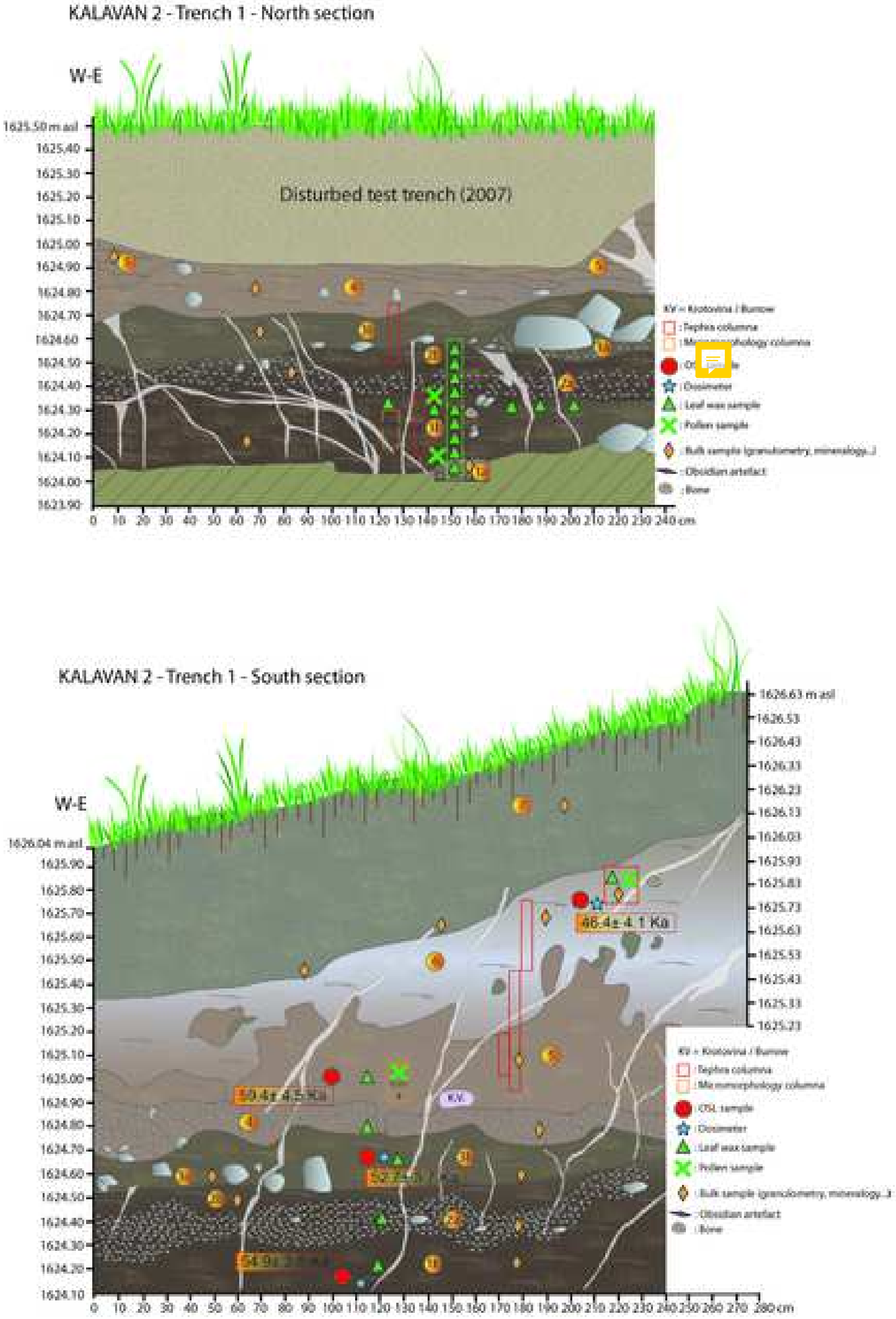

Figure

Click here to access/download;Figure;Kalavan 2 Fig. 5.jpg

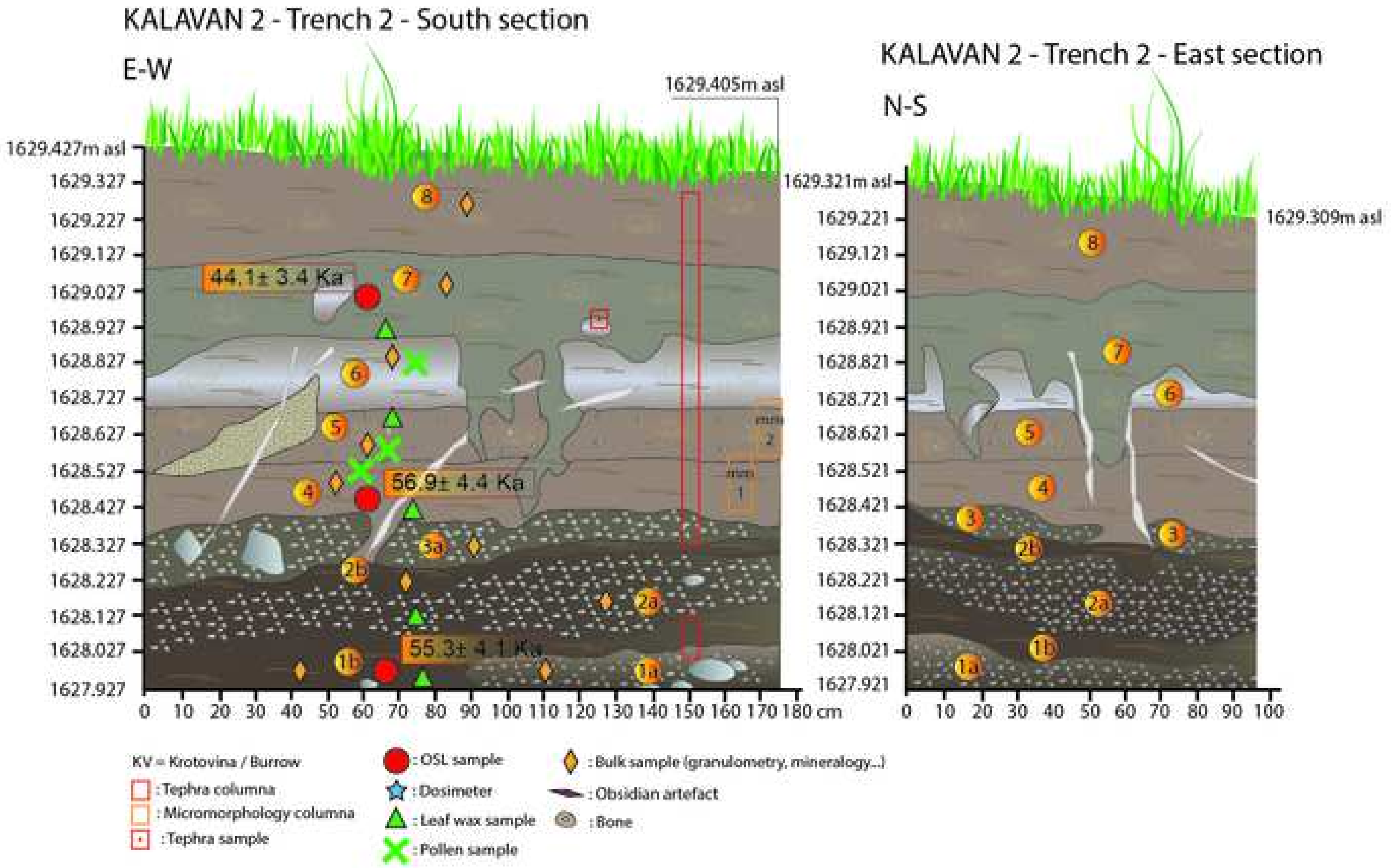

Figure
Click here to access/download;Figure;Kalavan 2 Fig. 6.jpg

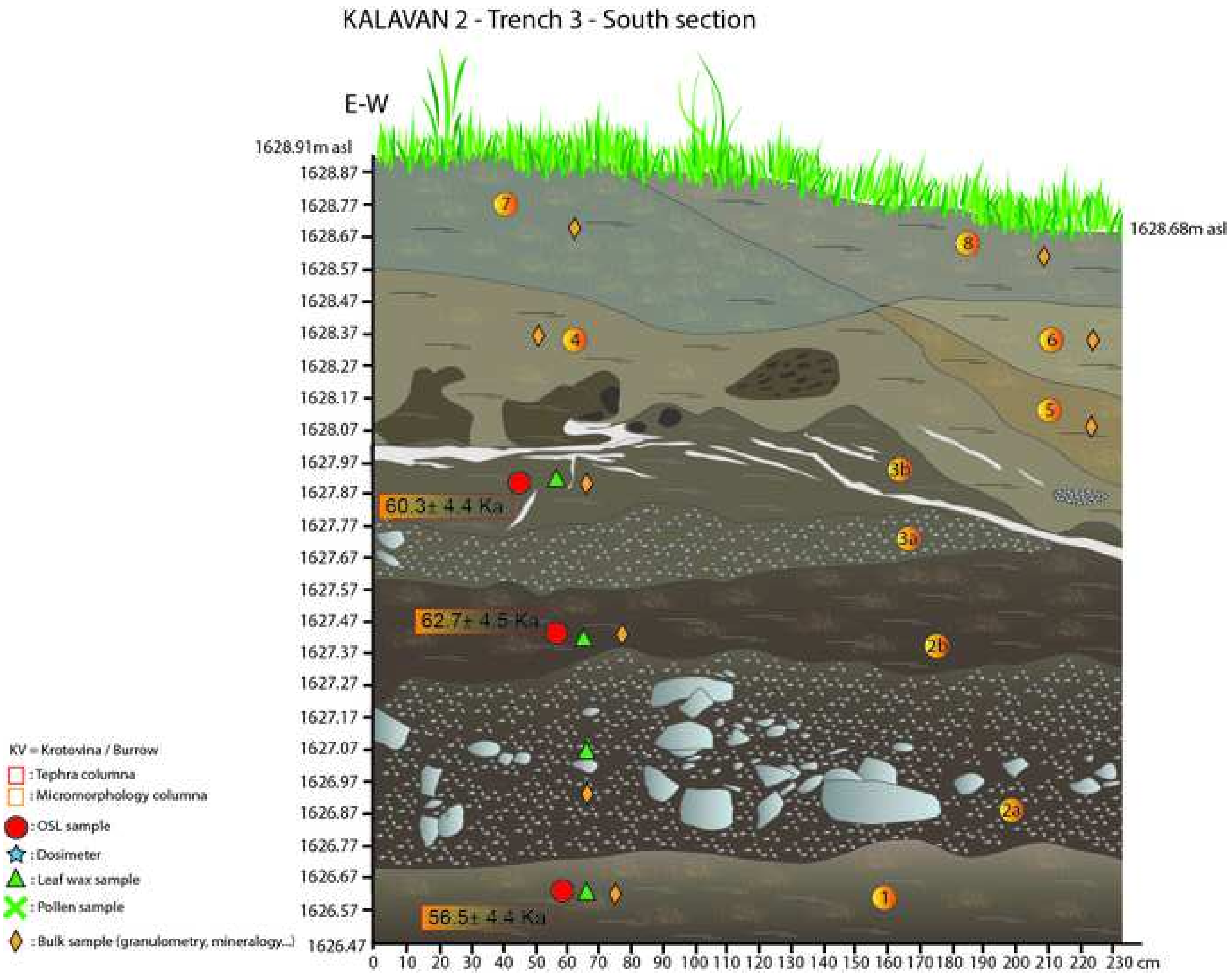

Figure

Click here to access/download;Figure;Kalavan 2 Fig. 7.jpg

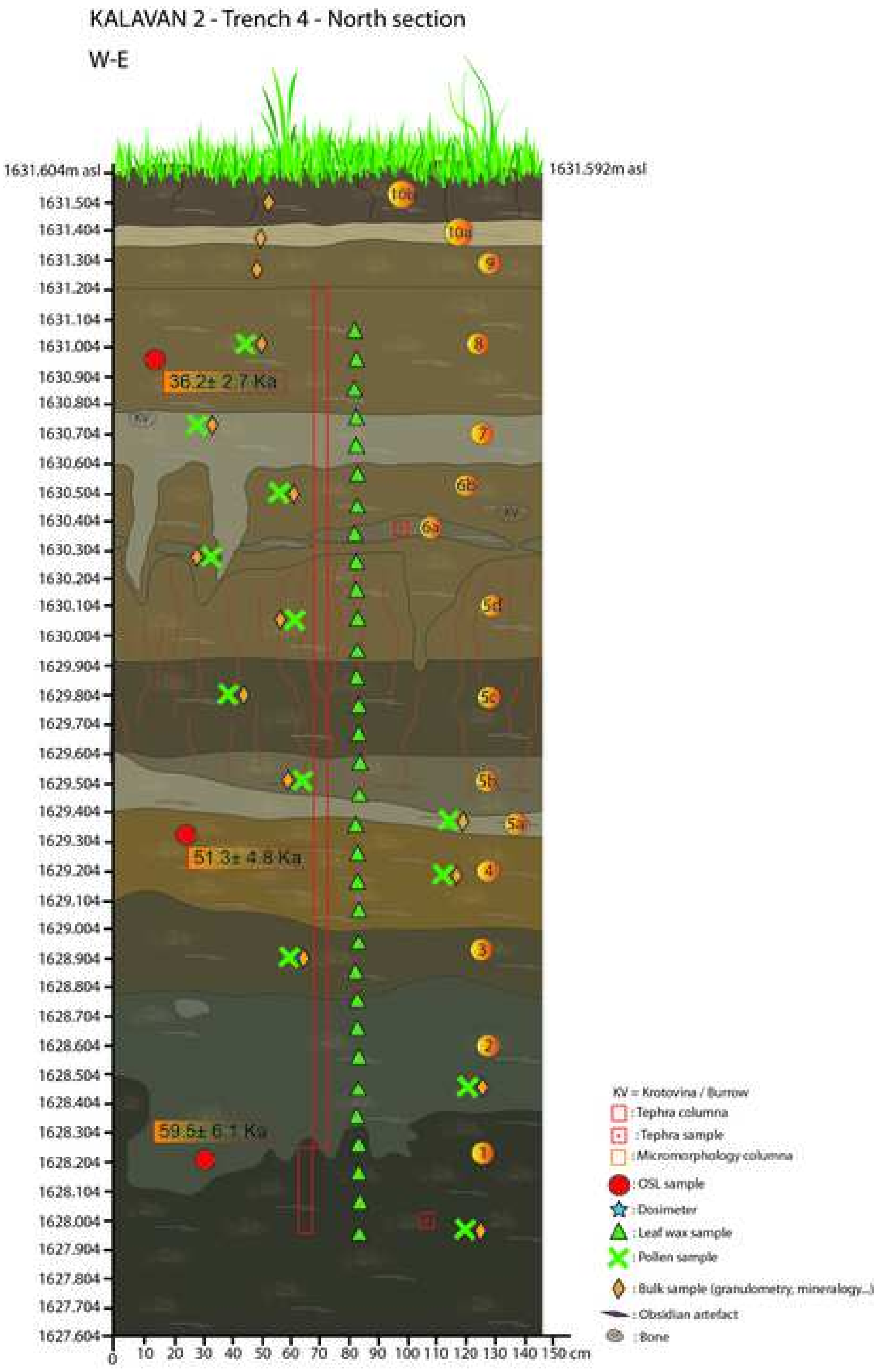

Click here to access/download;Figure;Kalavan 2 Fig. 8.jpg

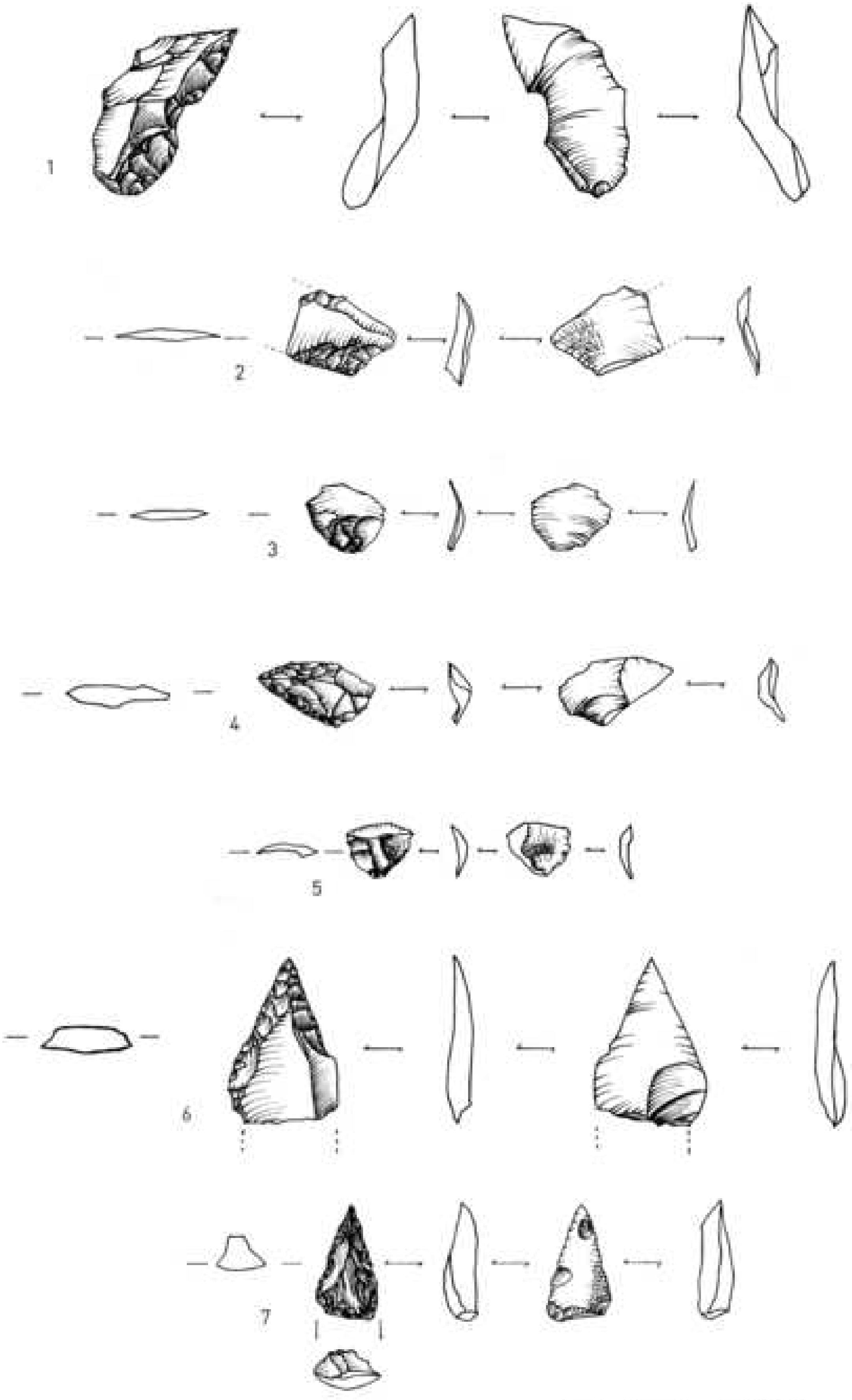

Click here to access/download;Figure;Kalavan 2 Fig. 9.jpg

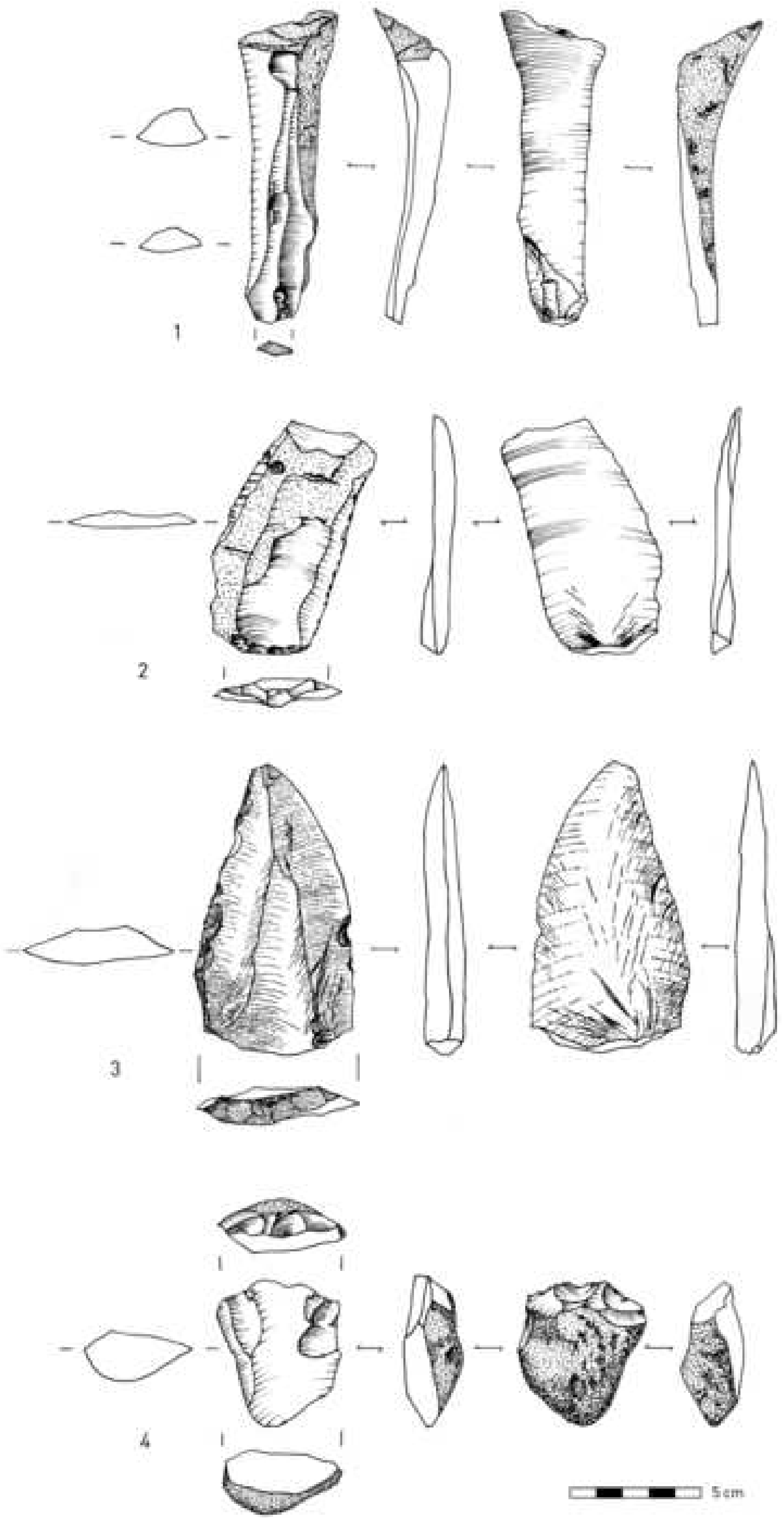

Click here to access/download;Figure;Kalavan 2 Fig. 10.jpg

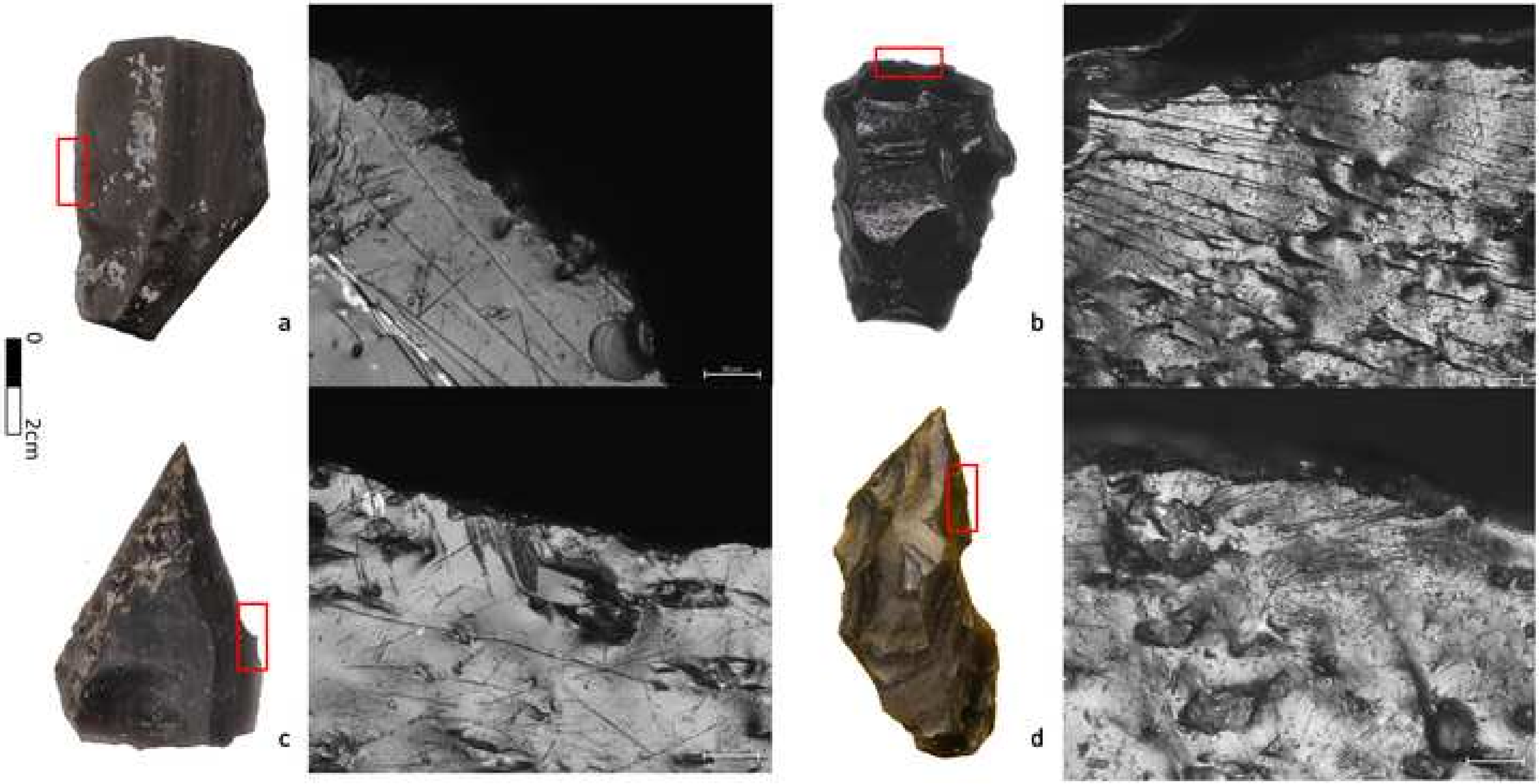

 Click here to access/download;Figure;Kalavan 2 fig. 11 percussive-tools.png

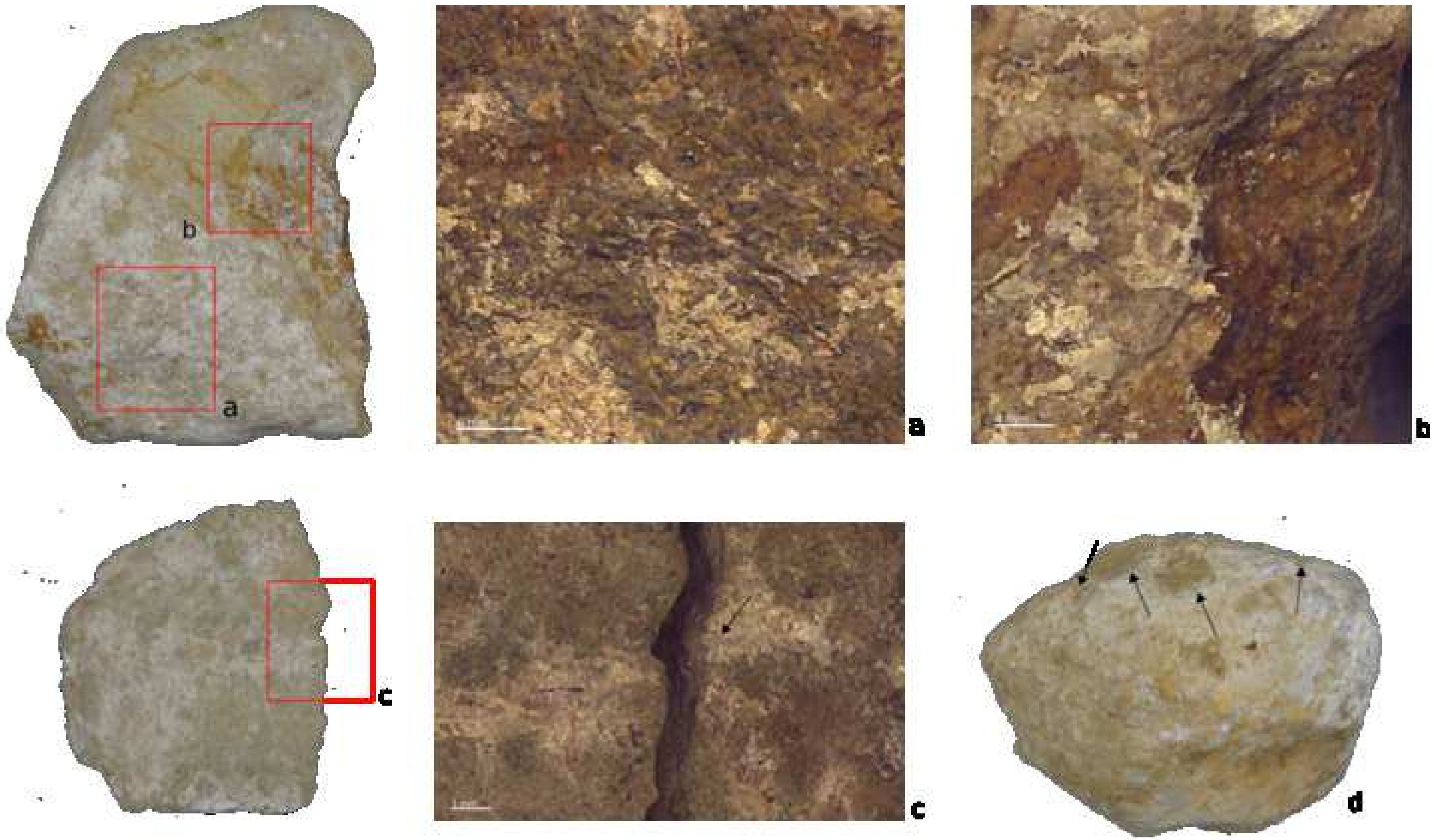

Click here to access/download;Figure;Kalavan 2 Fig. 12 obsidian sources map.pdf

Figure

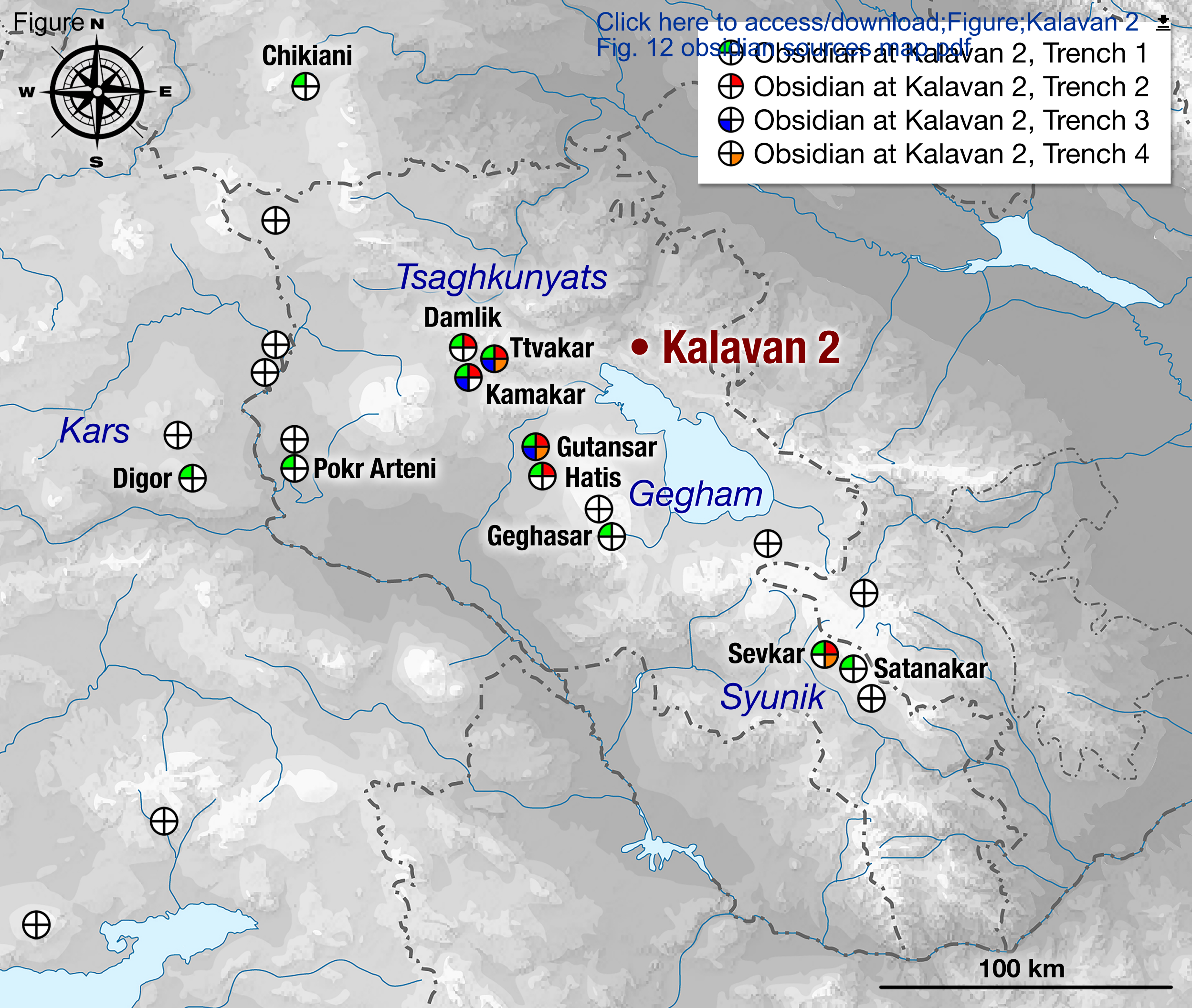

Figure

Click here to access/download;Figure;Kalavan 2 Fig. 13 sources by layer by trench.pdf

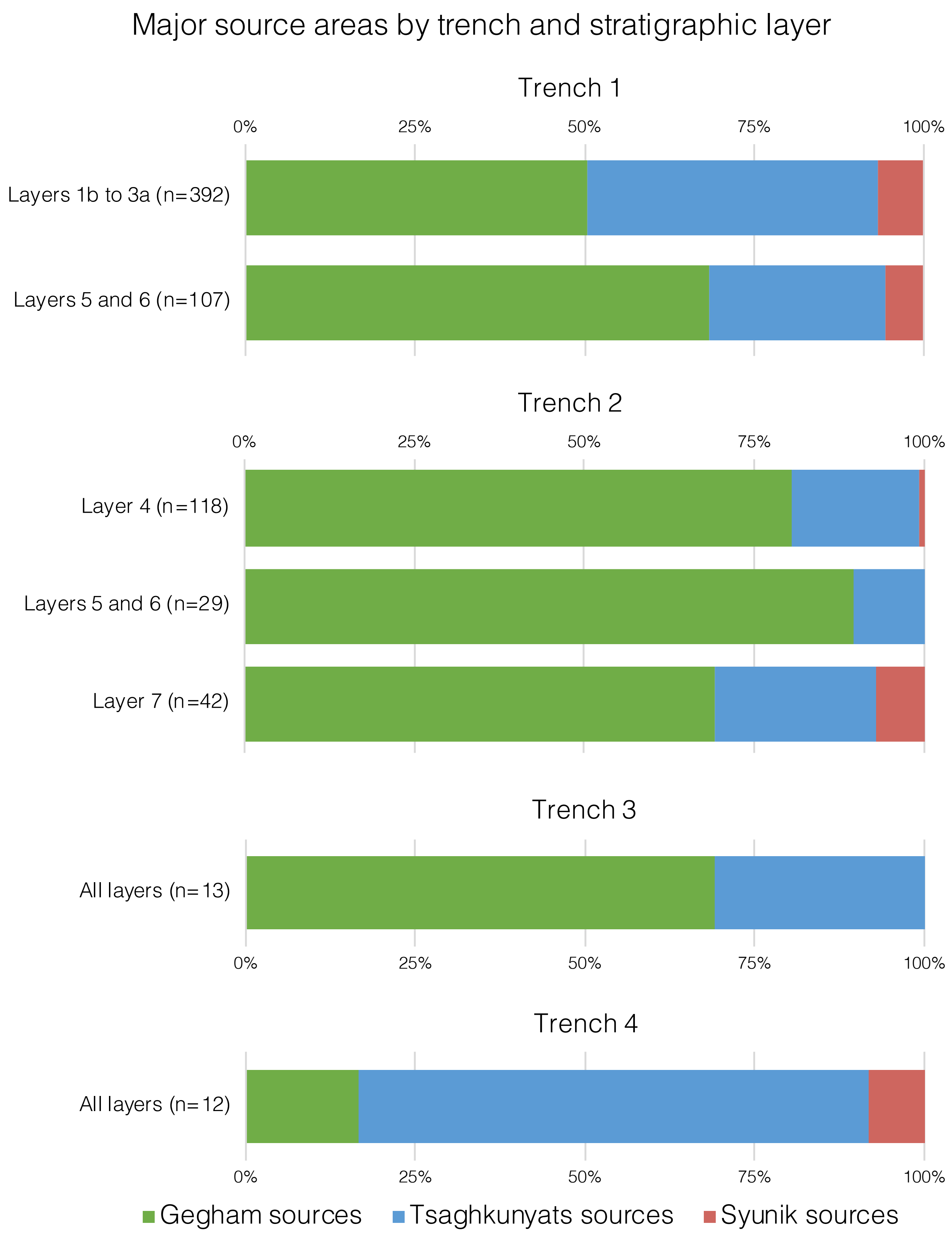

Click here to access/download;Figure;Kalavan 2 Fig. 14 sources by lithic class.pdf

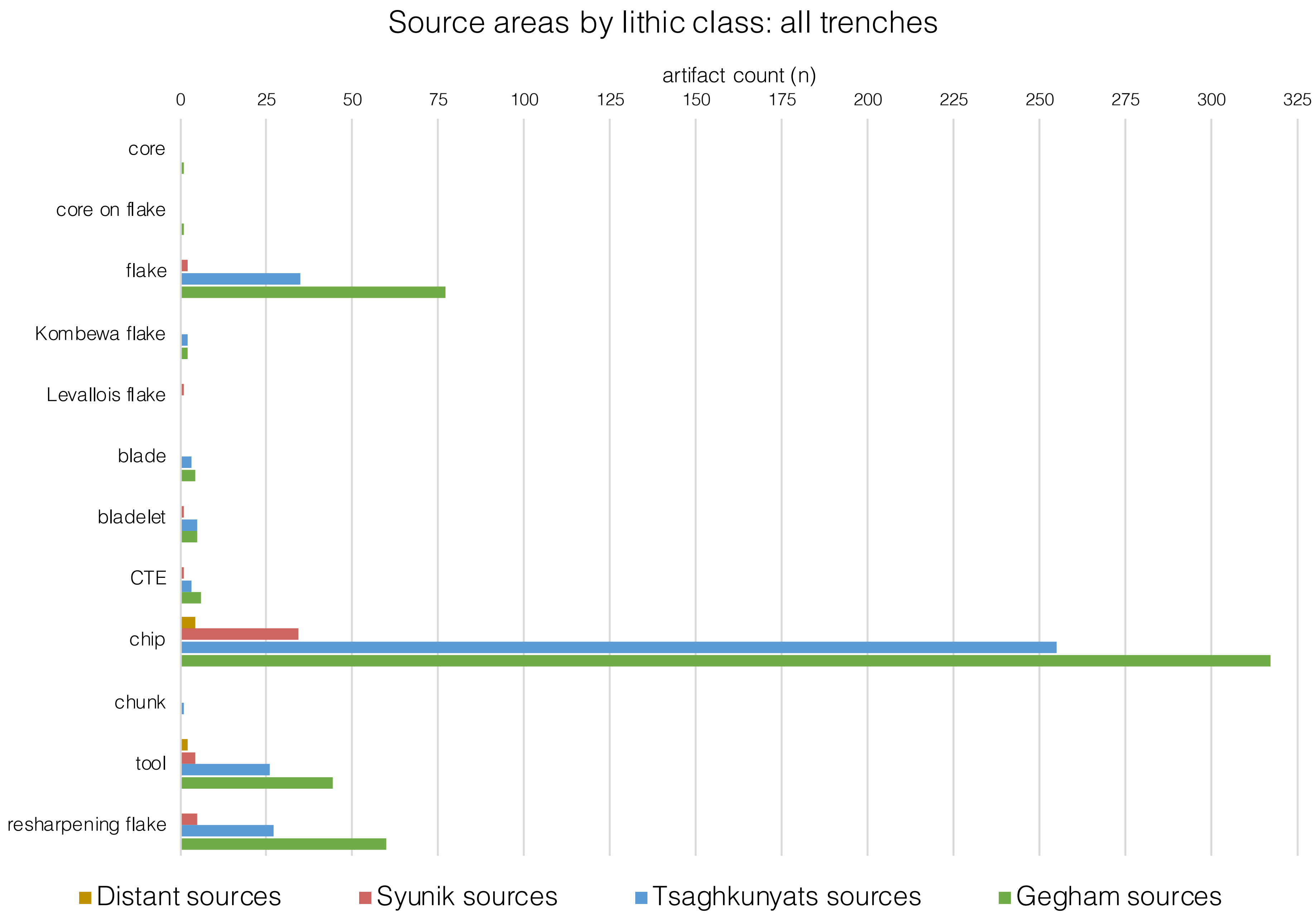

Figure

Click here to access/download;Figure;Kalavan 2 fig. 15 Kalavan 2 cut marked long bone.png

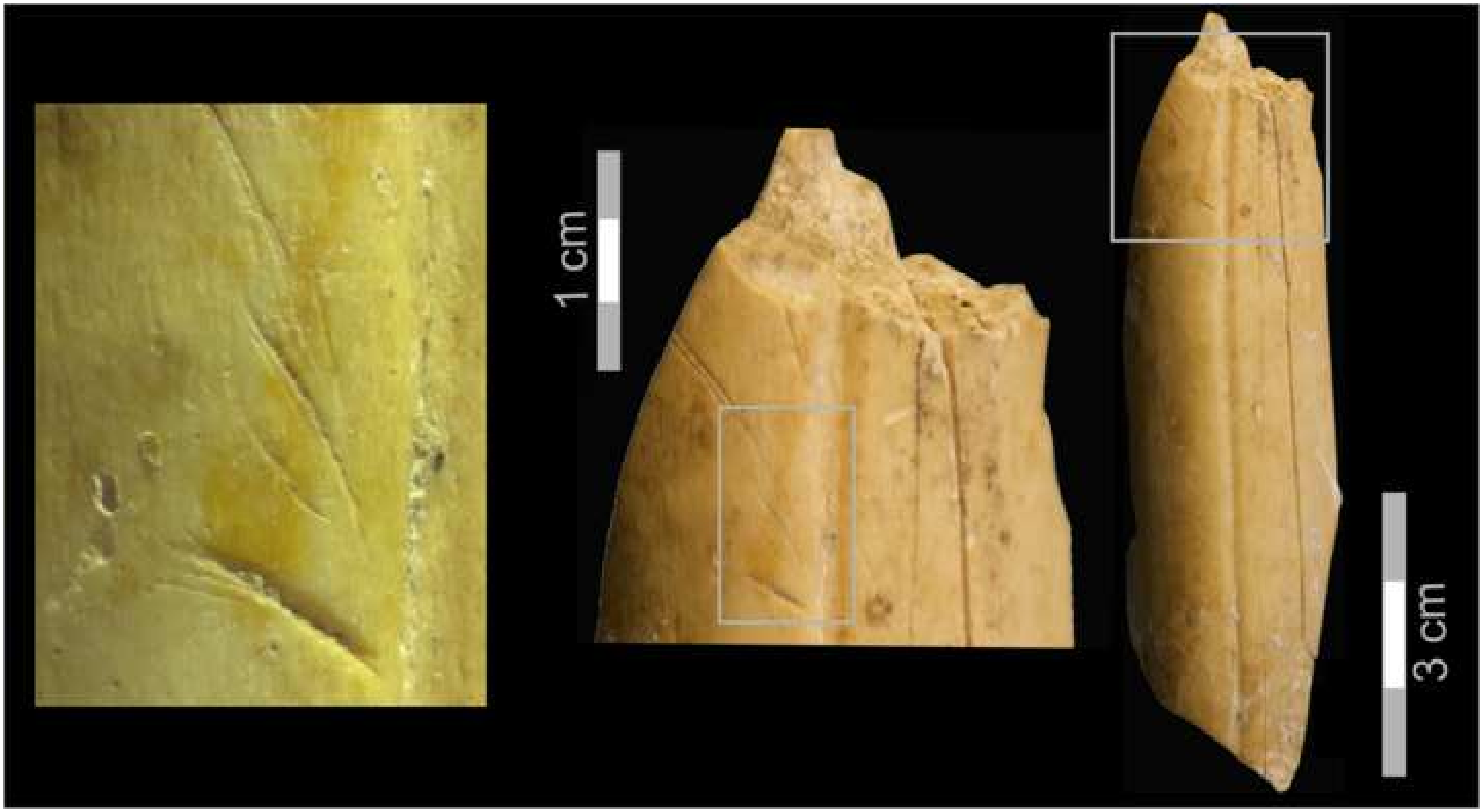

 Click here to access/download;Figure;Kalavan 2 Fig. 16.png

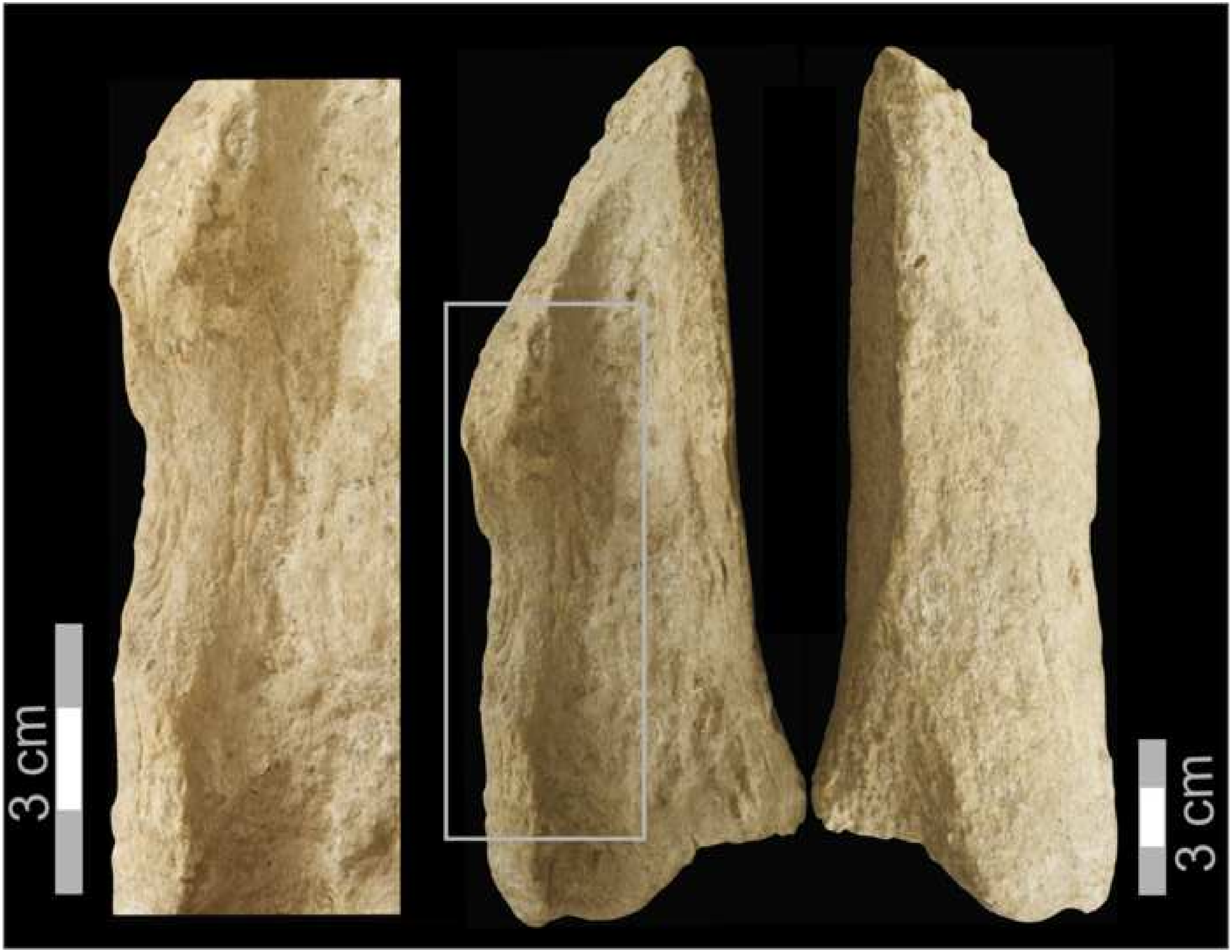

 Click here to access/download;Figure;Kalavan 2 Fig. 17.tif

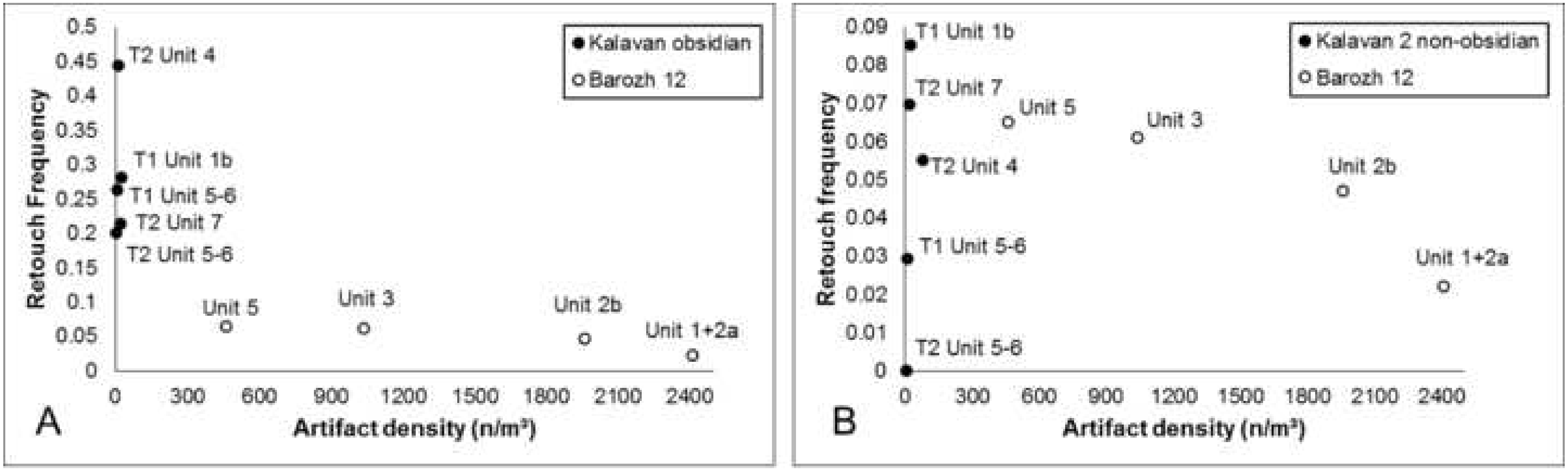

Click here to access/download
Supporting Information
Kalavan 2 Table 17.xlsx

Click here to access/download
Supporting Information
S4 Table 3 Small mammal postcranial elements per trench.docx

Click here to access/download
Supporting Information
Supporting information S1 dating.docx

Click here to access/download
Supporting Information
Supporting information S2 Sediment.docx

Click here to access/download
Supporting Information
Supporting information S3 use wear methodology.docx

Click here to access/download
Supporting Information
Supporting information S4 fauna.docx

Click here to access/download
Supporting Information
Sp 1 Fig. 1 pIRIR225-4-11-poly-nat-signal.JPG

Click here to access/download
Supporting Information
Sp 1 Fig. 2 pIRIR225-0.5mm-180-250-nat-signal.JPG

Click here to access/download
Supporting Information
Sp 1 Fig. 3 pIRIR225-1684-1685-1686.JPG

Click here to access/download
Supporting Information
Sp 1. Fig. 4 fading-pIRIR225_finegrain-1684-1685-1686.JPG

Click here to access/download
Supporting Information
Sp 1 fig. 5 Kalavan2_cryptotephrostratigraphy_trench 1 & 2.pdf

Click here to access/download

Supporting Information

Sp 1 fig. 6 Kalavan2_cryptotephrostratigraphy_trench 1 & 2.pdf

Click here to access/download
Supporting Information
Sp 1 fig. 7 visible tephra correlations.pdf

Click here to access/download
Supporting Information
Sp 2 fig. 1. pXRF results of elemental by trench.jpg

Click here to access/download
Supporting Information
Sp 2 fig. 2 Kalavan MM 3.1 figure.jpg

Click here to access/download
Supporting Information
Sp 2 fig. 3 Kalavan MM 3.2 figure.jpg

Click here to access/download
Supporting Information
Sp 2 fig. 4 Kalavan MM 4.1 figure.jpg

Click here to access/download
Supporting Information
Sp 2 Fig. 5 Kalavan MM 4.2 figure.jpg

Click here to access/download
Supporting Information
Sp 2 fig. 6 Kalavan MM 1.1 figure.jpg

Click here to access/download
Supporting Information
Sp 2 fig. 7 Kalavan MM 1.2 figure.jpg

Click here to access/download
Supporting Information
Sp 2 Fig. 8 Kalavan MM 2.1 figure.jpg

Click here to access/download

Supporting Information

Sp 2 fig. 9 Kalavan MM 2.2 figure.jpg

Click here to access/download
Supporting Information
Sp 2 fig. 10 ACL vs OEP-1.jpg

Click here to access/download
Supporting Information
Sp 3 Fig. 1 EF2 rev - element ratios sample.pdf

Click here to access/download
Supporting Information
Supplementary information figures caption.docx

Click here to access/download
Supporting Information
S1 table 1 Kalavan 2 tephra.docx

Click here to access/download
Supporting Information
S1 tephra_Kalavan.xlsx

Click here to access/download
Supporting Information
S2 Table 1 list of elements pXRF.docx

Click here to access/download
Supporting Information
S2 Table 3 pollen results.docx

Click here to access/download
Supporting Information
S3 list of studied samples for use-wear.docx

Click here to access/download
Supporting Information
S4 Table 1 fauna_size_class.docx

Click here to access/download
Supporting Information
S4 Table 2 meso and microwear.docx
