# Supplementary material for: Short-term occupations at high elevation during the Middle Paleolithic at Kalavan 2 (Republic of Armenia)
Source: PLoS One. 2021 Feb 4;16(2):e0245700. doi: 10.1371/journal.pone.0245700 (PMC7861461; doi:10.1371/journal.pone.0245700)
Supplement: Attachment — Submitted filename: PLOS Malinsky et al. Review.docx [file pone.0245700.s013.docx]

Review of “Short-term occupations at high elevation during the Middle Paleolithic at Kalavan 2 (Republic of Armenia)” by A. Malinsky-Muller et al.

Review by: Charles Egeland

I appreciate the opportunity to review this paper, which is an impressive multidisciplinary effort to understand the formation and paleoenvironment of a Middle Paleolithic site in Armenia. The southern Caucasus is a very important, but still under-researched, area, and data from additional sites, especially those associated with chronometric dates, are most welcome. The evidence for the exploitation of distant raw material sources is especially interesting. I won’t comment on the sedimentological or geomorphological analyses, as both are outside my area of expertise. Nevertheless, I would support publication, and as an interested reader I would appreciate if the authors could address the following issues:

1. It looks like there may be a repeated sentence in the first paragraph.
2. The authors argue that there was no hydrodynamic sorting of the lithics or the bones but, unlike the lithics, they provide no data on the size distribution of the faunal remains to support that claim. Were data on the orientation and inclination of the artifacts or faunal remains recorded?
3. The tables need to be standardized—some list the unit and/or trench while others do not.
4. In Table 18: is it possible to have 101% grass representation?
5. I think the authors’ concluding statement, that “[t]hese results from Kalavan 2 illustrate that high-elevation locales played a pivotal and integral role in late MP settlement dynamics and behavioral adaptations in the rugged landscapes of [the] Armenian highlands” is probably overstated. Do short-term occupations of one site imply a “pivotal role”? Even if we include other high-altitude sites in the area (e.g., Hovk 1), it seems premature (however reasonable) to conclude that such occupations were a key component of MP settlement-subsistence systems. I would refer to this as a working hypothesis rather than a broadly applicable conclusion.

Thanks again for this opportunity, and I would like to be named as a reviewer (Charles Egeland). I will happily communicate with the authors on any aspect of this review.
